# Functionalized 2,3′-Bipyrroles and Pyrrolo[1,2-*c*]imidazoles from Acylethynylpyrroles and Tosylmethylisocyanide

**DOI:** 10.3390/molecules29040885

**Published:** 2024-02-17

**Authors:** Maxim D. Gotsko, Ivan V. Saliy, Igor A. Ushakov, Lyubov N. Sobenina, Boris A. Trofimov

**Affiliations:** A.E. Favorsky Irkutsk Institute of Chemistry, Siberian Branch, Russian Academy of Sciences, 1 Favorsky Str., 664033 Irkutsk, Russia; gotsko@irioch.irk.ru (M.D.G.); saliy@irioch.irk.ru (I.V.S.); igor-papa71@mail.ru (I.A.U.); sobenina@irioch.irk.ru (L.N.S.)

**Keywords:** acylethynylpyrroles, tosylmethylisocyanide, cycloaddition, 2,3′-bipyrroles, pyrrolo[1,2-*c*]imidazoles

## Abstract

An efficient method for the synthesis of pharmaceutically prospective but still rare functionalized 2,3′-bipyrroles (in up to 80% yield) by the cycloaddition of easily available acylethynylpyrroles with tosylmethylisocyanide (TosMIC) has been developed. The reaction proceeds under reflux (1 h) in the KOH/THF system. In the *t*-BuONa/THF system, TosMIC acts in two directions: along with 2,3′-bipyrroles, the unexpected formation of pyrrolo[1,2-*c*]imidazoles is also observed (products ratio~1:1).

## 1. Introduction

The widespread applications of pyrroles, including their assemblies and the functionalized fused derivatives, in such fields as pharmacology, medicinal chemistry and materials science as well as their significance as precursors in the synthesis of many natural products have made this class of compounds one of the most important in heterocyclic chemistry [1,2,3,4,5,6,7,8,9,10,11,12].

Many products containing the pyrrole moiety exhibit a broad range of biological activities such as anticancer, antimicrobial, antiviral, anti-inflammatory, antipsychotic, antimalarial, insecticidal, etc. [1,2,3,4,5,6,7,8,9,10,11,12]. Based on pyrroles, anti-hyperlipidemic atorvastatin, anti-tumor agents sunitinib, vemurafenib, ruxolinitib and pemetrexed, vonoprazan used for the treatment of gastroduodenal ulcers, and nonsteroidal anti-inflammatory agents tolmetin, zomepirac and ketorolac were created.

In the light of the foregoing, it is clear that the synthesis of new pyrrole derivatives as potential drugs or that of their precursors is an important synthetic challenge.

Among the most promising precursors suitable for the synthesis of various pyrrole derivatives, acylethynylpyrroles are readily available products of the mechanochemical reaction of pyrroles with acylbromoacetylenes, which occurs by simple grinding the reagents in a mortar in the solid Al_2_O_3_ medium [13]. On the platform of these compounds, a wide range of pyrrole assemblies, such as pyrrolyl-oxazoles [14], pyrrolyl-pyrazoles [14], pyrrolyl-pyridines [14], pyrrolyl-pyrones [14] and pyrrolyl-pyridones [14], and condensed pyrrole compounds, such as pyrrolopyrazines [14], pyrrolizines [14] and pyrroloimidazoles [15], have already been obtained.

In this paper, we report on the methods for the synthesis of such important representatives of pyrrole assemblies and fused pyrroles as 2,3′-bipyrroles and pyrrolo[1,2-c]imidazoles via the reaction of 2-acylethynylpyrroles with tosylmethylisocyanide (TosMIC). 

**Scheme 1:**
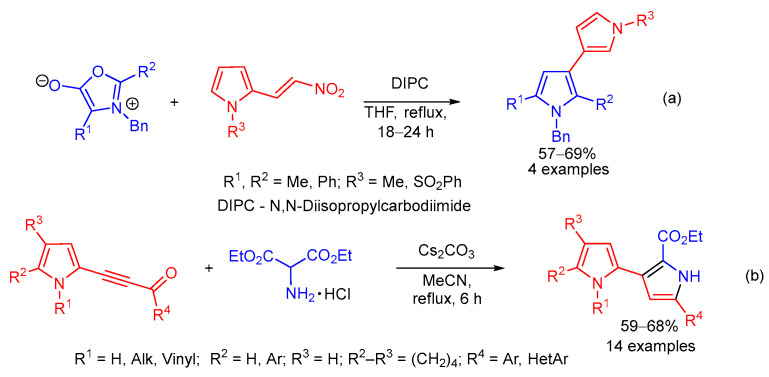
Synthetic approaches toward 2,3′-bipyrroles. (**a**) 1,3-Dipolar cycloaddition of unsymmetrical münchnones and nitrovinylheterocycles [17]; (**b**) Cycloaddition of acylethynylpyrroles with diethyl aminomalonate hydrochloride [18]. Previous works.

It is known that readily available 2,2′-bipyrroles are widely used as key building blocks in the synthesis of pyrrole macrocycles with different pharmacological activities [16]. Investigations of 2,3′-bipyrroles are still restricted by the absence of suitable facile approaches to these unique molecules. Only a few preparative significant methods for the synthesis of these assemblies are known. Among them are 1,3-dipolar cycloaddition of unsymmetrical münchnones and nitrovinylheterocycles in the presence of *N,N*′-diisopropylcarbodiimide (DIPC) (Scheme 1a) [17] and cycloaddition of acylethynylpyrroles with diethyl aminomalonate hydrochloride (Scheme 1b) [18]. 

Pyrrolo[1,2-*c*]imidazoles are an important structural motif frequently found in a number of natural products and bioactive molecules. For instance, some derivatives of pyrrolo[1,2-*c*]imidazole are capable of inhibiting aldose reductase [19], heparanase [20], glucosamine deacetylase (LpxC) [21], and ERK (extracellular signal-regulated kinases) [22]. 

Compounds of this class are antagonists of the 5-HT3 receptor with antiemetic activity especially effective in chemotherapy [23], the neuropeptide S receptor associated with biological processes such as appetite and food intake, movement, wakefulness, activation and anxiety [24], and the TNF receptor widely employed for the treatment of rheumatoid arthritis [25]. Pyrrolo[1,2-*c*]imidazoles also exhibit anticancer [26,27], antioxidant, antibacterial [28], anti-inflammatory and neuroprotective [29] activities.

**Figure 1:**
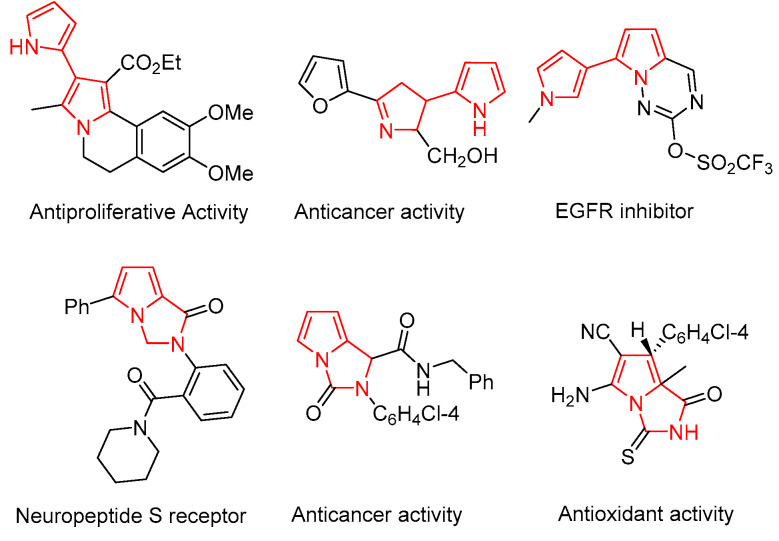
Some biological active compounds containing 2,3′-bipyrroles or pyrrolo[1,2-*c*]imidazoles moieties [24,26,28,30,31,32].

Despite the insufficient knowledge of the biological activity of 2,3′-bipyrroles, there is information about the prospects of their use as anticancer drugs[30,31] and EGFR inhibitor [32] (Figure 1).

It should be underlined that almost all pyrrolo[1,2-*c*]imidazoles studied to date contain an exocyclic carbonyl group or a thione function [19,20,21,22,23,24,25,26,27,28,29], which is probably explained by the inaccessibility of representatives of this class of compounds by other substituents.

**Scheme 2:**
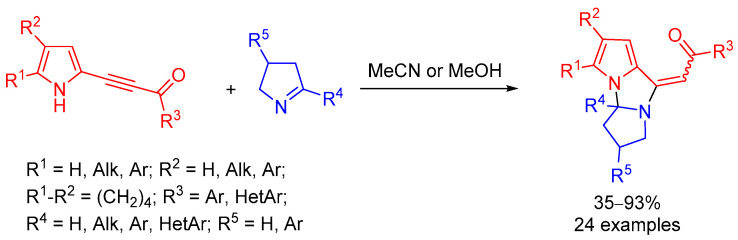
Synthesis of pyrrolo[1,2-*c*]imidazoles from acylethynylpyrroles and 1-pyrrolines.

Recently, based on the [3+2]-cycloaddition of acylethynylpyrroles to 1-pyrrolines, a method for the synthesis of pyrrolo[1,2-*c*]imidazoles with an exocyclic acylmethylidene group was developed (Scheme 2) [15]. 

## 2. Results and Discussion

In this paper, we share the results concerning the synthesis of 2,3′-bipyrroles and pyrrolo[1,2-*c*]imidazoles via the reaction of 2-acylethynylpyrroles **1a**–**o** with TosMIC, which is a versatile and powerful synthetic tool in heterocyclization reactions used to build five-membered heterocycles and heterofused systems [33].

It is known that activated acetylenes such as acetylenic esters or propiolic aldehydes react with TosMIC to afford pyrroles [34,35,36] or oxazoles [34,37], respectively, and carbonyl compounds can form oxazoles [34,38] or undergo reductive cyanation in nitriles [34,39]. 

At the same time, it was found that the reaction of 2-acylethynylpyrroles with TosMIC, depending on its conditions and substituents in the pyrrole ring, can deliver either pyrrolyl vinyl sulfones [40] or tosyl/pyrrolyl-capped 1,3-enynes [41]. In the former case, the reaction proceeds under a long reflux (120 h) in the Et_3_N/MeCN catalytic system, whereas for the easy and rapid (reflux, 1 h) synthesis of tosyl/pyrrolyl-capped 1,3-enynes, the *t*-BuOK/THF system is employed.

From these results, it follows that the optimal temperature for the reaction studied is that of reflux in THF, and the search for optimal conditions for the reaction of acylethynylpyrroles with TosMIC was carried out via reflux in THF at the molar ratio of acylethynylpyrroles:TosMIC:base = 1:2:2.

To commence the reaction of 2-acylethynylpyrroles **1a**–**o** with TosMIC, 2-benzoylethynylpyrrole (**1a**, 1 equiv) was refluxed with TosMIC (2 equiv) in THF in the presence of Et_3_N (2 equiv) for 24 h (Table 1, entry 1). However, under these conditions, not even traces of any products were found in the reaction mixture. Other organic catalysts, such as DBU (entry 2) and DABCO (entry 3), also did not promote the reaction: in all cases, the starting reagents were completely returned from the reaction.

Carrying out a reaction of 2-benzoylethynylpyrrole **1a** with TosMIC in the K_2_CO_3_/THF system (reflux, 24 h, entry 4), we were encountered, to our surprise, with a new reaction of this popular reagent: along with the expected 2,3′-bipyrrole **2a** (15% isolated yield), pyrrolo[1,2-*c*]imidazole, which functionalized with acyl and tosyl substituents, (*E*)-1-phenyl-2-(1*H*-pyrrolo[1,2-*c*]imidazol-1-ylidene)-3-tosylpropan-1-one (**3a**), was formed (isolated yield is 10%). 

When other bases, e.g., Cs_2_CO_3_, NaOH, KOH, *t*-BuONa, *t*-BuOK (entries 5–9, 11), were employed in the reaction of TosMIC with ethynylpyrrole **1a**, the complete conversion of the latter was achieved after 1 h of reflux in THF.

The highest yield of bipyrrole **2a** (80%) was reached when the reaction was carried out in the presence of KOH (entry 7) or in the presence of *t*-BuOK (entry 11). Under these conditions, pyrroloimidazole **3a** was detected only in traces. In preparative quantities, pyrroloimidazole **3a** was formed only in the *t*-BuONa/THF system: in this case, bipyrrole **2a** and pyrroloimidazole **3a** were obtained in almost equal ratios (^1^H NMR data), with their isolated yields being 27 and 37%, respectively (entry 9).

The selective formation of pyrroloimidazole **3a** was observed when the reaction was carried out in the presence of NaH (molar ratio **1a**:TosMIC:NaH = 1:1:1 and 1:2:2). However, in this case, the reaction was accompanied by strong tarring, and the target product was isolated in a yield of 39–45% (Table 1, entries 13, 14).

As follows from the data in Table 1, the selectivity of the reaction and yields of the products depend both on the nature and ratio of bases. This can be rationalized from the points of strength of bases and their softness (hardness) according to Pearson’s HSAB principle [42,43]. Indeed, in the reaction mixture, two different acids, namely CH-acid (TosMIC) and NH-acid (the pyrrole moiety), compete with each other for a base.

In the real process, the strengths of bases and their softness (hardness) as well as those of the acids depend not only on the nature of the base/alkali metal cation but also on the solvation effects, which are different for Na and K cations—i.e., the selectivity of the reaction and yields of the products are complex functions of several mutually depended variables.

**Scheme 3:**
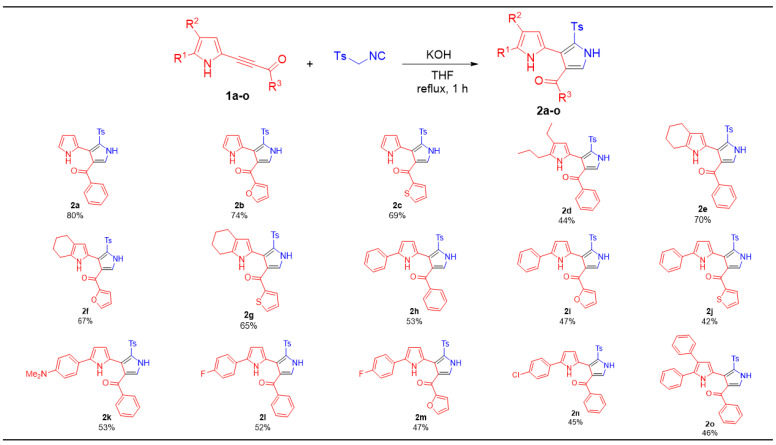
The synthesis of 2,3′-bipyrroles **2a**–**o** from 2-(acylethynyl)pyrroles **1a**–**o** and TosMIC (scope of the reaction) ^a^. ^a^ Reagents and conditions: acylethynylpyrrole **1a**–**o** (1 mmol), TosMIC (2 mmol), KOH (2 mmol), THF (10 mL) and reflux, 1 h.

Next, given the better convenience of working with KOH than with *t*-BuOK and its lower cost, we evaluated the substrate scope of the synthesis of bipyrroles **2a**–**o** in the presence of KOH (Scheme 3). 

The experiments showed that the reaction of 2-acylethynylpyrroles **1a**–**o** with TosMIC under these conditions proceeded efficiently and selectively, thus providing a short route to 2,3-dipyrroles **2a**–**o**, which are still rare and poorly studied compounds, in good yields up to 80%.

As follows from Scheme 3, the reaction is tolerant to different acylethynylpyrroles with aliphatic and aromatic substituents in the pyrrole ring as well as 2-acylethynyl-4,5,6,7-tetrohydroindoles; i.e., the synthesis has quite a general character.

Next, a range of acylethynylpyrroles (acylethynylpyrroles **1a**,**b**,**d**,**h**–**n**) were involved in the reaction with TosMIC in the *t*-BuONa/THF system to evaluate the scope of the novel cyclization giving hitherto unknown pyrrolo[1,2-*c*]imidazoles **3a**–**j**. 

**Scheme 4:**
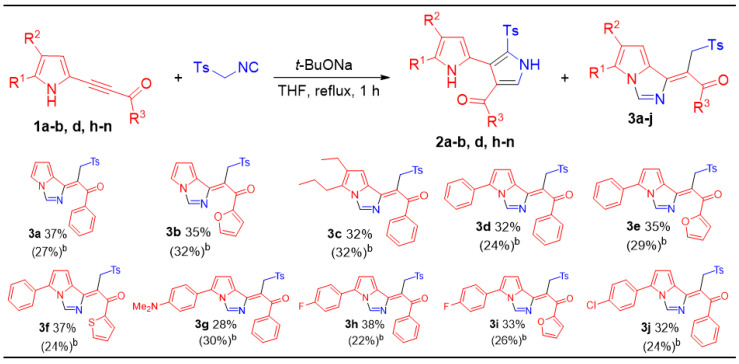
The synthesis of pyrrolo[1,2-*c*]imidazoles **3a**–**j** from 2-(acylethynyl)pyrroles **1a**,**b**,**d**,**h**–**n** and TosMIC (scope of the reaction) ^a^. ^a^ Reagents and conditions: acylethynylpyrrole **1a**,**b**,**d**,**h**–**n** (1 mmol), TosMIC (2 mmol), *t*-BuONa (2 mmol), THF (10 mL) and reflux, 1 h; ^b^ The yield of the corresponding bipyrrole is presented in parentheses.

Unfortunately, in contrast to the KOH/THF system, which selectively catalyzed the formation of 2,3′-bipyrroles, the *t*-BuONa/THF system c promoted the formation of both 2,3′-bipyrroles and pyrrolo[1,2-*c*]imidazoles from all the studied acylethynylpyrroles **1a**,**b**,**d**,**h**–**n** (Scheme 4). The reaction products were easily separated via column chromatography (SiO_2_, *n*-hexane:diethyl ether, 5:1).

As can be seen from Scheme 4, this reaction, like the formation of 2,3′-bipyrroles, also tolerates different acylethynylpyrroles with aliphatic and aromatic substituents in the pyrrole ring as well as 2-acylethynyl-4,5,6,7-tetrohydroindoles—i.e., it also has quite a general character.

The structures of compounds **2a** and **3a** were proved by ^1^H and ^13^C NMR spectroscopy using also 2D COSY, NOESY, HMBC and HSQC techniques. The ^1^H NMR spectra of 2,3′-bipyrroles contain broad singlets of 2 NH groups at 10.23–11.77 and 9.84–10.77 ppm, while the spectra of pyrroloimidazole **3a** show singlets of a proton at the C=N bond at 8.51–8.91 ppm and methylene group at 5.05–5.20 ppm.

**Scheme 5:**
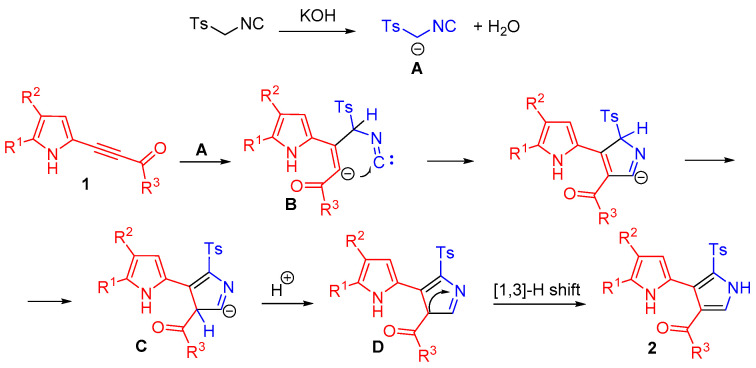
Proposed route of 2,3′-bipyrroles **2** formation.

Apparently, the formation of 2,3′-bipyrroles **2** starts with proton abstraction from the CH_2_-group of TosMIC followed by a nucleophilic attack of carbanion **A**, thus generated as a C-nucleophile, on the triple bond of acylethynylpyrroles **1** to afford intermediate anion **B**. This anion then undergoes intramolecular cyclization with the participation of an anionic center and a carbene carbon atom to form the cyclic anion **C** that is further quenched by a proton of the medium. The aromatization of the intermediate bipyrrole **D**, as a result of the [1,3]-H shift finishes the process (Scheme 5).

**Scheme 6:**
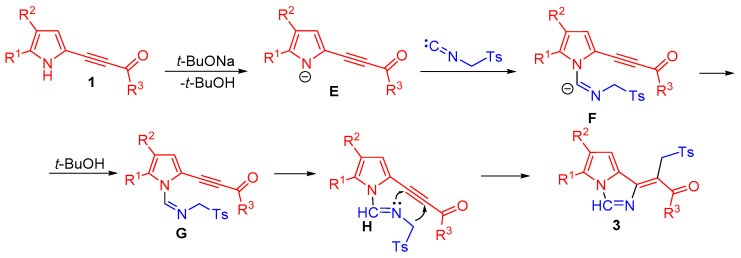
Proposed route of pyrrolo[1,2-*c*]imidazoles **3** formation.

The assembly of pyrrolo[1,2-*c*]imidazoles **3** likely involves the formation of the pyrrolate anion **E** in the first stage due to the greater deprotonating ability of the *t*-BuONa/THF super-base system compared to the KOH/THF system. The anion **E** attacks the carbene carbon atom of TosMIC, and the forming carbanion **F** is neutralized with *t*-BuOH to give intermediate imine **G**. The latter undergoes intramolecular cyclization to pyrroloimidazole via the four-membered transition state **H**, wherein the cleavage of the N and CH_2_-Ts bond occurs accompanied by simultaneous C1-N and C2-CH_2_Ts bonding (Scheme 6). 

## 3. Materials and Methods

### 3.1. General Information

IR spectra were obtained with a Vertex 70 spectrometer (Bruker, Billerica, MA, USA, 400–4000 cm^–1^, films). ^1^H (400.13 MHz) and ^13^C (100.6 MHz) spectra were recorded on a DPX-400 spectrometer (Bruker, Chicago, IL, USA) at ambient temperatures in CDCl_3_ and DMSO-*d*_6_ solutions and referenced to CDCl_3_ (residual protons of CDCl_3_ in ^1^H NMR δ = 7.26 ppm; ^13^C NMR δ = 77.1 ppm) and DMSO-*d*_6_ (residual protons of CDCl_3_ in ^1^H NMR δ = 2.50 ppm; ^13^C NMR δ = 39.52 ppm).

The C, H, N microanalyses were performed on a Flash EA 1112 CHNS-O/MAS analyzer (CHN Analyzer, Thermo Fisher Scientific, Monza, Italy). Sulfur was determined via complexometric titration with Chlorasenazo III. Fluorine content was determined on a SPECOL 11 (Carl Zeiss, Jena, Germany) spectrophotometer. Clorine was determined using mercurimetric titration. The melting point (uncorrected) was determined through a Kofler micro hot-stage apparatus (Antwerpen, Belgium). High-resolution mass spectral analyses were performed on an acetonitrile solution with 0.1% HFBA on an HPLC Agilent 1200/Agilent 6210 TOF instrument equipped with an electrospray ionization (ESI) source (Agilent, Santa Clara, CA, USA). 

All reactions were carried out in air.

Acylethynylpyrroles **1a**–**o** were prepared from corresponding pyrroles and acylbromoacetylenes in the presence of Al_2_O_3_ according to the reported procedure [13], and TosMIC, *t*-BuONa, KOH, NaH and THF were the commercial products.

### 3.2. The Reaction of Acylethynylpyrroles ***1a**–**o*** with TosMIC in the Presence of KOH: Synthesis of 2,3′-Bipyrroles (***2a**–**o***) (General Procedure)

A solution of acylethynylpyrrole **1a**–**o** (1 mmol) and TosMIC (395 mg, 2 mmol) in THF (10 mL) was heated to reflux. Then, under reflux, a suspension of KOH·0.5H_2_O (130 mg, 2 mmol) in THF (10 mL) was added dropwise to the reaction mixture for 1 h. The residue, after removal of the solvent, was fractionated using column chromatography (SiO_2_, *n*-hexane:diethyl ether, 5:1) to afford the 2,3′-bipyrrole **2a**–**o**. 

### 3.3. The Reaction of Acylethynylpyrroles ***1a**,**b**,**d**,**h**–**n*** with TosMIC in the Presence of t-BuONa: Synthesis of 2,3′-Bipyrroles (***2a**,**b**,**d**,**h**–**n***) and Pyrrolo[1,2-c]imidazoles ***3a**–**j*** (General Procedure)

A solution of acylethynylpyrrole **1b**,**d**,**h**–**n** (1 mmol) and TosMIC (395 mg, 2 mmol) in THF (10 mL) was heated to reflux. Then, under reflux, a solution of *t*-BuONa (224 mg, 2 mmol) in THF (10 mL) was added dropwise to the reaction mixture for 1 h. The residue, after removal of the solvent, was fractionated using column chromatography (SiO_2_, *n*-hexane:diethyl ether, 5:1) to afford the 2,3′-bipyrrole **2a**,**b**,**d**,**h**–**n** (SiO_2_, *n*-hexane:diethyl ether, 5:1) pyrrolo[1,2-*c*]imidazoles **3a**–**j**. Yields of 2,3′-bipyrrole **2a**,**b**,**d**,**h**–**n**, thus obtained, are as follows: **2a**—27%; **2b**—32%; **2d**—32%; **2h**—24%; **2i**—29%, **2j**—24%; **2k**—30%; **2l**—22%; **2m**—26%; and **2n**—24%.

### 3.4. Characterization Data of 2,3-Bipyrroles ***2a**–**o*** and Pyrrolo[1,2-c]imidazoles ***3a**–**j***

Phenyl(2′-tosyl-1*H*,1′*H*-[2,3′-bipyrrol]-4′-yl)methanone (**2a**).

Yield 312 mg (80%). Yellow crystals, mp 163–165 °C. IR (KBr, cm^−1^): 3295 (NH), 3148, 3063 (=CH), 2920 (CH), 1636 (CO), 1597, 1517 (C=C), 1354, 1140 (SO_2_). ^1^H NMR (400.13 MHz, CDCl_3_): *δ* 10.89 (br.s, 1H, NH, CO-pyrrole), 9.92 (br.s, 1H, NH, pyrrole), 7.75–7.73 (m, 2H, Ph), 7.59–7.57 (m, 2H, Ph), 7.54–7.52 (m, 1H, Ph), 7.44–7.40 (m, 2H, Ph), 7.24 (d, *J* = 3.5 Hz, 1H, H-5, CO-pyrrole), 7.16–7.14 (m, 2H, Ph), 6.84–6.83 (m, 2H, H-5, H-3, pyrrole), 6.20–6.18 (m, 1H, H-4, pyrrole), 2.34 (s, 3H, CH_3_). ^13^C NMR (100.6 MHz, CDCl_3_): *δ* 192.4, 144.7, 139.3, 137.5, 132.7, 129.8 (2C), 129.7 (2C), 129.3, 128.4 (2C), 127.2 (2C), 125.2, 124.3, 123.0, 121.4, 119.2, 113.1, 109.5, 21.7. Anal. Calcd for C_22_H_18_N_2_O_3_S: C, 67.68; H, 4.65; N, 7.17; S, 8.21%. Found: C, 67.84; H, 4.78; N, 7.32; S, 8.01%. HRMS (ESI-TOF): found 391,1116. Calcd. for [C_22_H_18_N_2_O_4_S+H]^+^ 391,1121.

Furan-2-yl(2′-tosyl-1*H*,1′*H*-[2,3′-bipyrrol]-4′-yl)methanone (**2b**).

Yield 282 mg (74%). Yellow crystals, mp 95–97 °C. IR (KBr, cm^−1^): 3293 (NH), 3131 (=CH), 2923, 2859 (CH), 1620 (CO), 1563, 1520 (C=C), 1354, 1140 (SO_2_). ^1^H NMR (400.13 MHz, CDCl_3_): *δ* 10.71 (br.s, 1H, NH, CO-pyrrole), 9.94 (br.s, 1H, NH, pyrrole), 7.68 (d, *J* = 3.3 Hz, 1H, H-5, CO-pyrrole), 7.62–7.61 (m, 1H, H-5, furan), 7.57–7.55 (m, 2H, Ph), 7.16–7.14 (m, 3H, H-3, furan, Ph), 6.86–6.84 (m, 2H, H-3, H-5, pyrrole), 6.54 (dd, *J* = 3.3, 1.4 Hz, 1H, H-4, furan), 6.22–6.20 (m, 1H, H-4, pyrrole), 2.33 (s, 3H, CH_3_). ^13^C NMR (100.6 MHz, CDCl_3_): *δ* 177.8, 153.4, 146.9, 144.7, 137.9, 129.7 (2C), 127.5, 127.3 (2C), 125.7, 123.3, 123.0, 121.4, 119.8, 119.3, 113.2, 112.5, 109.5, 21.6. Anal. Calcd for C_20_H_16_N_2_O_4_S: C, 63.15; H, 4.24; N, 7.36; S, 8.43%. Found: C, 63.27; H, 4.42; N, 7.53; S, 8.3%.

Thiophen-2-yl(2′-tosyl-1*H*,1′*H*-[2,3′-bipyrrol]-4′-yl)methanone (**2c**).

Yield 274 mg (69%). Yellow crystals, mp 175–177 °C. IR (KBr, cm^−1^): 3351 (NH), 3137, 3110 (=CH), 2922 (CH), 1614 (CO), 1569, 1520, 1484, 1458 (C=C), 1359, 1136 (SO_2_). ^1^H NMR (400.13 MHz, CDCl_3_): *δ* 10.64 (br.s, 1H, NH, CO-pyrrole), 10.16 (br.s, 1H, NH, pyrrole), 7.65 (d, *J* = 4.9 Hz, 1H, H-5, thiophene), 7.57 (d, *J* = 8.1 Hz, 2H, Ph), 7.54 (d, *J* = 3.5 Hz, 1H, H-3, thiophene), 7.46 (d, *J* = 3.3 Hz, 1H, H-3, pyrrole), 7.14 (d, *J* = 8.1 Hz, 2H, Ph), 7.09–7.07 (m, 1H, H-4, thiophene), 6.85–6.83 (m, 1H, H-4, pyrrole), 6.83–6.82 (m, 1H, H-3, pyrrole), 6.21–6.20 (m, 1H, H-5, pyrrole), 2.32 (s, 3H, CH_3_). ^13^C NMR (100.6 MHz, CDCl_3_): *δ* 183.5, 145.0, 144.8, 137.5, 134.6, 134.4, 129.7 (2C), 128.2, 127.4, 127.2 (2C), 125.4, 124.1, 122.5, 121.4, 119.3, 113.0, 109.4, 21.7. Anal. Calcd for C_20_H_16_N_2_O_3_S_2_: C, 60.59; H, 4.07; N, 7.07; S, 16.17%. Found: C, 60.73; H, 4.27; N, 7.18; S, 15.99%. HRMS (ESI-TOF): found 397,0681. Calcd. for [C_20_H_16_N_2_O_3_S_2_+H]^+^ 397,0685.

(4-Ethyl-5-propyl-2′-tosyl-1*H*,1′*H*-[2,3′-bipyrrol]-4′-yl)(phenyl)methanone (**2d**).

Yield 203 mg (44%). Yellow crystals, mp 170–172 °C. IR (KBr, cm^−1^): 3285 (NH), 3160 (=CH), 2958, 2927, 2869 (CH), 1630 (CO), 1596, 1575, 1519, 1495, 1461 (C=C), 1351, 1142, (SO_2_). ^1^H NMR (400.13 MHz, CDCl_3_): *δ* 10.63 (br.s, 1H, NH, CO-pyrrole), 9.84 (br.s, 1H, NH, pyrrole), 7.72–7.70 (m, 2H, Ph), 7.63–7.61 (m, 2H, Ph), 7.54–7.51 (m, 1H, Ph), 7.43–7.39 (m, 2H, Ph), 7.22 (d, *J* = 3.5 Hz, 1H, H-5, CO-pyrrole), 7.16–7.14 (m, 2H, Ph), 6.67–6.65 (m, 1H, pyrrole), 2.55–2.53 (m, 2H, CH_2_), 2.51–2.53 (m, 2H, CH_2_), 2.37 (s, 3H, CH_3_), 1.67–1.57 (m, 2H CH_2_), 1.12 (t, *J* = 7.6 Hz, 3H, CH_3_), 0.90 (t, *J* = 7.3 Hz, 3H, CH_3_). ^13^C NMR (100.6 MHz, CDCl_3_): *δ* 192.5, 144.5, 139.8, 137.7, 132.4, 130.1, 129.7 (2C), 129.6 (2C), 129.5, 128.4 (2C), 127.3 (2C), 124.1, 124.0, 123.8, 123.2, 118.7, 113.5, 28.0, 23.1, 21.7, 19.0, 16.1, 13.9. Anal. Calcd for C_27_H_28_N_2_O_3_S: C, 70.41; H, 6.13; N, 6.08; S, 6.96%. Found: C, 70.6; H, 6.28; N, 6.25; S, 6.84%. HRMS (ESI-TOF): found 461,1899. Calcd. for [C_27_H_28_N_2_O_3_S +H]^+^ 461,1902.

Phenyl(4-(4,5,6,7-tetrahydro-1*H*-indol-2-yl)-5-tosyl-1*H*-pyrrol-3-yl)methanone (**2e**). 

Yield 311 mg (70%). Yellow crystals, mp 235–237 °C. IR (KBr, cm^−1^): 3342 (NH), 3171 (=CH), 2922, 2851 (CH), 1633 (CO), 1594, 1574, 1521, 1495 (C=C), 1351, 1126 (SO_2_). ^1^H NMR (400.13 MHz, CDCl_3_): *δ* 10.60 (br.s, 1H, NH, CO-pyrrole), 9.89 (br.s, 1H, NH, pyrrole), 7.73–7.71 (m, 2H, Ph), 7.65–7.63 (m, 2H, Ph), 7.56–7.52 (m, 1H, Ph), 7.44–7.40 (m, 2H, Ph), 7.20–7.19 (m, 2H, Ph), 7.17–7.15 (m, 1H, H-2, CO-pyrrole), 6.68–6.67 (m, 1H, H-3, pyrrole), 2.61–2.59 (m, 2H, CH_2–_7), 2.51–2.48 (m, 2H, CH_2–_4), 2.36 (s, 3H, CH_3_), 1.79–1.78 (m, 2H, CH_2–_5), 1.73–1.71 (m, 2H, CH_2–_6). ^13^C NMR (100.61 MHz, CDCl_3_): *δ* 192.5, 144.5, 139.8, 137.7, 132.5, 129.7 (3C), 129.6 (2C), 129.2, 128.4 (2C), 127.2 (2C), 124.1, 123.9, 121.3, 119.5, 118.8, 112.4, 24.0, 23.6, 23.1, 23.1, 21.7. Anal. Calcd for C_26_H_24_N_2_O_3_S: C, 70.25; H, 5.44; N, 6.30; S, 7.21%. Found: C, 70.40; H, 5.25; N, 6.44; S, 7.04%. HRMS (ESI-TOF): found 445,1586. Calcd. for [C_26_H_24_N_2_O_3_S+H]^+^ 445,1589.

Furan-2-yl(4-(4,5,6,7-tetrahydro-1*H*-indol-2-yl)-5-tosyl-1*H*-pyrrol-3-yl)methanone (**2f**). 

Yield 291 mg (67%). Yellow crystals, mp 173–175 °C. IR (KBr, cm^−1^): 3278 (NH), 3019 (=CH), 2924, 2850 (CH), 1618 (CO), 1609, 1524, 1464 (C=C), 1351, 1138 (SO_2_). ^1^H NMR (400.13 MHz, CDCl_3_): *δ* 10.39 (br.s, 1H, NH, CO-pyrrole), 10.27 (br.s, 1H, NH, pyrrole), 7.65–7.64 (m, 2H, Ph), 7.62–7.61 (m, 2H, H-5, H-3, furan), 7.16–7.14 (m, 2H, Ph), 7.12 (d, *J* = 3.5 Hz, 1H, H-2, CO-pyrrole), 6.64 (d, *J* = 2.2 Hz, 1H, H-3, pyrrole), 6.52 (dd, *J* = 3.5, 1.6 Hz, 1H, H-4, furan), 2.60–2.57 (m, 2H, CH_2–_7), 2.51–2.48 (m, 2H, CH_2–_4), 2.33 (s, 3H, CH_3_), 1.81–1.75 (m, 2H, CH_2–_5), 1.74–1.70 (m, 2H, CH_2–_6). ^13^C NMR (100.6 MHz, CDCl_3_): *δ* 177.9, 153.2, 147.0, 144.5, 137.7, 129.6 (2C), 129.1, 128.5, 127.2 (2C), 124.0, 123.8, 122.4, 119.9, 119.3, 118.6, 112.4, 112.3, 24.0, 23.5, 23.1, 23.0, 21.7. Anal. Calcd for C_24_H_22_N_2_O_4_S: C, 66.34; H, 5.10; N, 6.45; S, 7.38%. Found: C, 66.41; H, 5.87; N, 6.18; S, 7.22%. HRMS (ESI-TOF): found 435,1379. Calcd. for [C_24_H_22_N_2_O_4_S+H]^+^ 435,1383.

(4-(4,5,6,7-Tetrahydro-1*H*-indol-2-yl)-5-tosyl-1*H*-pyrrol-3-yl)(thiophen-2-yl)methanone (**2g**). 

Yield 293 mg (65%). Yellow crystals, mp 198–200 °C. IR (KBr, cm^−1^): 3289 (NH), 3131 (=CH), 2924, 2851 (CH), 1613 (CO), 1595, 1514 (C=C), 1358, 1140 (SO_2_). ^1^H NMR (400.13 MHz, CDCl_3_): *δ* 10.23 (br.s, 1H, NH, CO-pyrrole), 10.02 (br.s, 1H, NH, pyrrole), 7.66–7.62 (m, 3H, Ph, H-5, thiophene), 7.56–7.55 (m, 1H, H-3, thiophene), 7.43 (d, *J* = 3.4 Hz, 1H, H-2, CO-pyrrole), 7.17–7.15 (m, 2H, Ph), 7.10–7.08 (m, 1H, H-4 thiophene), 6.63–6.60 (m, 1H, H-3, pyrrole), 2.60–2.57 (m, 2H, CH_2–_7), 2.51–2.48 (m, 2H, CH_2–_4), 2.35 (s, 3H, CH_3_), 1.84–1.76 (m, 2H, CH_2–_5), 1.74–1.69(m, 2H, CH_2–_6). ^13^C NMR (100.61 MHz, CDCl_3_): *δ* 183.5, 145.3, 144.5, 137.7, 134.3, 134.1, 129.6 (2C), 129.2, 128.1, 127.8, 127.3 (2C), 124.3, 123.7, 123.4, 119.4, 118.6, 112.3, 24.0, 23.6, 23.1, 23.0, 21.7. Anal. Calcd for C_24_H_22_N_2_O_3_S_2_: C, 63.98; H, 4.92; N, 6.22; S, 14.23%. Found: C, 63.75; H, 5.07; N, 6.08; S, 14.12%. HRMS (ESI-TOF): found 451,1150. Calcd. for [C_24_H_22_N_2_O_3_S_2_+H]^+^ 451,1154.

Phenyl(5-phenyl-2′-tosyl-1*H*,1′*H*-[2,3′-bipyrrol]-4′-yl)methanone (**2h**). 

Yield 247 mg (53%). Yellow crystals, mp 205–207 °C. IR (KBr, cm^−1^): 3281 (NH), 3147 (=CH), 2923 (CH), 1629 (CO), 1597, 1677, 1508, 1477 (C=C), 1349, 1139 (SO_2_). ^1^H NMR (400.13 MHz, CDCl_3_): *δ* 11.77 (br.s, 1H, NH, CO-pyrrole), 9.89 (br.s, 1H, NH, pyrrole), 7.78–7.76 (m, 2H, Ph), 7.68–7.66 (m, 2H, Ph), 7.65–7.63 (m, 2H, Ph), 7.58–7.54 (m, 1H, Ph), 7.46–7.43 (m, 2H, Ph), 7.40–7.36 (m, 2H, Ph), 7.28 (d, *J* = 3.2 Hz, 1H, H-5, CO-pyrrole), 7.21–7.19 (m, 1H, Ph), 7.18–7.16 (m, 2H, Ph), 7.04–7.02 (m, 1H, H-4, pyrrole), 6.56–6.55 (m, 1H, H-3, pyrrole), 2.32 (s, 3H, CH_3_). ^13^C NMR (100.6 MHz, CDCl_3_): *δ* 192.7, 144.9, 139.6, 137.4, 133.1, 132.7, 132.6, 129.9 (2C), 129.8 (2C), 129.0 (2C), 128.5 (2C), 127.2 (2C), 126.4, 125.1, 124.2, 123.9 (2C), 122.8, 122.8, 122.7, 120.2, 114.9, 107.4, 21.7. Anal. Calcd for C_28_H_22_N_2_O_3_S: C, 72.08; H, 4.75; N, 6.00; S, 6.87%. Found: C, 71.81; H, 4.85; N, 5.89; S, 6.75%. HRMS (ESI-TOF): found 467,1429. Calcd. for [C_28_H_22_N_2_O_3_S+H]^+^ 467,1431.

Furan-2-yl(5-phenyl-2′-tosyl-1*H*,1′*H*-[2,3′-bipyrrol]-4′-yl)methanone (**2i**). 

Yield 215 mg (47%). Yellow crystals, mp 90–92 °C. IR (KBr, cm^−1^): 3278 (NH), 3150 (=CH), 2923 (CH), 1619 (CO), 1519, 1561, 1531, 1509 (C=C), 1351, 1140 (SO_2_). ^1^H NMR (400.13 MHz, CDCl_3_): *δ* 11.58 (br.s, 1H, NH of CO-pyrrole), 7.70–7.66 (m, 2H, H-5, furan, H-5, CO-pyrrole), 7.64–7.62 (m, 4H, Ph), 7.39–7.35 (m, 2H, Ph), 7.21–7.17 (m, 1H, Ph), 7.15–7.12 (m, 3H, H-3, furan, Ph), 7.05–7.03 (m, 1H, H-3, pyrrole), 6.57–5.55 (m, 1H, H-4, pyrrole), 6.52 (dd, *J* = 3.4, 1.5 Hz, 1H, H-4, furan), 2.29 (s, 3H, CH_3_). ^13^C NMR (100.6 MHz, CDCl_3_): *δ* 178.0, 153.0, 147.2, 144.8, 137.4, 132.9, 132.5, 129.8 (2C), 129.1, 129.0 (2C), 127.1 (2C), 126.3, 125.1, 123.8 (2C), 122.8 (2C), 122.6, 120.4, 114.7, 112.5, 107.3, 21.6. Anal. Calcd for C_26_H_20_N_2_O_4_S: C, 68.41; H, 4.42; N, 6.14; S, 7.02%. Found: C, 68.11; H, 4.29; N, 6.22; S, 6.91%. HRMS (ESI-TOF): found 457,1222. Calcd. for [C_26_H_20_N_2_O_4_S+H]^+^ 457,1220.

(5-Phenyl-2′-tosyl-1*H*,1′*H*-[2,3′-bipyrrol]-4′-yl)(thiophen-2-yl)methanone (**2j**). 

Yield 198 mg (42%). Yellow crystals, mp 105–107 °C. IR (KBr, cm^−1^): 3295 (NH), 3137 (=CH), 2922, 2853 (CH), 1613 (CO), 1582, 1562, 1511 (C=C), 1359, 1140 (SO_2_). ^1^H NMR (400.13 MHz, CDCl_3_): *δ* 11.26 (br.s, 1H, NH, CO-pyrrole), 9.72 (br.s, 1H, NH, pyrrole), 7.68–7.67 (m, 1H, H-5 of thiophene), 7.65–7.60 (m, 5H, Ph, H-3, thiophene), 7.50–7.49 (m, 1H, H-5, CO-pyrrole), 7.40–7.36 (m, 2H, Ph), 7.22–7.18 (m, 1H, Ph), 7.16–7.14 (m, 2H, Ph), 7.12–7.10 (m, 1H, H-4, thiophene), 6.97 (dd, *J* = 3.6, 2.5 Hz, 1H, H-3, pyrrole), 6.55–6.54 (m, 1 7.2, 134.5, 134.3, 132.9, 132.4, 129.7 (2C), 128.9 (2C), 128.0, 127.6, 127.0 (2C), 126.2, 125.1, 123.9, 123.7 (2C), 122.5, 122.1, 114.5, 107.1, 21.5. Anal. Calcd for C_26_H_20_N_2_O_3_S_2_: C, 66.08; H, 4.27; N, 5.93; S, 13.57%. Found: C, 66.25; H, 4.46; N, 6.07; S, 13.38%. HRMS (ESI-TOF): found 473,0994. Calcd. for [C_26_H_20_N_2_O_3_S_2_+H]^+^ 473,0998.

(5-(4-(Dimethylamino)phenyl)-2′-tosyl-1*H*,1′*H*-[2,3′-bipyrrol]-4′-yl)(phenyl)methanone (**2k**). 

Yield 270 mg (53%). Yellow crystals, mp 213–215 °C. IR (KBr, cm^−1^): 3240 (NH), 2924, 2853 (CH), 1613 (CO), 1512, 1491 (C=C), 1350, 1141 (SO_2_). ^1^H NMR (400.13 MHz, CDCl_3_): *δ* 11.58 (br.s, 1H, NH, CO-pyrrole), 10.03 (br.s, 1H, NH, pyrrole), 7.75–7.73 (m, 2H, Ph), 7.67–6.65 (m, 2H, Ph), 7.54–7.52 (m, 3H, Ph), 7.44–7.40 (m, 2H, Ph), 7.24–7.19 (m, 1H, H-5, CO-pyrrole), 7.16–7.14 (m, 2H, Ph), 6.98–6.96 (m, 1H, H-4, pyrrole), 6.78–6.76 (m, 2H, Ph), 6.40–6.38 (m, 1H, H-3 pyrrole), 2.96 (s, 6H, N(CH_3_)_2_), 2.31 (s, 3H, CH_3_). ^13^C NMR (100.6 MHz, CDCl_3_): *δ* 192.6, 149.4, 144.7, 139.7, 137.6, 133.9, 132.5, 130.0, 129.8 (2C), 129.7 (2C), 128.4 (2C), 127.1 (3C), 125.0 (2C), 124.3, 123.9, 123.2, 121.6, 121.3, 114.9, 113.2, 105.6, 40.8 (2C), 21.7. Anal. Calcd for C_30_H_27_N_3_O_3_S: C, 70.71; H, 5.34; N, 8.25; S, 6.29%. Found: C, 70.82; H, 5.48; N, 8.38; S, 6.18%. HRMS (ESI-TOF): found 510,1851. Calcd. for [C_30_H_27_N_3_O_3_S+H]^+^ 510,1848.

(5-(4-Fluorophenyl)-2′-tosyl-1*H*,1′*H*-[2,3′-bipyrrol]-4′-yl)(phenyl)methanone (**2l**).

Yield 252 mg (52%). Yellow crystals, mp 130–132 °C. IR (KBr, cm^−1^): 3233 (NH), 3063 (=CH), 2969 (CH), 1613 (CO), 1597, 1486 (C=C), 1351, 1145 (SO_2_). ^1^H NMR (400.13 MHz, DMSO-d_6_): *δ* 11.79 (br.s, 1H, NH, CO-pyrrole), 9.94 (br.s, 1H, NH, pyrrole), 7.77–7.75 (m, 2H, Ph), 7.68–7.66 (m, 2H, Ph), 7.60–7.55 (m, 3H, Ph), 7.47–7.43 (m, 2H, Ph), 7.28 (d, *J* = 3.5 Hz, 1H, H-5, CO-pyrrole), 7.19–7.17 (m, 2H, Ph), 7.09–7.03 (m, 3H, H-4, pyrrole, Ph), 6.49–6.47 (m, 1H, H-3, pyrrole), 2.33 (s, 3H, CH_3_). ^13^C NMR (100.6 MHz DMSO-d_6_): *δ* 190.4, 160.5 (d, *J* = 242.4 Hz), 144.1, 138.8 (d, *J* = 29.4 Hz, 2C), 131.9, 130.9, 130.1, 129.8 (2C), 129.3 (d, *J* = 2.8 Hz), 129.0 (2C), 128.1 (2C), 126.7, 126.5 (2C), 125.3 (d, *J* = 7.8 Hz, 2C), 123.6, 122.5, 121.7, 115.6, 115.4, 112.7, 106.2, 21.0. Anal. Calcd for C_28_H_21_FN_2_O_3_S: C, 69.41; H, 4.37; F, 3.92; N, 5.78; S, 6.62%. Found: C, 69.56; H, 4.50; N, 5.95; S, 6.51; F, 4.04%.

(5-(4-Fluorophenyl)-2′-tosyl-1*H*,1′*H*-[2,3′-bipyrrol]-4′-yl)(furan-2-yl)methanone (**2m**). 

Yield 228 mg (48%). Yellow crystals, mp 118–120 °C. IR (KBr, cm^−1^): 3281 (NH), 3152 (=CH), 2923, 2856 (CH), 1621 (CO), 1602, 1561, 1512, 1488 (C=C), 1351, 1141 (SO_2_). ^1^H NMR (400.13 MHz, CDCl_3_): *δ* 11.58 (br.s, 1H, NH, CO-pyrrole), 7.72–7.70 (m, 1H, H-5, furan), 7.67–7.65 (m, 2H, Ph), 7.64–7.62 (m, 1H, H-3, furan), 7.59–7.55 (m, 2H, Ph), 7.17 (d, *J* = 3.6 Hz, 1H, H-5, CO-pyrrole), 7.16–7.14 (m, 2H, Ph), 7.08–7.03 (m, 3H, H-3 pyrrole, Ph), 6.54 (dd, *J* = 3.3, 1.5 Hz, 1H, H-4, furan), 6.49–6.48 (m, 1H, H-4 pyrrole), 2.31 (s, 3H, CH_3_). ^13^C NMR (100.6 MHz, CDCl_3_): *δ* 178.0, 161.7 (d, *J* = 245.4 Hz), 153.1, 147.3, 144.9, 137.5, 132.2, 129.8 (2C), 129.0, 128.9 (d, *J* = 2.8 Hz), 127.2 (2C), 125.5 (d, *J* = 7.9 Hz, 2C), 125.1, 122.8, 122.7, 122.7, 120.5, 115.9 (d, *J* = 21.8 Hz, 2C), 114.8, 112.6, 107.1, 21.7. Anal. Calcd for C_26_H_19_FN_2_O_4_S: C, 65.81; H, 4.04; F, 4.00; N, 5.90; S, 6.76%. Found: C, 65.99; H, 4.21; F, 4.18; N, 6.09; S, 6.66%. HRMS (ESI-TOF): found 475,1128. Calcd. for [C_26_H_19_FN_2_O_4_S+H]^+^ 475,1133.

(5-(4-Chlorophenyl)-2′-tosyl-1*H*,1′*H*-[2,3′-bipyrrol]-4′-yl)(phenyl)methanone (**2n**). 

Yield 225 mg (45%). Yellow crystals, mp 218–220 °C. IR (KBr, cm^−1^): 3275 (NH), 3118, 3092 (=CH), 1630 (CO), 1596, 1576, 1505, 1477 (C=C), 1351, 1145 (SO_2_). ^1^H NMR (400.13 MHz, CDCl_3_): *δ* 11.83 (br.s, 1H, NH, CO-pyrrole), 9.89 (br.s, 1H, NH, pyrrole), 7.78–7.76 (m, 2H, Ph), 7.68–7.66 (m, 2H, Ph), 7.59–7.54 (m, 3H, Ph), 7.47–7.43 (m, 2H, Ph), 7.34–7.32 (m, 2H, Ph), 7.29 (d, *J* = 3.5 Hz, 1H, H-5, CO-pyrrole), 7.19–7.17 (m, 2H, Ph), 7.05 (dd, *J* = 3.6, 2.4 Hz, 1H, H-4, pyrrole), 6.54–6.52 (m, 1H, H-3, pyrrole), 2.33 (s, 3H, CH_3_). ^13^C NMR (100.61 MHz, CDCl_3_): *δ* 192.8, 144.9, 139.6, 137.4, 132.7, 131.8, 131.7, 131.1, 130.3, 129.9 (2C), 129.8 (2C), 129.2 (2C), 128.5 (2C), 127.1 (2C), 125.1, 125.0 (2C), 124.0, 123.2, 122.6, 114.9, 107.8, 21.7. Anal. Calcd for C_28_H_21_ClN_2_O_3_S: C, 67.13; H, 4.23; Cl, 7.08; N, 5.59; S, 6.40%. Found: C, 67.33; H, 4.38; N, 5.70; S, 6.29%. HRMS (ESI-TOF): found 501,1040. Calcd. for [C_28_H_21_ClN_2_O_3_S+H]^+^ 501,1044.

(4,5-Diphenyl-2′-tosyl-1*H*,1′*H*-[2,3′-bipyrrol]-4′-yl)(phenyl)methanone (**2o**). 

Yield 250 mg (46%). Yellow crystals, mp 203–205 °C. IR (KBr, cm^−1^): 3296 (NH), 3059 (=CH), 2923 (CH), 1632 (CO), 1599, 1508, 1477 (C=C), 1351, 1132 (SO_2_). ^1^H NMR (400.13 MHz, CDCl_3_): *δ* 11.36 (br.s, 1H, NH, CO-pyrrole), 9.82 (br.s, 1H, NH, pyrrole), 7.80–7.77 (m, 2H, Ph), 7.73–7.70 (m, 2H, Ph), 7.61–7.55 (m, 1H, H-5, CO-pyrrole), 7.48–7.45 (m, 4H, Ph), 7.36–7.27 (m, 7H, Ph), 7.24–7.20 (m, 4H, Ph), 7.05–7.03 (m, 1H, pyrrole), 2.36 (s, 3H, CH_3_). ^13^C NMR (100.6 MHz, CDCl_3_): *δ* 192.6, 144.9, 139.6, 137.4, 137.0, 133.2, 132.7, 129.8 (3C), 129.6, 129.2, 128.8 (2C), 128.7 (2C), 128.5 (2C), 128.4 (2C), 127.4 (2C), 127.1 (2C), 126.8, 126.0, 125.5, 124.4, 123.6, 122.4, 121.8, 120.8, 115.7, 21.7. Anal. Calcd for C_34_H_26_N_2_O_3_S: C, 74.95; H, 4.68; N, 5.13; S, 5.91%. Found: C, 75.45; H, 4.98; N, 5.30; S, 5.79%. HRMS (ESI-TOF): found 543,1742. Calcd. for [C_34_H_26_N_2_O_3_S+H]^+^ 543,1739.

(E)-1-Phenyl-2-(1*H*-pyrrolo[1,2-*c*]imidazol-1-ylidene)-3-tosylpropan-1-one (**3a**). 

Yield 144 mg (37%). Yellow crystals, mp 183–185°C. IR (KBr, cm^−1^): 3063 (=CH), 2923, 1689 (CO), 1596, 1581, 1523, 1493 (C=C), 1320, 1153 (SO_2_). ^1^H NMR (400.13 MHz, CDCl_3_): *δ* 8.64 (s, 1H, H-3, pyrroloimidazole), 8.14–8,12 (m, 2H, Ph), 7.90–7.88 (m, 2H, Ph), 7.66–7.62 (m, 1H, Ph), 7.56–7.53 (m, 2H, Ph), 7.44–7.43 (m, 1H, H-5, pyrroloimidazole), 7.29–7.27 (m, 2H, Ph), 6.94–6.93 (m, 1H, H-6, pyrroloimidazole), 6.67–6.65 (m, 1H, H-7, pyrroloimidazole, 5.17 (s, 2H, CH_2_), 2.39 (s, 3H, CH_3_). ^13^C NMR (100.6 MHz, CDCl_3_): δ 195.2, 144.4, 138.3, 137.6, 136.9, 136.8, 133.7, 132.1, 129.6 (2C), 128.9 (2C), 128.8 (2C), 128.5 (2C), 124.2, 118.3, 114.2, 104.8, 38.1, 21.8. Anal. Calcd for C_22_H_18_N_2_O_3_S: C, 67.68; H, 4.65; N, 7.17; S, 8.21%. Found: C, 67.80; H, 4.83; N, 7.30; S, 8.09%. HRMS (ESI-TOF): found 391,1116. Calcd. for [C_22_H_18_O_3_SN_2_+H]^+^ 391,1120.

(*E*)-1-(Furan-2-yl)-2-(1*H*-pyrrolo[1,2-*c*]imidazol-1-ylidene)-3-tosylpropan-1-one (**3b**). 

Yield 133 mg (35%). Yellow crystals, mp 90–92 °C. IR (KBr, cm^−1^): 3132 (=CH), 2923, 2853 (CH), 1679 (CO), 1621, 1595, 1521, 1467 (C=C), 1320, 1153 (SO_2_). ^1^H NMR (400.13 MHz, CDCl_3_): *δ* 8.63 (s, 1H, H-3, pyrroloimidazole), 7.93–7.91 (m, 2H, Ph), 7.66–7.67 (m, 1H, H-5, furan), 7.43–7.41 (m, 1H, H-3, furan), 7.38–7.37 (m, 1H, H-5, pyrroloimidazole), 7.30–7.28 (m, 2H, Ph), 6.94–6.92 (m, 1H, H-6, pyrroloimidazole), 6.72–6.71 (m, 1H, H-7, pyrroloimidazole), 6.62 (dd, *J* = 3.4, 1.5 Hz, 1H, H-4, furan), 5.05 (s, 2H, CH_2_), 2.40 (s, 3H, CH_3_). ^13^C NMR (100.6 MHz, CDCl_3_): *δ* 184.1, 152.4, 146.8, 144.4, 138.5, 137.6, 136.9, 132.0, 129.6 (2C), 128.9 (2C), 123.2, 118.4, 117.8, 114.2, 112.7, 104.9, 37.6, 21.8. Anal. Calcd for C_20_H_16_N_2_O_4_S: C, 63.30; H, 4.24; N, 7.21; S, 8.43%. Found: C, 63.30; H, 4.39; N, 7.48; S, 8.31%. HRMS (ESI-TOF): found 381,0909. Calcd. for [C_20_H_16_N_2_O_4_S+H]^+^ 381,0911.

(*E*)-2-(6-Ethyl-5-propyl-1*H*-pyrrolo[1,2-*c*]imidazol-1-ylidene)-1-phenyl-3-tosylpropan-1-one (**3c**). 

Yield 147 mg (32%). Yellow crystals, mp 153–155 °C. IR (KBr, cm^−1^): 3129 (=CH), 2963, 2932, 2872 (CH), 1690 (CO), 1597, 1581, 1495 (C=C), 1318, 1129 (SO_2_). ^1^H NMR (400.13 MHz, CDCl_3_): *δ* 8.51 (s, 1H, H-3, pyrroloimidazole), 8.15–8,13 (m, 2H, Ph), 7.90–7.88 (m, 2H, Ph), 7.65–7.61 (m, 1H, Ph), 7.56–7.52 (m, 2H, Ph), 7.28–7.26 (m, 2H, Ph), 6.55–6.53 (m, 1H, H-6, pyrroloimidazole), 5.13 (s, 2H, CH_2_), 2.85–2.81 (m, 2H, CH_2_), 2.62–2.57 (m, 2H, CH_2_), 2.38 (s, 3H, CH_3_), 1.63–1.57 (m, 2H, CH_2_), 1.19 (t, *J* =7.5 Hz, 3H, CH_3_), 0.94 (t, *J* = 7.3 Hz, 3H, CH_3_). ^13^C NMR (100.6 MHz, CDCl_3_): δ 195.5, 144.1, 137.9, 136.9, 136.6, 133.9, 133.8, 133.5, 130.9, 129.5 (2C), 128.9 (2C), 128.7 (2C), 128.5 (2C), 124.3, 123.1, 103.9, 38.2, 25.6, 21.7, 21.6, 19.5, 15.2, 14.0. Anal. Calcd for C_27_H_28_N_2_O_3_S: C, 70.41; H, 6.13; N, 6.08; S, 6.96%. Found: C, 70.14; H, 6.15; N, 6.98; S, 6.77%. HRMS (ESI-TOF): found 461,1899. Calcd. for [C_27_H_28_N_2_O_3_S+H]^+^ 461,1900.

(*E*)-1-Phenyl-2-(5-phenyl-1*H*-pyrrolo[1,2-*c*]imidazol-1-ylidene)-3-tosylpropan-1-one (**3d**). 

Yield 149 mg (32%). Yellow crystals, mp 165–167 °C. IR (KBr, cm^−1^): 3134, 3061 (=CH), 2923, 2853 (CH), 1688 (CO), 1596, 1455 (C=C), 1318, 1145 (SO_2_). ^1^H NMR (400.13 MHz, CDCl_3_): *δ* 8.91 (s, 1H, H-3, pyrroloimidazole), 8.17–8.15 (m, 2H, Ph), 7.90–7.88 (m, 2H, Ph), 7.68–7.64 (m, 1H, Ph), 7.58–7.55 (m, 2H, Ph), 7.49–7.48 (m, 4H, Ph), 7.43–7.39 (m, 1H, Ph), 7.29–7.27 (m, 2H, Ph), 6.98 (d, *J* = 3.7 Hz, 1H, H-6, pyrroloimidazole), 6.76 (d, *J* = 3.7 Hz, 1H, H-7, pyrroloimidazole), 5.20 (s, 2H, CH_2_), 2.39 (s, 3H, CH_3_). ^13^C NMR (100.61 MHz, CDCl_3_): *δ* 195.4, 144.4, 138.3, 137.6, 136.9, 135.1, 133.7, 132.8, 130.1, 129.5 (2C), 129.4 (2C), 129.0 (2C), 128.9 (2C), 128.8, 128.6 (3C), 128.5 (2C), 124.2, 118.2, 105.2, 38.1, 21.8. Anal. Calcd for C_28_H_22_N_2_O_3_S: C, 72.08; H, 4.75; N, 6.00; S, 6.87%. Found: C, 72.24; H, 4.94; N, 6.15; S, 6.74%. HRMS (ESI-TOF): found 467,1429. Calcd. for [C_28_H_22_N_2_O_3_S+H]^+^ 467,1435.

(*E*)-1-(Furan-2-yl)-2-(5-phenyl-1*H*-pyrrolo[1,2-*c*]imidazol-1-ylidene)-3-tosylpropan-1-one (**3e**). 

Yield 160 mg (35%). Yellow crystals, mp 183–185 °C. IR (KBr, cm^−1^): 3133, 3063 (=CH), 2924 (CH), 1680 (CO), 1596, 1589, 1537 (C=C), 1317, 1145 (SO_2_). ^1^H NMR (400.13 MHz, CDCl_3_): *δ* 8.90 (s, 1H, H-3, pyrroloimidazole), 7.95–7.93 (m, 2H, Ph), 7.70–7.69 (m, 1H, H-5, furan), 7.52–7.48 (m, 4H, Ph), 7.42–7.41 (m, 2H, *p*-Ph, H-3, furan), 7.31–7.29 (m, 2H, Ph), 6.99 (d, *J* = 4.1 Hz, 1H, H-6, pyrroloimidazole), 6.82 (d, *J* = 4.1 Hz, 1H, H-7, pyrroloimidazole), 6.64 (dd, *J* = 3.6, 1.7 Hz, 1H, H-4, furan), 5.09 (s, 2H, CH_2_), 2.40 (s, 3H, CH_3_). ^13^C NMR (100.6 MHz, CDCl_3_): *δ* 184.0, 152.2, 146.6, 144.2, 138.3, 137.4, 134.9, 132.5, 129.8, 129.3 (2C), 129.2 (2C), 128.8 (2C), 128.7, 128.6, 128.4 (2C), 123.0, 118.0, 117.6, 112.5, 105.1, 37.5, 21.6. Anal. Calcd for C_26_H_20_N_2_O_4_S: C, 68.04; H, 4.42; N, 6.14; S, 7.02%. Found: C, 68.54; H, 4.56; N, 6.27; S, 6.88%. HRMS (ESI-TOF): found 457,1222. Calcd. for [C_26_H_20_N_2_O_4_S+H]^+^ 457,1229.

(*E*)-2-(5-Phenyl-1*H*-pyrrolo[1,2-*c*]imidazol-1-ylidene)-1-(thiophen-2-yl)-3-tosylpropan-1-one (**3f**). 

Yield 175 mg (37%). Yellow crystals, mp 155–157 °C. IR (KBr, cm^−1^): 3120, 3097 (=CH), 2861 (CH), 1663 (CO), 1595, 1518, 1494 (C=C), 1318, 1122 (SO_2_). ^1^H NMR (400.13 MHz, CDCl_3_): *δ* 8.87 (s, 1H, H-3, pyrroloimidazole), 7.99–7.98 (m, 1H, H-5, thiophene), 7.90–7.87 (m, 2H, Ph), 7.70–7.68 (m, 1H, H-3, thiophene), 7.45–7.40 (m, 4H, Ph), 7.39–7.34 (m, 1H, Ph), 7.27–7.25 (m, 2H, Ph), 7.21-.7.18 (m, 1H, H-3, thiophene), 6.94–6.93 (m, 1H, H-6, pyrroloimidazole), 6.78–6.77 (m, 1H, H-7, pyrroloimidazole), 5.12 (s, 2H, CH_2_), 2.35 (s, 3H, CH_3_). ^13^C NMR (100.6 MHz, CDCl_3_): δ 187.9, 144.4, 143.5, 138.3, 137.5, 135.1, 134.2, 132.8, 132.6, 129.9, 129.5 (2C), 129.4 (2C), 128.9, 128.8 (2C), 128.6, 128.5 (2C), 128.4, 123.4, 118.2, 105.4, 38.4, 21.7. Anal. Calcd for C_26_H_20_N_2_O_3_S_2_: C, 66.08; H, 4.27; N, 5.93; S, 13.57%. Found: C, 65.91; H, 4.05; N, 6.11; S, 13.39%. HRMS (ESI-TOF): found 473,0994. Calcd. for [C_26_H_20_N_2_O_3_S_2_+H]^+^ 473,0995.

(*E*)-2-(5-(4-(Dimethylamino)phenyl)-1*H*-pyrrolo[1,2-*c*]imidazol-1-ylidene)-1-phenyl-3-tosylpropan-1-one (**3g**). 

Yield 143 mg (28%). Yellow crystals, mp 108–110 °C. IR (KBr, cm^−1^): 3018 (=CH), 2924 (CH), 1688 (CO), 1612, 1597 (C=C), 1318, 1145 (SO_2_). ^1^H NMR (400.13 MHz, CDCl_3_): *δ* 8.89 (s, 1H, H-3, pyrroloimidazole), 8.17–8.15 (m, 2H, Ph), 7.90–7.88 (m, 2H, Ph), 7.67–7.62 (m, 1H, Ph), 7.57–7.54 (m, 2H, Ph), 7.35–7.33 (m, 2H, Ph), 7.28–7.26 (m, 2H, Ph), 6.87 (d, *J* = 3.9 Hz, 1H, H-6, pyrroloimidazole), 6.79–6.77 (m, 2H, Ph), 6.73 (d, *J* = 3.9 Hz, 1H, H-7, pyrroloimidazole), 5.18 (s, 2H, CH_2_), 3.02 (s, 6H, N(CH_3_)_2_), 2.39 (s, 3H, CH_3_). ^13^C NMR (100.6 MHz, CDCl_3_): δ 195.5, 150.6, 144.2, 137.8, 137.4, 136.9, 135.3, 133.6, 132.0, 130.5, 129.8, 129.6 (3C), 129.5 (2C), 128.9 (2C), 128.8 (2C), 128.5 (3C), 124.2, 117.3, 112.6, 105.1, 38.1 (2C), 21.8. Anal. Calcd for C_30_H_27_N_3_O_3_S: C, 70.71; H, 5.34; N, 8.25; S, 6.29%. Found: C, 70.85; H, 5.53; N, 8.39; S, 6.12%. HRMS (ESI-TOF): found 510,1851. Calcd. for [C_30_H_27_N_3_O_3_S+H]^+^ 510,1856.

(*E*)-2-(5-(4-Fluorophenyl)-1*H*-pyrrolo[1,2-*c*]imidazol-1-ylidene)-1-phenyl-3-tosylpropan-1-one (**3h**).

Yield 184 mg (38%). Yellow crystals, mp 95–97 °C. IR (KBr, cm^−1^): 3131, 3063 (=CH), 2923 (CH), 1689 (CO), 1596, 1539, 1510, 1493 (C=C), 1316, 1146 (SO_2_). ^1^H NMR (400.13 MHz, CDCl_3_): *δ* 8.81 (s, 1H, H-3, pyrroloimidazole), 8.16–8,14 (m, 2H, Ph), 7.90–7.88 (m, 2H, Ph), 7.67–7.63 (m, 1H, Ph), 7.58–7.54 (m, 2H, Ph), 7.47–7.44 (m, 2H, Ph), 7.29–7.27 (m, 2H, Ph), 7.20–7.16 (m, 2H, Ph), 6.93 (d, *J* = 4.0 Hz, 1H, H-6, pyrroloimidazole), 6.75 (d, *J* = 4.0 Hz, 1H, H-7, pyrroloimidazole), 5.20 (s, 2H, CH_2_), 2.39 (s, 3H, CH_3_). ^13^C NMR (100.6 MHz, CDCl3): δ 195.3, 162.9 (d, ^1^*J*_CF_ = 250.5 Hz, C4, 4F-C_6_H_4_), 144.4, 138.3, 137.5, 136.8, 134.8, 133.7, 132.7, 130.6 (d, ^3^*J*_CF_ = 8.3 Hz, C-2,6 4F-C_6_H_4_, 2C), 129.5 (2C), 129.0 (2C), 128.9 (2C), 128.5 (2C), 127.4, 126.1 (d, ^4^*J*_CF_ = 3.3 Hz, C-1 4F-C_6_H_4_), 124.2, 118.2, 116.6 (d, ^2^*J*_CF_ = 21.8 Hz, C-3,5 4F-C_6_H_4_, 2C), 105.1, 38.1, 21.8. Anal. Calcd for C_28_H_21_FN_2_O_3_S: C, 69.41; H, 4.37; F, 3.92; N, 5.78; S, 6.62%. Found: C, 69.52; H, 4.51; N, 5.97; S, 6.47; F, 4.04%. HRMS (ESI-TOF): found 485,1335. Calcd. for [C_28_H_21_FN_2_O_3_S+H]^+^ 485,1339. 

(*E*)-2-(5-(4-Fluorophenyl)-1*H*-pyrrolo[1,2-*c*]imidazol-1-ylidene)-1-(furan-2-yl)-3-tosylpropan-1-one (**3i**).

Yield 157 mg (33%). Yellow crystals, mp 188–190 °C. IR (KBr, cm^−1^): 3131, 3056 (=CH), 2925 (CH), 1680 (CO), 1602, 1561, 1570, 1540, 1513 (C=C), 1317, 1161 (SO_2_). ^1^H NMR (400.13 MHz, CDCl_3_): *δ* 8.80 (s, 1H, H-3, pyrroloimidazole), 7.93–7,91 (m, 2H, Ph), 7.68–7.67 (m, 1H, H-5, furan), 7.46–7.43 (m, 2H, H-3, furan), 7.41–7.40 (m, 1H, Ph), 7.30–7.28 (m, 2H, Ph), 7.19–7.15 (m, 2H, Ph), 6.93 (d, *J* = 4.0 Hz, 1H, H-6, pyrroloimidazole), 6.79 (d, *J* = 4.0 Hz, 1H, H-7, pyrroloimidazole), 6.63 (dd, *J* = 2.5, 1.4 Hz, 1H, H-4, furan), 5.08 (s, 2H, CH_2_), 2.39 (s, 3H, CH_3_). ^13^C NMR (100.6 MHz, CDCl_3_): *δ* 184.1, 162.9 (d, ^1^*J*_CF_ = 249.8 Hz, C4, 4F-C_6_H_4_), 152.3, 146.8, 144.4, 138.5, 137.5, 134.9, 132.6, 130.5 (d, ^3^*J*_CF_ = 8.3 Hz, C-2,6 4F-C_6_H_4_, 2C), 129.5 (2C), 128.9 (2C), 127.4, 126.1 (d, ^4^*J*_CF_ = 3.4 Hz, C-1 4F-C_6_H_4_), 123.2, 118.2, 117, 116.6 (d, ^2^*J*_CF_ = 21.8 Hz, C-3,5 4F-C_6_H_4_, 2C), 112.7, 105.3, 37.6, 21.7. Anal. Calcd for C_26_H_19_FN_2_O_4_S: C, 65.81; H, 4.04; F, 4.00; N, 5.90; S, 6.76%. Found: C, 65.99; H, 4.19; N, 6.10; S, 6.60; F, 4.12%. HRMS (ESI-TOF): found 475,1128. Calcd. for [C_26_H_19_FN_2_O_4_S+H]^+^ 475,1128.

(*E*)-2-(5-(4-chlorophenyl)-1*H*-pyrrolo[1,2-*c*]imidazol-1-ylidene)-1-phenyl-3-tosylpropan-1-one (**3j**). 

Yield 160 mg (32%). Yellow crystals, mp 135–137 °C. IR (KBr, cm^−1^): 3056 (=CH), 2924 (CH), 1688 (CO), 1596, 1581, 1532, 1494 (C=C), 1315, 1145 (SO_2_). ^1^H NMR (400.13 MHz, CDCl_3_): *δ* 8.84 (s, 1H, H-3, pyrroloimidazole), 8.16–8,14 (m, 2H, Ph), 7.90–7.88 (m, 2H, Ph), 7.67–7.64 (m, 1H, Ph), 7.58–7.54 (m, 2H, Ph), 7.47–7.41 (m, 4H, Ph), 7.29–7.27 (m, 2H, Ph), 6.96 (d, *J* = 4.1 Hz, 1H, H-6, pyrroloimidazole), 6.76 (d, *J* = 4.1 Hz, 1H, H-7, pyrroloimidazole), 5.20 (s, 2H, CH_2_), 2.39 (s, 3H, CH_3_). ^13^C NMR (100.6 MHz, CDCl_3_): δ 194.7, 143.8, 137.9, 136.8, 136.1, 134.3, 134.2, 133.1, 132.4, 129.1 (2C), 129.0 (2C), 128.9 (2C), 128.3 (2C), 128.2 (2C), 127.9 (2C), 127.8, 126.6, 123.6, 117.7, 104.6, 37.4, 21.1. Anal. Calcd for C_28_H_21_ClN_2_O_3_S: C, 67.13; H, 4.23; Cl, 7.08; N, 5.59; S, 6.40%. Found: C, 67.25; H, 4.39; N, 5.78; S, 6.25%.

## 4. Conclusions

In summary, the base-catalyzed cycloaddition of readily available acylethynylpyrroles with TosMIC was studied to develop a feasible one-pot syntheses, which is pharmaceutically prospective, but still rare functionalized 2,3′-bipyrroles and previously unknown pyrrolo[1,2-*c*]imidazoles have exocyclic double bonds with tosyl and acyl substituents. The elaborated methods are easy to operate and demonstrate good substrate coverage. The synthesized compounds represent a promising rewarding platform for drug design and materials science. 

## Figures and Tables

**Table 1:** Effects of reaction conditions on the synthesis of 2,3′-bipyrroles **2a** and pyrroloimidazole **3a** from 2-benzoylethynylpyrrole **1a** and TosMIC.

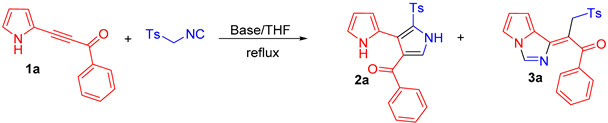					
Entry ^a^	Base	Time, h	Conversion of 1a, %	Yield of 2a, % ^b^	Yield of 3a, % ^b^
1	NEt_3_	24	0	0	0
2	DBU	24	0	0	0
3	DABCO	24	0	0	0
4	K_2_CO_3_	24	45	15	10
5	Cs_2_CO_3_	1	100	56	0
6	NaOH	1	100	72	traces
7	KOH	1	100	80	traces
8 ^c^	*t*-BuONa	1	100	15	23
9	*t*-BuONa	1	100	27	37
10 ^c^	*t*-BuOK	1	75	43	traces
11	*t*-BuOK	1	100	80	traces
12 ^d^	NaH	1	90	17	31
13 ^c^	NaH	1	90	traces	39
14	NaH	1	100	traces	45
15 ^e^	NaH	1	100	traces	42

^a^ The reactions were carried out at a molar ratio of **1a**:TosMIC:base = 1:2:2, 20 mL THF; ^b^ Isolated yield; ^c^ The reactions were carried out at a molar ratio of **1a**:TosMIC:base = 1:1:1; ^d^ The reactions were carried out at a molar ratio of **1a**:TosMIC:base = 1:1:0.5; ^e^ 1 eq. t-BuOH was added.

## Data Availability

The data presented in this study are available in the Supplementary Materials.

## Supplementary Materials

The following supporting information can be downloaded at: https://www.mdpi.com/article/10.3390/molecules29040885/s1. File S1: The synthesis of detailed procedures and physicochemical characterization of products: 2,3′-bipyrroles (**2a**–**o**) and pyrrolo[1,2-*c*]imidazoles (**3a**–**j**); ^1^H NMR and ^13^C NMR spectra of compounds **2a**–**o** and **3a**–**j**.
