# Supplementary material for: Functionalized 2,3′-Bipyrroles and Pyrrolo[1,2-c]imidazoles from Acylethynylpyrroles and Tosylmethylisocyanide
Source: Molecules. 2024 Feb 17;29(4):885. doi: 10.3390/molecules29040885 (PMC10893325; doi:10.3390/molecules29040885)

Supporting Information

Maxim D. Gotsko, Ivan V. Saliy, Igor A. Ushakov, Lyubov' N. Sobenina, Boris A. Trofimov

A.E. Favorsky Irkutsk Institute of Chemistry, Siberian Branch of the Russian Academy of Sciences, 1 Favorsky St., Irkutsk 664033, Russian Federation

Corresponding author: Tel.: 7 (3952) 51-14-31; fax 7 (3952) 41-93-46; e-mail: boris_trofimov@irioch.irk.ru

Table of contents

1. General information 2
2. Starting materials 2
3. The reaction of acylethynylpyrroles 1 with TosMIC 2
4. Characterization data of 2,3-bipyrroles 2a-o and pyrrolo[1,2-*c*]imidazoles 3a-j 3
5. The NMR spectra of 2,3-bipyrroles (2a-o) and pyrrolo[1,2-c]imidazoles (3a-j) and HRMS spectra 13

1. General information

IR spectra were obtained with a Bruker Vertex 70 spectrometer (400–4000 cm^{-1}, films). ^1H (400.13 MHz), ^{13}C (100.6 MHz) spectra were recorded on a Bruker DPX-400 spectrometer at ambient temperature in $CDCl_3$ and DMSO-d_6 solutions and referenced to $CDCl_3$ (residual protons of $CDCl_3$ in ^1H NMR δ = 7.26 ppm; ^{13}C NMR δ = 77.1 ppm), DMSO-d_6 (residual protons of $CDCl_3$ in ^1H NMR δ = 2.50 ppm; ^{13}C NMR δ = 39.52 ppm).

The C, H, N microanalyses were performed on a Flash EA 1112 CHNS-O/MAS analyzer. Sulfur was determined by complexometric titration with Chlorasenazo III. Fluorine content was determined on a SPECOL 11 (Carl Zeiss Jena, Germany) spectrophotometer. Clorine was determined by mercurimetric titration. Melting point (uncorrected) was determined on a Kofler micro hot-stage apparatus. High-resolution mass spectral analyses were performed from acetonitrile solution with 0.1% HFBA on HPLC Agilent 1200/Agilent 6210 TOF instrument equipped with an electrospray ionization (ESI) source (Agilent, USA).

All reactions were carried out in dried glassware in the presence of air.

2. Starting materials

Acylethynylpyrroles **1a-o** were prepared from corresponding pyrroles and acylbromoacetylenes in the presence of Al_2O_3 according to the reported procedure[1], tosylmethylisocyanide (TosMIC), *t*-BuONa, KOH, NaH and THF are commercial products.

3. The reaction of acylethynylpyrroles 1 with TosMIC

3.1. The reaction of acylethynylpyrroles 1a-o with TosMIC in the presence of KOH. Synthesis of 2,3'-bipyrroles 2a-o (General Procedure)

A solution of acylethynylpyrrole **1a-o** (1 mmol) and TosMIC (395 mg, 2 mmol) in THF (10 mL) was heated to reflux. Then, under reflux, suspension of KOH·0.5H_2O (130 mg, 2 mmol) in THF (10 mL) was added dropwise to reaction mixture for 1 hour. The residue, after removing solvent, was fractionated by column chromatography (SiO_2, *n*-hexane : diethyl ether, 5 : 1) to afford the 2,3'-bipyrrole **2a-o**.

3.2. The reaction of acylethynylpyrroles 1a-d,i-n with TosMIC in the presence of t-BuONa. Synthesis of 2,3'-bipyrroles 2a-b, d, h-n and pyrrolo[1,2-c]imidazoles 3a-j (General Procedure)

A solution of acylethynylpyrrole **1a-b, d, h-n** (1 mmol) and TosMIC (395 mg, 2 mmol) in THF (10 mL) was heated to reflux. Then, under reflux, solution of *t*-BuONa (224 mg, 2 mmol) in THF (10 mL) was added dropwise to reaction mixture for 1 hour. The residue, after removing solvent, was fractionated by column chromatography (SiO_2, *n*-hexane : diethyl ether, 5 : 1) to afford the 2,3'-bipyrrole **2a-b, d, h-n** then pyrrolo[1,2-*c*]imidazoles **3a-j**. Yields of 2,3'-bipyrrole **2a-b, d, h-n**, thus obtained, are: **2a** - 27%; **2b** - 32%; **2d** - 32%; **2h** - 24%; **2i** - 29%, **2j** - 24%; **2k** - 30%; **2l** - 22%; **2m** - 26%; **2n** - 24%.

4. Characterization data of 2,3-bipyrroles 2a-o and pyrrolo[1,2-*c*]imidazoles 3a-j
4.1. Characterization data of 2,3-bipyrroles 2a-o

*Phenyl(2'-tosyl-1H,1'H-[2,3'-bipyrrol]-4'-yl)methanone (**2a**)*

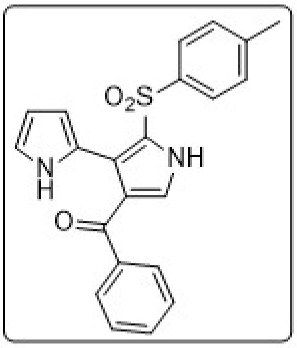

Yield 312 mg (80%). Yellow crystals, mp 163-165 °C. IR (KBr, cm^{-1}): 3295 (NH), 3148, 3063 (=CH), 2920 (CH), 1636 (CO), 1597, 1517 (C=C), 1354, 1140 (SO_2). ^1H NMR (400.13 MHz, $CDCl_3$): δ 10.89 (br.s, 1H, NH, CO-pyrrole), 9.92 (br.s, 1H, NH, pyrrole), 7.75-7.73 (m, 2H, Ph), 7.59-7.57 (m, 2H, Ph), 7.54-7.52 (m, 1H, Ph), 7.44-7.40 (m, 2H, Ph), 7.24 (d, J = 3.5 Hz, 1H, H-5, CO-pyrrole), 7.16-7.14 (m, 2H, Ph), 6.84-6.83 (m, 2H, H-5, H-3, pyrrole), 6.20-6.18 (m, 1H, H-4, pyrrole), 2.34 (s, 3H, CH_3). ^{13}C NMR (100.6 MHz, $CDCl_3$): δ 192.4, 144.7, 139.3, 137.5, 132.7, 129.8 (2C), 129.7 (2C), 129.3, 128.4 (2C), 127.2 (2C), 125.2, 124.3, 123.0, 121.4, 119.2, 113.1, 109.5, 21.7. Anal. Calcd for $C_{22}H_{18}N_2O_3S$: C, 67.68; H, 4.65; N, 7.17; S, 8.21%. Found: C, 67.84; H, 4.78; N, 7.32; S, 8.01%. HRMS (ESI-TOF): found 391,1116. Calcd. for $[C_{22}H_{18}N_2O_4S+H]^+$ 391,1121.

*Furan-2-yl(2'-tosyl-1H,1'H-[2,3'-bipyrrol]-4'-yl)methanone (**2b**)*

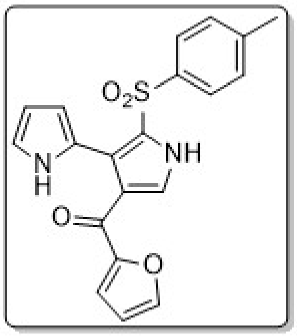

Yield 282 mg (74%). Yellow crystals, mp 95-97 °C. IR (KBr, cm^{-1}): 3293 (NH), 3131 (=CH), 2923, 2859 (CH), 1620 (CO), 1563, 1520 (C=C), 1354, 1140 (SO_2). ^1H NMR (400.13 MHz, $CDCl_3$): δ 10.71 (br.s, 1H, NH, CO-pyrrole), 9.94 (br.s, 1H, NH, pyrrole), 7.68 (d, J = 3.3 Hz, 1H, H-5, CO-pyrrole), 7.62-7.61 (m, 1H, H-5, furan), 7.57-7.55 (m, 2H, Ph), 7.16-7.14 (m, 3H, H-3, furan, Ph), 6.86-6.84 (m, 2H, H-3, H-5, pyrrole), 6.54 (dd, J = 3.3, 1.4 Hz, 1H, H-4, furan), 6.22-6.20 (m, 1H, H-4, pyrrole), 2.33 (s, 3H, CH_3). ^{13}C NMR (100.6 MHz, $CDCl_3$): δ 177.8, 153.4, 146.9, 144.7, 137.9, 129.7 (2C), 127.5, 127.3 (2C), 125.7, 123.3, 123.0, 121.4, 119.8, 119.3, 113.2, 112.5, 109.5, 21.6. Anal. Calcd for $C_{20}H_{16}N_2O_4S$: C, 63.15; H, 4.24; N, 7.36; S, 8.43%. Found: C, 63.27; H, 4.42; N, 7.53; S, 8.3%.

*Thiophen-2-yl(2'-tosyl-1H,1'H-[2,3'-bipyrrol]-4'-yl)methanone (**2c**)*

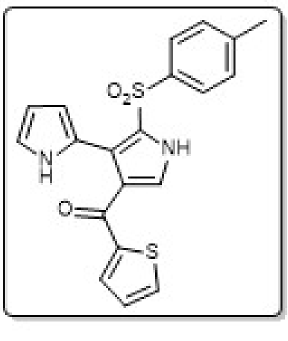

Yield 274 mg (69%). Yellow crystals, mp 175-177 °C. IR (KBr, cm^{-1}): 3351 (NH), 3137, 3110 (=CH), 2922 (CH), 1614 (CO), 1569, 1520, 1484, 1458 (C=C), 1359, 1136 (SO_2). ^1H NMR (400.13 MHz, $CDCl_3$): δ 10.64 (br.s, 1H, NH, CO-pyrrole), 10.16 (br.s, 1H, NH, pyrrole), 7.65 (d, J = 4.9 Hz, 1H, H-5, thiophene), 7.57 (d, J = 8.1 Hz, 2H, Ph), 7.54 (d, J = 3.5 Hz, 1H, H-3, thiophene), 7.46 (d, J =

3.3 Hz, 1H, H-3, pyrrole), 7.14 (d, J = 8.1 Hz, 2H, Ph), 7.09-7.07 (m, 1H, H-4, thiophene), 6.85-6.83 (m, 1H, H-4, pyrrole), 6.83-6.82 (m, 1H, H-3, pyrrole), 6.21-6.20 (m, 1H, H-5, pyrrole), 2.32 (s, 3H, CH_3). ^{13}C NMR (100.6 MHz, $CDCl_3$): δ 183.5, 145.0, 144.8, 137.5, 134.6, 134.4, 129.7 (2C), 128.2, 127.4, 127.2 (2C), 125.4, 124.1, 122.5, 121.4, 119.3, 113.0, 109.4, 21.7. Anal. Calcd for $C_{20}H_{16}N_2O_3S_2$: C, 60.59; H, 4.07; N, 7.07; S, 16.17%. Found: C, 60.73; H, 4.27; N, 7.18; S, 15.99%. HRMS (ESI-TOF): found 397,0681. Calcd. for $[C_{20}H_{16}N_2O_3S_2+H]^+$ 397,0685.

*(4-Ethyl-5-propyl-2'-tosyl-1H,1'H-[2,3'-bipyrrol]-4'-yl)(phenyl)methanone (**2d**)*

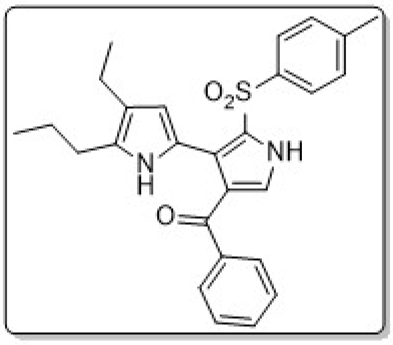

Yield 203 mg (44%). Yellow crystals, mp 170-172 °C. IR (KBr, cm^{-1}): 3285 (NH), 3160 (=CH), 2958, 2927, 2869 (CH), 1630 (CO), 1596, 1575, 1519, 1495, 1461 (C=C), 1351, 1142, (SO_2). ^1H NMR (400.13 MHz, $CDCl_3$): δ 10.63 (br.s, 1H, NH, CO-pyrrole), 9.84 (br.s, 1H, NH, pyrrole), 7.72-7.70 (m, 2H, Ph), 7.63-7.61 (m, 2H, Ph), 7.54-7.51 (m, 1H, Ph), 7.43-7.39 (m, 2H, Ph), 7.22 (d, J = 3.5 Hz, 1H, H-5, CO-pyrrole), 7.16-7.14 (m, 2H, Ph), 6.67-6.65 (m, 1H, pyrrole), 2.55-2.53 (m, 2H, CH_2), 2.51-2.53 (m, 2H, CH_2), 2.37 (s, 3H, CH_3), 1.67-1.57 (m, 2H CH_2), 1.12 (t, J = 7.6 Hz, 3H, CH_3), 0.90 (t, J = 7.3 Hz, 3H, CH_3). ^{13}C NMR (100.6 MHz, $CDCl_3$): δ 192.5, 144.5, 139.8, 137.7, 132.4, 130.1, 129.7 (2C), 129.6 (2C), 129.5, 128.4 (2C), 127.3 (2C), 124.1, 124.0, 123.8, 123.2, 118.7, 113.5, 28.0, 23.1, 21.7, 19.0, 16.1, 13.9. Anal. Calcd for $C_{27}H_{28}N_2O_3S$: C, 70.41; H, 6.13; N, 6.08; S, 6.96%. Found: C, 70.6; H, 6.28; N, 6.25; S, 6.84%. HRMS (ESI-TOF): found 461,1899. Calcd. for $[C_{27}H_{28}N_2O_3S +H]^+$ 461,1902.

*Phenyl(4-(4,5,6,7-tetrahydro-1H-indol-2-yl)-5-tosyl-1H-pyrrol-3-yl)methanone (**2e**)*

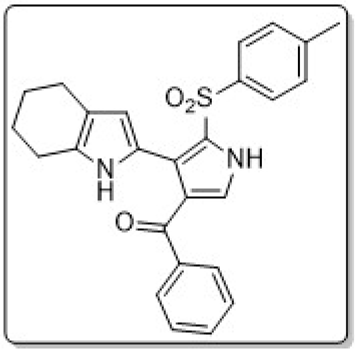

Yield 311 mg (70%). Yellow crystals, mp 235-237 °C. IR (KBr, cm^{-1}): 3342 (NH), 3171 (=CH), 2922, 2851 (CH), 1633 (CO), 1594, 1574, 1521, 1495 (C=C), 1351, 1126 (SO_2). ^1H NMR (400.13 MHz, $CDCl_3$): δ 10.60 (br.s, 1H, NH, CO-pyrrole), 9.89 (br.s, 1H, NH, pyrrole), 7.73-7.71 (m, 2H, Ph), 7.65-7.63 (m, 2H, Ph), 7.56-7.52 (m, 1H, Ph), 7.44-7.40 (m, 2H, Ph), 7.20-7.19 (m, 2H, Ph), 7.17-7.15 (m, 1H, H-2, CO-pyrrole), 6.68-6.67 (m, 1H, H-3, pyrrole), 2.61-2.59 (m, 2H, CH_2-7), 2.51-2.48 (m, 2H, CH_2-4), 2.36 (s, 3H, CH_3), 1.79-1.78 (m, 2H, CH_2-5), 1.73-1.71(m, 2H, CH_2-6). ^{13}C NMR (100.61 MHz, $CDCl_3$): δ 192.5, 144.5, 139.8, 137.7, 132.5, 129.7 (3C), 129.6 (2C), 129.2, 128.4 (2C), 127.2 (2C), 124.1, 123.9, 121.3, 119.5, 118.8, 112.4, 24.0, 23.6, 23.1, 23.1, 21.7. Anal. Calcd for $C_{26}H_{24}N_2O_3S$: C, 70.25; H, 5.44; N, 6.30; S, 7.21%. Found: C, 70.40; H, 5.25; N, 6.44; S, 7.04%. HRMS (ESI-TOF): found 445,1586. Calcd. for $[C_{26}H_{24}N_2O_3S+H]^+$ 445,1589.

*Furan-2-yl(4-(4,5,6,7-tetrahydro-1H-indol-2-yl)-5-tosyl-1H-pyrrol-3-yl)methanone (**2f**)*

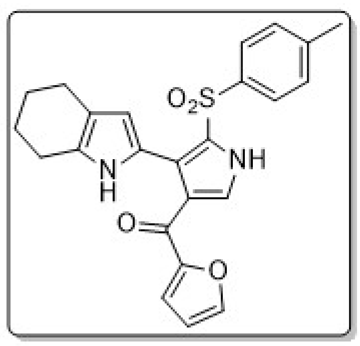

Yield 291 mg (67%). Yellow crystals, mp 173-175 °C. IR (KBr, cm^{-1}): 3278 (NH), 3019 (=CH), 2924, 2850 (CH), 1618 (CO), 1609, 1524, 1464 (C=C), 1351, 1138 (SO_2). ^1H NMR (400.13 MHz, $CDCl_3$): δ 10.39 (br.s, 1H, NH, CO-pyrrole), 10.27 (br.s, 1H, NH, pyrrole), 7.65-7.64 (m, 2H, Ph), 7.62-7.61 (m, 2H, H-5, H-3, furan), 7.16-7.14 (m, 2H, Ph), 7.12 (d, J = 3.5 Hz, 1H, H-2, CO-pyrrole), 6.64 (d, J = 2.2 Hz, 1H, H-3, pyrrole), 6.52 (dd, J = 3.5, 1.6 Hz, 1H, H-4, furan), 2.60-2.57 (m, 2H, CH_2-7), 2.51-2.48 (m, 2H, CH_2-4), 2.33 (s, 3H, CH_3), 1.81-1.75 (m, 2H, CH_2-5), 1.74-1.70 (m, 2H, CH_2-6). ^{13}C NMR (100.6 MHz, $CDCl_3$): δ 177.9, 153.2, 147.0, 144.5, 137.7, 129.6 (2C), 129.1, 128.5, 127.2 (2C), 124.0, 123.8, 122.4, 119.9, 119.3, 118.6, 112.4, 112.3, 24.0, 23.5, 23.1, 23.0, 21.7. Anal. Calcd for $C_{24}H_{22}N_2O_4S$: C, 66.34; H, 5.10; N, 6.45; S, 7.38%. Found: C, 66.41; H, 5.87; N, 6.18; S, 7.22%. HRMS (ESI-TOF): found 435,1379. Calcd. for $[C_{24}H_{22}N_2O_4S+H]^+$ 435,1383.

*(4-(4,5,6,7-Tetrahydro-1H-indol-2-yl)-5-tosyl-1H-pyrrol-3-yl)(thiophen-2-yl)methanone (**2g**)*

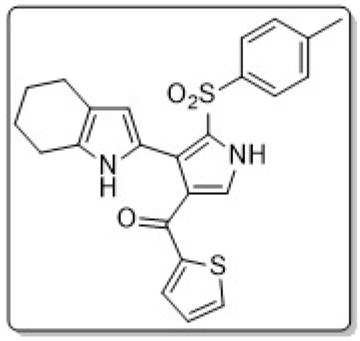

Yield 293 mg (65%). Yellow crystals, mp 198-200 °C. IR (KBr, cm^{-1}): 3289 (NH), 3131 (=CH), 2924, 2851 (CH), 1613 (CO), 1595, 1514 (C=C), 1358, 1140 (SO_2). ^1H NMR (400.13 MHz, $CDCl_3$): δ 10.23 (br.s, 1H, NH, CO-pyrrole), 10.02 (br.s, 1H, NH, pyrrole), 7.66-7.62 (m, 3H, Ph, H-5, thiophene), 7.56-7.55 (m, 1H, H-3, thiophene), 7.43 (d, J = 3.4 Hz, 1H, H-2, CO-pyrrole), 7.17-7.15 (m, 2H, Ph), 7.10-7.08 (m, 1H, H-4 thiophene), 6.63-6.60 (m, 1H, H-3, pyrrole), 2.60-2.57 (m, 2H, CH_2-7), 2.51-2.48 (m, 2H, CH_2-4), 2.35 (s, 3H, CH_3), 1.84-1.76 (m, 2H, CH_2-5), 1.74-1.69(m, 2H, CH_2-6). ^{13}C NMR (100.61 MHz, $CDCl_3$): δ 183.5, 145.3, 144.5, 137.7, 134.3, 134.1, 129.6 (2C), 129.2, 128.1, 127.8, 127.3 (2C), 124.3, 123.7, 123.4, 119.4, 118.6, 112.3, 24.0, 23.6, 23.1, 23.0, 21.7. Anal. Calcd for $C_{24}H_{22}N_2O_3S_2$: C, 63.98; H, 4.92; N, 6.22; S, 14.23%. Found: C, 63.75; H, 5.07; N, 6.08; S, 14.12%. HRMS (ESI-TOF): found 451,1150. Calcd. for $[C_{24}H_{22}N_2O_3S_2+H]^+$ 451,1154.

*Phenyl(5-phenyl-2'-tosyl-1H,1'H-[2,3'-bipyrrol]-4'-yl)methanone (**2h**)*

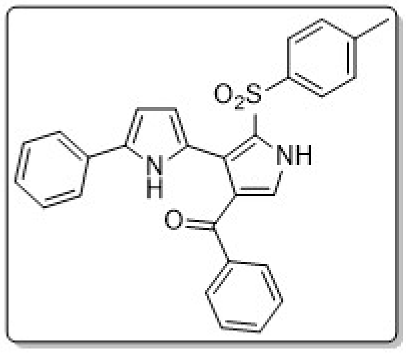

Yield 247 mg (53%). Yellow crystals, mp 205-207 °C. IR (KBr, cm^{-1}): 3281 (NH), 3147 (=CH), 2923 (CH), 1629 (CO), 1597, 1677, 1508, 1477 (C=C), 1349, 1139 (SO2). ^1H NMR (400.13 MHz, $CDCl_3$): δ 11.77 (br.s, 1H, NH, CO-pyrrole), 9.89 (br.s, 1H, NH, pyrrole), 7.78-7.76 (m, 2H, Ph), 7.68-7.66 (m, 2H, Ph), 7.65-7.63 (m, 2H, Ph), 7.58-7.54 (m, 1H, Ph), 7.46-7.43 (m, 2H, Ph), 7.40-7.36 (m, 2H, Ph), 7.28 (d, J = 3.2 Hz, 1H, H-5, CO-pyrrole), 7.21-7.19 (m, 1H, Ph), 7.18-7.16 (m, 2H, Ph), 7.04-7.02 (m, 1H, H-4, pyrrole), 6.56-6.55 (m, 1H, H-3, pyrrole), 2.32 (s, 3H, CH_3). ^{13}C NMR (100.6 MHz, $CDCl_3$): δ 192.7, 144.9, 139.6, 137.4, 133.1, 132.7, 132.6, 129.9 (2C), 129.8 (2C), 129.0 (2C), 128.5 (2C), 127.2 (2C),

126.4, 125.1, 124.2, 123.9 (2C), 122.8, 122.8, 122.7, 120.2, 114.9, 107.4, 21.7. Anal. Calcd for $C_{28}H_{22}N_2O_3S$: C, 72.08; H, 4.75; N, 6.00; S, 6.87%. Found: C, 71.81; H, 4.85; N, 5.89; S, 6.75%. HRMS (ESI-TOF): found 467,1429. Calcd. for $[C_{28}H_{22}N_2O_3S+H]^+$ 467,1431.

*Furan-2-yl(5-phenyl-2'-tosyl-1H,1'H-[2,3'-bipyrrol]-4'-yl)methanone (**2i**)*

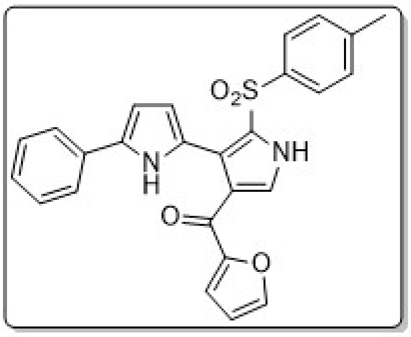

Yield 215 mg (47%). Yellow crystals, mp 90-92 °C. IR (KBr, cm^{-1}): 3278 (NH), 3150 (=CH), 2923 (CH), 1619 (CO), 1519, 1561, 1531, 1509 (C=C), 1351, 1140 (SO_2). ^{1}H NMR (400.13 MHz, $CDCl_3$): δ 11.58 (br.s, 1H, NH of CO-pyrrole), 7.70-7.66 (m, 2H, H-5, furan, H-5, CO-pyrrole), 7.64-7.62 (m, 4H, Ph), 7.39-7.35 (m, 2H, Ph), 7.21-7.17 (m, 1H, Ph), 7.15-7.12 (m, 3H, H-3, furan, Ph), 7.05-7.03 (m, 1H, H-3, pyrrole), 6.57-5.55 (m, 1H, H-4, pyrrole), 6.52 (dd, J = 3.4, 1.5 Hz, 1H, H-4, furan), 2.29 (s, 3H, CH_3). ^{13}C NMR (100.6 MHz, $CDCl_3$): δ 178.0, 153.0, 147.2, 144.8, 137.4, 132.9, 132.5, 129.8 (2C), 129.1, 129.0 (2C), 127.1 (2C), 126.3, 125.1, 123.8 (2C), 122.8 (2C), 122.6, 120.4, 114.7, 112.5, 107.3, 21.6. Anal. Calcd for $C_{26}H_{20}N_2O_4S$: C, 68.41; H, 4.42; N, 6.14; S, 7.02%. Found: C, 68.11; H, 4.29; N, 6.22; S, 6.91%. HRMS (ESI-TOF): found 457,1222. Calcd. for $[C_{26}H_{20}N_2O_4S+H]^+$ 457,1220.

*(5-Phenyl-2'-tosyl-1H,1'H-[2,3'-bipyrrol]-4'-yl)(thiophen-2-yl)methanone (**2j**)*

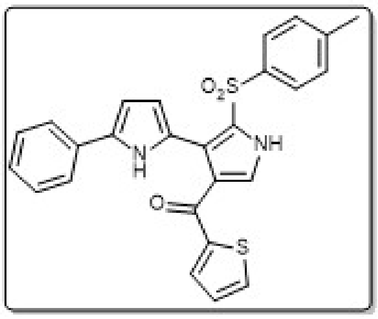

Yield 198 mg (42%). Yellow crystals, mp 105-107 °C. IR (KBr, cm^{-1}): 3295 (NH), 3137 (=CH), 2922, 2853 (CH), 1613 (CO), 1582, 1562, 1511 (C=C), 1359, 1140 (SO_2). ^{1}H NMR (400.13 MHz, $CDCl_3$): δ 11.26 (br.s, 1H, NH, CO-pyrrole), 9.72 (br.s, 1H, NH, pyrrole), 7.68-7.67 (m, 1H, H-5 of thiophene), 7.65-7.60 (m, 5H, Ph, H-3, thiophene), 7.50-7.49 (m, 1H, H-5, CO-pyrrole), 7.40-7.36 (m, 2H, Ph), 7.22-7.18 (m, 1H, Ph), 7.16-7.14 (m, 2H, Ph), 7.12-7.10 (m, 1H, H-4, thiophene), 6.97 (dd, J = 3.6, 2.5 Hz, 1H, H-3, pyrrole), 6.55-6.54 (m, 1 7.2, 134.5, 134.3, 132.9, 132.4, 129.7 (2C), 128.9 (2C), 128.0, 127.6, 127.0 (2C), 126.2, 125.1, 123.9, 123.7 (2C), 122.5, 122.1, 114.5, 107.1, 21.5. Anal. Calcd for $C_{26}H_{20}N_2O_3S_2$: C, 66.08; H, 4.27; N, 5.93; S, 13.57%. Found: C, 66.25; H, 4.46; N, 6.07; S, 13.38%. HRMS (ESI-TOF): found 473,0994. Calcd. for $[C_{26}H_{20}N_2O_3S_2+H]^+$ 473,0998.

*(5-(4-(Dimethylamino)phenyl)-2'-tosyl-1H,1'H-[2,3'-bipyrrol]-4'-yl)(phenyl)methanone (**2k**)*

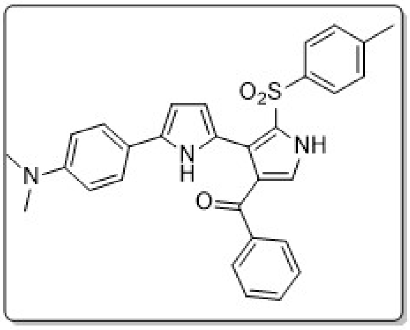

Yield 270 mg (53%). Yellow crystals, mp 213-215 °C. IR (KBr, cm^{-1}): 3240 (NH), 2924, 2853 (CH), 1613 (CO), 1512, 1491 (C=C), 1350, 1141 (SO_2). ^{1}H NMR (400.13 MHz, $CDCl_3$): δ 11.58 (br.s, 1H, NH, CO-pyrrole), 10.03 (br.s, 1H, NH, pyrrole), 7.75-7.73 (m, 2H, Ph), 7.67-6.65 (m, 2H, Ph), 7.54-7.52 (m, 3H, Ph), 7.44-7.40 (m, 2H, Ph), 7.24-7.19 (m, 1H, H-5, CO-pyrrole), 7.16-7.14 (m, 2H, Ph), 6.98-6.96 (m, 1H, H-4, pyrrole), 6.78-6.76 (m, 2H, Ph), 6.40-6.38 (m, 1H, H-3 pyrrole), 2.96 (s,

6H, $N(CH_3)_2$), 2.31 (s, 3H, CH_3). ^{13}C NMR (100.6 MHz, $CDCl_3$): δ 192.6, 149.4, 144.7, 139.7, 137.6, 133.9, 132.5, 130.0, 129.8 (2C), 129.7 (2C), 128.4 (2C), 127.1 (3C), 125.0 (2C), 124.3, 123.9, 123.2, 121.6, 121.3, 114.9, 113.2, 105.6, 40.8 (2C), 21.7. Anal. Calcd for $C_{30}H_{27}N_3O_3S$: C, 70.71; H, 5.34; N, 8.25; S, 6.29%. Found: C, 70.82; H, 5.48; N, 8.38; S, 6.18%. HRMS (ESI-TOF): found 510,1851. Calcd. for $[C_{30}H_{27}N_3O_3S+H]^+$ 510,1848.

*(5-(4-Fluorophenyl)-2'-tosyl-1H,1'H-[2,3'-bipyrrol]-4'-yl)(phenyl)methanone (**2l**)*

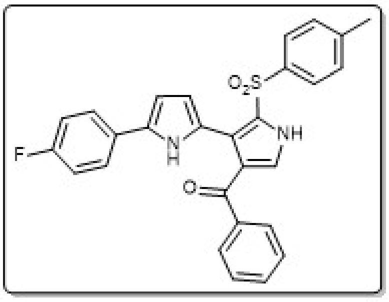

Yield 252 mg (52%). Yellow crystals, mp 130-132 °C. IR (KBr, cm^{-1}): 3233 (NH), 3063 (=CH), 2969 (CH), 1613 (CO), 1597, 1486 (C=C), 1351, 1145 (SO_2). ^1H NMR (400.13 MHz, DMSO-d_6): δ 11.79 (br.s, 1H, NH, CO-pyrrole), 9.94 (br.s, 1H, NH, pyrrole), 7.77-7.75 (m, 2H, Ph), 7.68-7.66 (m, 2H, Ph), 7.60-7.55 (m, 3H, Ph), 7.47-7.43 (m, 2H, Ph), 7.28 (d, J = 3.5 Hz, 1H, H-5, CO-pyrrole), 7.19-7.17 (m, 2H, Ph), 7.09-7.03 (m, 3H, H-4, pyrrole, Ph), 6.49-6.47 (m, 1H, H-3, pyrrole), 2.33 (s, 3H, CH_3). ^{13}C NMR (100.6 MHz DMSO-d_6): δ 190.4, 160.5 (d, J = 242.4 Hz), 144.1, 138.8 (d, J = 29.4 Hz, 2C), 131.9, 130.9, 130.1, 129.8 (2C), 129.3 (d, J = 2.8 Hz), 129.0 (2C), 128.1 (2C), 126.7, 126.5 (2C), 125.3 (d, J = 7.8 Hz, 2C), 123.6, 122.5, 121.7, 115.6, 115.4, 112.7, 106.2, 21.0. Anal. Calcd for $C_{28}H_{21}FN_2O_3S$: C, 69.41; H, 4.37; F, 3.92; N, 5.78; S, 6.62%. Found: C, 69.56; H, 4.50; N, 5.95; S, 6.51; F, 4.04%.

*(5-(4-Fluorophenyl)-2'-tosyl-1H,1'H-[2,3'-bipyrrol]-4'-yl)(furan-2-yl)methanone (**2m**)*

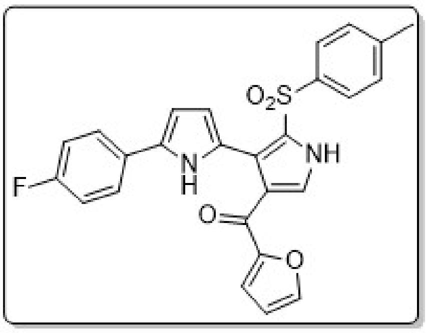

Yield 228 mg (48%). Yellow crystals, mp 118-120 °C. IR (KBr, cm^{-1}): 3281 (NH), 3152 (=CH), 2923, 2856 (CH), 1621 (CO), 1602, 1561, 1512, 1488 (C=C), 1351, 1141 (SO_2). ^1H NMR (400.13 MHz, $CDCl_3$): δ 11.58 (br.s, 1H, NH, CO-pyrrole), 7.72-7.70 (m, 1H, H-5, furan), 7.67-7.65 (m, 2H, Ph), 7.64-7.62 (m, 1H, H-3, furan), 7.59-7.55 (m, 2H, Ph), 7.17 (d, J = 3.6 Hz, 1H, H-5, CO-pyrrole), 7.16-7.14 (m, 2H, Ph), 7.08-7.03 (m, 3H, H-3 pyrrole, Ph), 6.54 (dd, J = 3.3, 1.5 Hz, 1H, H-4, furan), 6.49-6.48 (m, 1H, H-4 pyrrole), 2.31 (s, 3H, CH_3). ^{13}C NMR (100.6 MHz, $CDCl_3$): δ 178.0, 161.7 (d, J = 245.4 Hz), 153.1, 147.3, 144.9, 137.5, 132.2, 129.8 (2C), 129.0, 128.9 (d, J = 2.8 Hz), 127.2 (2C), 125.5 (d, J = 7.9 Hz, 2C), 125.1, 122.8, 122.7, 122.7, 120.5, 115.9 (d, J = 21.8 Hz, 2C), 114.8, 112.6, 107.1, 21.7. Anal. Calcd for $C_{26}H_{19}FN_2O_4S$: C, 65.81; H, 4.04; F, 4.00; N, 5.90; S, 6.76%. Found: C, 65.99; H, 4.21; F, 4.18; N, 6.09; S, 6.66%. HRMS (ESI-TOF): found 475,1128. Calcd. for $[C_{26}H_{19}FN_2O_4S+H]^+$ 475,1133.

*(5-(4-Chlorophenyl)-2'-tosyl-1H,1'H-[2,3'-bipyrrol]-4'-yl)(phenyl)methanone (**2n**)*

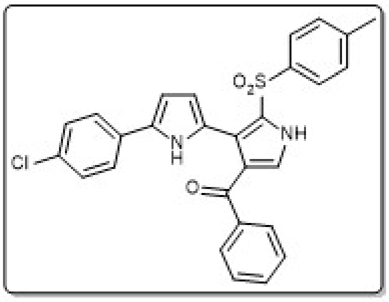

Yield 225 mg (45%). Yellow crystals, mp 218-220 °C. IR (KBr, cm^{-1}): 3275 (NH), 3118, 3092 (=CH), 1630 (CO), 1596, 1576, 1505, 1477 (C=C), 1351, 1145 (SO_2). ^1H NMR (400.13 MHz, $CDCl_3$): δ 11.83 (br.s, 1H, NH, CO-pyrrole), 9.89 (br.s, 1H, NH, pyrrole), 7.78-7.76 (m, 2H, Ph), 7.68-7.66

(m, 2H, Ph), 7.59-7.54 (m, 3H, Ph), 7.47-7.43 (m, 2H, Ph), 7.34-7.32 (m, 2H, Ph), 7.29 (d, J = 3.5 Hz, 1H, H-5, CO-pyrrole), 7.19-7.17 (m, 2H, Ph), 7.05 (dd, J = 3.6, 2.4 Hz, 1H, H-4, pyrrole), 6.54-6.52 (m, 1H, H-3, pyrrole), 2.33 (s, 3H, CH_3). ^{13}C NMR (100.61 MHz, $CDCl_3$): δ 192.8, 144.9, 139.6, 137.4, 132.7, 131.8, 131.7, 131.1, 130.3, 129.9 (2C), 129.8 (2C), 129.2 (2C), 128.5 (2C), 127.1 (2C), 125.1, 125.0 (2C), 124.0, 123.2, 122.6, 114.9, 107.8, 21.7. Anal. Calcd for $C_{28}H_{21}ClN_2O_3S$: C, 67.13; H, 4.23; Cl, 7.08; N, 5.59; S, 6.40%. Found: C, 67.33; H, 4.38; N, 5.70; S, 6.29%. HRMS (ESI-TOF): found 501,1040. Calcd. for $[C_{28}H_{21}ClN_2O_3S+H]^+$ 501,1044.

*(4,5-Diphenyl-2'-tosyl-1H,1'H-[2,3'-bipyrrol]-4'-yl)(phenyl)methanone (**2o**)*

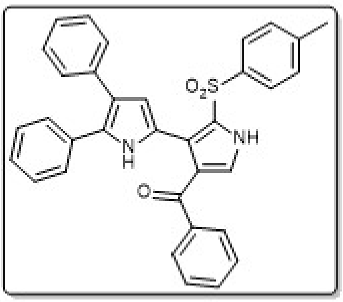

Yield 250 mg (46%). Yellow crystals, mp 203-205 °C. IR (KBr, cm^{-1}): 3296 (NH), 3059 (=CH), 2923 (CH), 1632 (CO), 1599, 1508, 1477 (C=C), 1351, 1132 (SO_2). ^1H NMR (400.13 MHz, $CDCl_3$): δ 11.36 (br.s, 1H, NH, CO-pyrrole), 9.82 (br.s, 1H, NH, pyrrole), 7.80-7.77 (m, 2H, Ph), 7.73-7.70 (m, 2H, Ph), 7.61-7.55 (m, 1H, H-5, CO-pyrrole), 7.48-7.45 (m, 4H, Ph), 7.36-7.27 (m, 7H, Ph), 7.24-7.20 (m, 4H, Ph), 7.05-7.03 (m, 1H, pyrrole), 2.36 (s, 3H, CH_3). ^{13}C NMR (100.6 MHz, $CDCl_3$): δ 192.6, 144.9, 139.6, 137.4, 137.0, 133.2, 132.7, 129.8 (3C), 129.6, 129.2, 128.8 (2C), 128.7 (2C), 128.5 (2C), 128.4 (2C), 127.4 (2C), 127.1 (2C), 126.8, 126.0, 125.5, 124.4, 123.6, 122.4, 121.8, 120.8, 115.7, 21.7. Anal. Calcd for $C_{34}H_{26}N_2O_3S$: C, 74.95; H, 4.68; N, 5.13; S, 5.91%. Found: C, 75.45; H, 4.98; N, 5.30; S, 5.79%. HRMS (ESI-TOF): found 543,1742. Calcd. for $[C_{34}H_{26}N_2O_3S+H]^+$ 543,1739.

4.2. Characterization data of pyrrolo[1,2-*c*]imidazoles 3a-j

*(E)-1-Phenyl-2-(1H-pyrrolo[1,2-c]imidazol-1-ylidene)-3-tosylpropan-1-one (**3a**)*

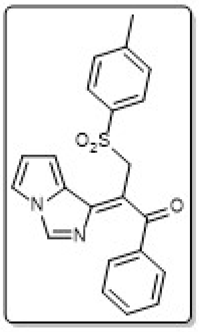

Yield 144 mg (37%). Yellow crystals, mp 183-185°C. IR (KBr, cm^{-1}): 3063(=CH), 2923, 1689 (CO), 1596, 1581, 1523, 1493 (C=C), 1320, 1153 (SO_2). ^1H NMR (400.13 MHz, $CDCl_3$): δ 8.64 (s, 1H, H-3, pyrroloimidazole), 8.14-8,12 (m, 2H, Ph), 7.90-7.88 (m, 2H, Ph), 7.66-7.62 (m, 1H, Ph), 7.56-7.53 (m, 2H, Ph), 7.44-7.43 (m, 1H, H-5, pyrroloimidazole), 7.29-7.27 (m, 2H, Ph), 6.94-6.93 (m, 1H, H-6, pyrroloimidazole), 6.67-6.65 (m, 1H, H-7, pyrroloimidazole, 5.17 (s, 2H, CH_2), 2.39 (s, 3H, CH_3). ^{13}C NMR (100.6 MHz, $CDCl_3$): δ 195.2, 144.4, 138.3, 137.6, 136.9, 136.8, 133.7, 132.1, 129.6 (2C), 128.9 (2C), 128.8 (2C), 128.5 (2C), 124.2, 118.3, 114.2, 104.8, 38.1, 21.8. Anal. Calcd for $C_{22}H_{18}N_2O_3S$: C, 67.68; H, 4.65; N, 7.17; S, 8.21 %. Found: C, 67.80; H, 4.83; N, 7.30; S, 8.09%. HRMS (ESI-TOF): found 391,1116. Calcd. for $[C_{22}H_{18}O_3SN_2+H]^+$ 391,1120.

*(E)-1-(Furan-2-yl)-2-(1H-pyrrolo[1,2-c]imidazol-1-ylidene)-3-tosylpropan-1-one (**3b**)*

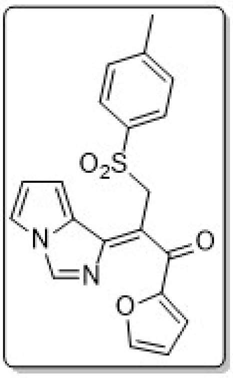

Yield 133 mg (35%). Yellow crystals, mp 90-92 °C. IR (KBr, cm^{-1}): 3132 (=CH), 2923, 2853 (CH), 1679 (CO), 1621, 1595, 1521, 1467 (C=C), 1320, 1153 (SO_2). ^1H NMR (400.13 MHz, $CDCl_3$): δ 8.63 (s, 1H, H-3, pyrroloimidazole), 7.93-7.91 (m, 2H, Ph), 7.66-7.67 (m, 1H, H-5, furan), 7.43-7.41 (m, 1H, H-3, furan), 7.38-7.37 (m, 1H, H-5, pyrroloimidazole), 7.30-7.28 (m, 2H, Ph), 6.94-6.92 (m, 1H, H-6, pyrroloimidazole), 6.72-6.71 (m, 1H, H-7, pyrroloimidazole), 6.62 (dd, J = 3.4, 1.5 Hz, 1H, H-4, furan), 5.05 (s, 2H, CH_2), 2.40 (s, 3H, CH_3). ^{13}C NMR (100.6 MHz, $CDCl_3$): δ 184.1, 152.4, 146.8, 144.4, 138.5, 137.6, 136.9, 132.0, 129.6 (2C), 128.9 (2C), 123.2, 118.4, 117.8, 114.2, 112.7, 104.9, 37.6, 21.8. Anal. Calcd for $C_{20}H_{16}N_2O_4S$: C, 63.30; H, 4.24; N, 7.21; S, 8.43%. Found: C, 63.30; H, 4.39; N, 7.48; S, 8.31%. HRMS (ESI-TOF): found 381,0909. Calcd. for $[C_{20}H_{16}N_2O_4S+H]^+$ 381,0911.

*(E)-2-(6-Ethyl-5-propyl-1H-pyrrolo[1,2-c]imidazol-1-ylidene)-1-phenyl-3-tosylpropan-1-one (**3c**)*

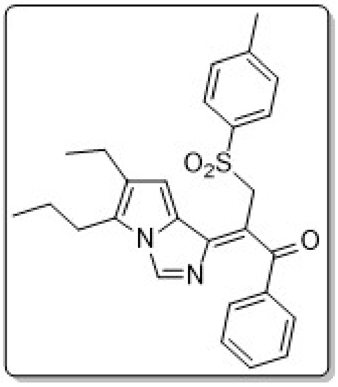

Yield 147 mg (32%). Yellow crystals, mp 153-155°C. IR (KBr, cm^{-1}): 3129 (=CH), 2963, 2932, 2872 (CH), 1690 (CO), 1597, 1581, 1495 (C=C), 1318, 1129 (SO_2). ^1H NMR (400.13 MHz, $CDCl_3$): δ 8.51 (s, 1H, H-3, pyrroloimidazole), 8.15-8,13 (m, 2H, Ph), 7.90-7.88 (m, 2H, Ph), 7.65-7.61 (m, 1H, Ph), 7.56-7.52 (m, 2H, Ph), 7.28-7.26 (m, 2H, Ph), 6.55-6.53 (m, 1H, H-6, pyrroloimidazole), 5.13 (s, 2H, CH_2), 2.85-2.81 (m, 2H, CH_2), 2.62-2.57 (m, 2H, CH_2), 2.38 (s, 3H, CH_3), 1.63-1.57 (m, 2H, CH_2), 1.19 (t, J =7.5 Hz, 3H, CH_3), 0.94 (t, J = 7.3 Hz, 3H, CH_3). ^{13}C NMR (100.6 MHz, $CDCl_3$): δ 195.5, 144.1, 137.9, 136.9, 136.6, 133.9, 133.8, 133.5, 130.9, 129.5 (2C), 128.9 (2C), 128.7 (2C), 128.5 (2C), 124.3, 123.1, 103.9, 38.2, 25.6, 21.7, 21.6, 19.5, 15.2, 14.0. Anal. Calcd for $C_{27}H_{28}N_2O_3S$: C, 70.41; H, 6.13; N, 6.08; S, 6.96%. Found: C, 70.14; H, 6.15; N, 6.98; S, 6.77%. HRMS (ESI-TOF): found 461,1899. Calcd. for $[C_{27}H_{28}N_2O_3S+H]^+$ 461,1900.

*(E)-1-Phenyl-2-(5-phenyl-1H-pyrrolo[1,2-c]imidazol-1-ylidene)-3-tosylpropan-1-one (**3d**)*

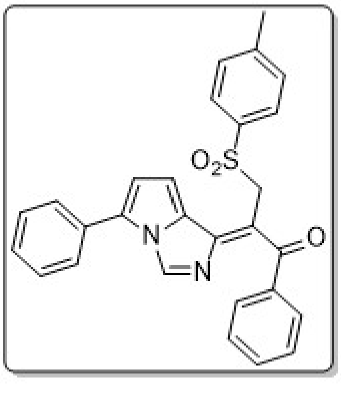

Yield 149 mg (32%). Yellow crystals, mp 165-167°C. IR (KBr, cm^{-1}): 3134, 3061 (=CH), 2923, 2853 (CH), 1688 (CO), 1596, 1455 (C=C), 1318, 1145 (SO_2). ^1H NMR (400.13 MHz, $CDCl_3$): δ 8.91 (s, 1H, H-3, pyrroloimidazole), 8.17-8.15 (m, 2H, Ph), 7.90-7.88 (m, 2H, Ph), 7.68-7.64 (m, 1H, Ph), 7.58-7.55 (m, 2H, Ph), 7.49-7.48 (m, 4H, Ph), 7.43-7.39 (m, 1H, Ph), 7.29-7.27 (m, 2H, Ph), 6.98 (d, J = 3.7 Hz, 1H, H-6, pyrroloimidazole), 6.76 (d, J = 3.7 Hz, 1H, H-7, pyrroloimidazole), 5.20 (s, 2H, CH_2), 2.39 (s, 3H, CH_3). ^{13}C NMR (100.61 MHz, $CDCl_3$): δ 195.4, 144.4, 138.3, 137.6, 136.9, 135.1, 133.7, 132.8, 130.1, 129.5 (2C), 129.4 (2C), 129.0 (2C), 128.9 (2C), 128.8, 128.6 (3C), 128.5 (2C), 124.2, 118.2,

105.2, 38.1, 21.8. Anal. Calcd for $C_{28}H_{22}N_2O_3S$: C, 72.08; H, 4.75; N, 6.00; S, 6.87%. Found: C, 72.24; H, 4.94; N, 6.15; S, 6.74%. HRMS (ESI-TOF): found 467,1429. Calcd. for $[C_{28}H_{22}N_2O_3S+H]^+$ 467,1435.

*(E)-1-(Furan-2-yl)-2-(5-phenyl-1H-pyrrolo[1,2-c]imidazol-1-ylidene)-3-tosylpropan-1-one (**3e**)*

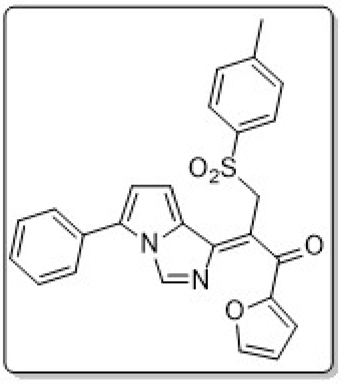

Yield 160 mg (35%). Yellow crystals, mp 183-185°C. IR (KBr, cm^{-1}): 3133, 3063 (=CH), 2924 (CH), 1680 (CO), 1596, 1589, 1537 (C=C), 1317, 1145 (SO_2). ^1H NMR (400.13 MHz, $CDCl_3$): δ 8.90 (s, 1H, H-3, pyrroloimidazole), 7.95-7.93 (m, 2H, Ph), 7.70-7.69 (m, 1H, H-5, furan), 7.52-7.48 (m, 4H, Ph), 7.42-7.41 (m, 2H, *p*-Ph, H-3, furan), 7.31-7.29 (m, 2H, Ph), 6.99 (d, J = 4.1 Hz, 1H, H-6, pyrroloimidazole), 6.82 (d, J = 4.1 Hz, 1H, H-7, pyrroloimidazole), 6.64 (dd, J = 3.6, 1.7 Hz, 1H, H-4, furan), 5.09 (s, 2H, CH_2), 2.40 (s, 3H, CH_3). ^{13}C NMR (100.6 MHz, $CDCl_3$): δ 184.0, 152.2, 146.6, 144.2, 138.3, 137.4, 134.9, 132.5, 129.8, 129.3 (2C), 129.2 (2C), 128.8 (2C), 128.7, 128.6, 128.4 (2C), 123.0, 118.0, 117.6, 112.5, 105.1, 37.5, 21.6. Anal. Calcd for $C_{26}H_{20}N_2O_4S$: C, 68.04; H, 4.42; N, 6.14; S, 7.02%. Found: C, 68.54; H, 4.56; N, 6.27; S, 6.88%. HRMS (ESI-TOF): found 457,1222. Calcd. for $[C_{26}H_{20}N_2O_4S+H]^+$ 457,1229.

*(E)-2-(5-Phenyl-1H-pyrrolo[1,2-c]imidazol-1-ylidene)-1-(thiophen-2-yl)-3-tosylpropan-1-one (**3f**)*

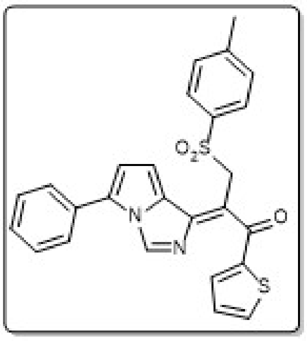

Yield 175 mg (37%). Yellow crystals, mp 155-157°C. IR (KBr, cm^{-1}): 3120, 3097 (=CH), 2861 (CH), 1663 (CO), 1595, 1518, 1494 (C=C), 1318, 1122 (SO_2). ^1H NMR (400.13 MHz, $CDCl_3$): δ 8.87 (s, 1H, H-3, pyrroloimidazole), 7.99-7.98 (m, 1H, H-5, thiophene), 7.90-7.87 (m, 2H, Ph), 7.70-7.68 (m, 1H, H-3, thiophene), 7.45-7.40 (m, 4H, Ph), 7.39-7.34 (m, 1H, Ph), 7.27-7.25 (m, 2H, Ph), 7.21-.7.18 (m, 1H, H-3, thiophene), 6.94-6.93 (m, 1H, H-6, pyrroloimidazole), 6.78-6.77 (m, 1H, H-7, pyrroloimidazole), 5.12 (s, 2H, CH_2), 2.35 (s, 3H, CH_3). ^{13}C NMR (100.6 MHz, $CDCl_3$): δ 187.9, 144.4, 143.5, 138.3, 137.5, 135.1, 134.2, 132.8, 132.6, 129.9, 129.5 (2C), 129.4 (2C), 128.9, 128.8 (2C), 128.6, 128.5 (2C), 128.4, 123.4, 118.2, 105.4, 38.4, 21.7. Anal. Calcd for $C_{26}H_{20}N_2O_3S_2$: C, 66.08; H, 4.27; N, 5.93; S, 13.57%. Found: C, 65.91; H, 4.05; N, 6.11; S, 13.39%. HRMS (ESI-TOF): found 473,0994. Calcd. for $[C_{26}H_{20}N_2O_3S_2+H]^+$ 473,0995.

*(E)-2-(5-(4-(Dimethylamino)phenyl)-1H-pyrrolo[1,2-c]imidazol-1-ylidene)-1-phenyl-3-tosylpropan-1-one (**3g**)*

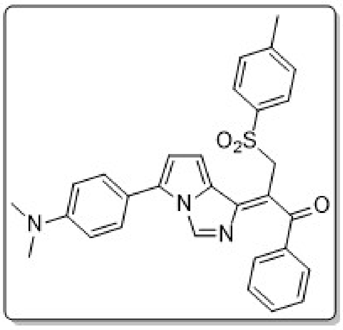

Yield 143 mg (28%). Yellow crystals, mp 108-110°C. IR (KBr, cm^{-1}): 3018 (=CH), 2924 (CH), 1688 (CO), 1612, 1597 (C=C), 1318, 1145 (SO_2). ^1H NMR (400.13 MHz, $CDCl_3$): δ 8.89 (s, 1H, H-3, pyrroloimidazole), 8.17-8.15 (m, 2H, Ph), 7.90-7.88 (m, 2H, Ph), 7.67-7.62 (m, 1H, Ph), 7.57-7.54 (m, 2H, Ph), 7.35-7.33 (m, 2H, Ph), 7.28-7.26 (m, 2H, Ph), 6.87 (d, J = 3.9 Hz, 1H, H-6, pyrroloimidazole), 6.79-6.77 (m, 2H, Ph), 6.73 (d, J = 3.9 Hz, 1H, H-7,

pyrroloimidazole), 5.18 (s, 2H, CH_2), 3.02 (s, 6H, $N(CH_3)_2$), 2.39 (s, 3H, CH_3). ^{13}C NMR (100.6 MHz, $CDCl_3$): δ 195.5, 150.6, 144.2, 137.8, 137.4, 136.9, 135.3, 133.6, 132.0, 130.5, 129.8, 129.6 (3C), 129.5 (2C), 128.9 (2C), 128.8 (2C), 128.5 (3C), 124.2, 117.3, 112.6, 105.1, 38.1 (2C), 21.8. Anal. Calcd for $C_{30}H_{27}N_3O_3S$: C, 70.71; H, 5.34; N, 8.25; S, 6.29%. Found: C, 70.85; H, 5.53; N, 8.39; S, 6.12%. HRMS (ESI-TOF): found 510,1851. Calcd. for $[C_{30}H_{27}N_3O_3S+H]^+$ 510,1856.

*(E)-2-(5-(4-Fluorophenyl)-1H-pyrrolo[1,2-c]imidazol-1-ylidene)-1-phenyl-3-tosylpropan-1-one (**3h**)*

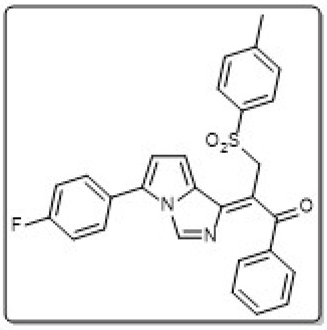

Yield 184 mg (38%). Yellow crystals, mp 95-97°C. IR (KBr, cm^{-1}): 3131, 3063 (=CH), 2923 (CH), 1689 (CO), 1596, 1539, 1510, 1493 (C=C), 1316, 1146 (SO_2). ^1H NMR (400.13 MHz, $CDCl_3$): *δ* 8.81 (s, 1H, H-3, pyrroloimidazole), 8.16-8,14 (m, 2H, Ph), 7.90-7.88 (m, 2H, Ph), 7.67-7.63 (m, 1H, Ph), 7.58-7.54 (m, 2H, Ph), 7.47-7.44 (m, 2H, Ph), 7.29-7.27 (m, 2H, Ph), 7.20-7.16 (m, 2H, Ph), 6.93 (d, *J* = 4.0 Hz, 1H, H-6, pyrroloimidazole), 6.75 (d, *J* = 4.0 Hz, 1H, H-7, pyrroloimidazole), 5.20 (s, 2H, CH_2), 2.39 (s, 3H, CH_3). ^{13}C NMR (100.6 MHz, CDCl3): δ 195.3, 162.9 (d, $^1J_{CF}$ = 250.5 Hz, C4, 4F-C_6H_4), 144.4, 138.3, 137.5, 136.8, 134.8, 133.7, 132.7, 130.6 (d, $^3J_{CF}$ = 8.3 Hz, C-2,6 4F-C_6H_4, 2C), 129.5 (2C), 129.0 (2C), 128.9 (2C), 128.5 (2C), 127.4, 126.1 (d, $^4J_{CF}$ = 3.3 Hz, C-1 4F-C_6H_4), 124.2, 118.2, 116.6 (d, $^2J_{CF}$ = 21.8 Hz, C-3,5 4F-C_6H_4, 2C), 105.1, 38.1, 21.8. Anal. Calcd for $C_{28}H_{21}FN_2O_3S$: C, 69.41; H, 4.37; F, 3.92; N, 5.78; S, 6.62%. Found: C, 69.52; H, 4.51; N, 5.97; S, 6.47; F, 4.04%. HRMS (ESI-TOF): found 485,1335. Calcd. for $[C_{28}H_{21}FN_2O_3S+H]^+$ 485,1339.

*(E)-2-(5-(4-Fluorophenyl)-1H-pyrrolo[1,2-c]imidazol-1-ylidene)-1-(furan-2-yl)-3-tosylpropan-1-one (**3i**)*

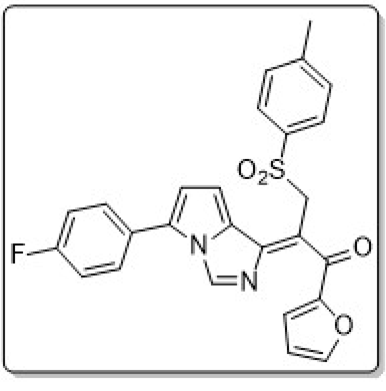

Yield 157 mg (33%). Yellow crystals, mp 188-190°C. IR (KBr, cm^{-1}): 3131, 3056 (=CH), 2925 (CH), 1680 (CO), 1602, 1561, 1570, 1540, 1513 (C=C), 1317, 1161 (SO_2). ^1H NMR (400.13 MHz, $CDCl_3$): *δ* 8.80 (s, 1H, H-3, pyrroloimidazole), 7.93-7,91 (m, 2H, Ph), 7.68-7.67 (m, 1H, H-5, furan), 7.46-7.43 (m, 2H, H-3, furan), 7.41-7.40 (m, 1H, Ph), 7.30-7.28 (m, 2H, Ph), 7.19-7.15 (m, 2H, Ph), 6.93 (d, *J* = 4.0 Hz, 1H, H-6, pyrroloimidazole), 6.79 (d, *J* = 4.0 Hz, 1H, H-7, pyrroloimidazole), 6.63 (dd, *J* = 2.5, 1.4 Hz, 1H, H-4, furan), 5.08 (s, 2H, CH_2), 2.39 (s, 3H, CH_3). ^{13}C NMR (100.6 MHz, $CDCl_3$): *δ* 184.1, 162.9 (d, $^1J_{CF}$ = 249.8 Hz, C4, 4F-C_6H_4), 152.3, 146.8, 144.4, 138.5, 137.5, 134.9, 132.6, 130.5 (d, $^3J_{CF}$ = 8.3 Hz, C-2,6 4F-C_6H_4, 2C), 129.5 (2C), 128.9 (2C), 127.4, 126.1 (d, $^4J_{CF}$ = 3.4 Hz, C-1 4F-C_6H_4), 123.2, 118.2, 117, 116.6 (d, $^2J_{CF}$ = 21.8 Hz, C-3,5 4F-C_6H_4, 2C), 112.7, 105.3, 37.6, 21.7. Anal. Calcd for $C_{26}H_{19}FN_2O_4S$: C, 65.81; H, 4.04; F, 4.00; N, 5.90; S, 6.76%. Found: C, 65.99; H, 4.19; N, 6.10; S, 6.60; F, 4.12 %. HRMS (ESI-TOF): found 475,1128. Calcd. for $[C_{26}H_{19}FN_2O_4S+H]^+$ 475,1128.

(E)-2-(5-(4-chlorophenyl)-1H-pyrrolo[1,2-c]imidazol-1-ylidene)-1-phenyl-3-tosylpropan-1-one (***3j***)

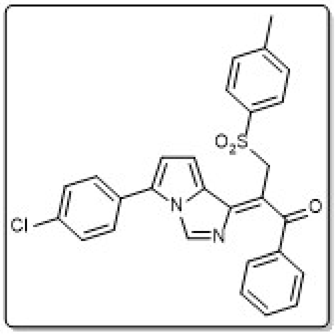

Yield 160 mg (32%). Yellow crystals, mp 135-137°C. IR (KBr, cm^{-1}): 3056 (=CH), 2924 (CH), 1688 (CO), 1596, 1581, 1532, 1494 (C=C), 1315, 1145 (SO_2). ^1H NMR (400.13 MHz, $CDCl_3$): δ 8.84 (s, 1H, H-3, pyrroloimidazole), 8.16-8,14 (m, 2H, Ph), 7.90-7.88 (m, 2H, Ph), 7.67-7.64 (m, 1H, Ph), 7.58-7.54 (m, 2H, Ph), 7.47-7.41 (m, 4H, Ph), 7.29-7.27 (m, 2H, Ph), 6.96 (d, J = 4.1 Hz, 1H, H-6, pyrroloimidazole), 6.76 (d, J = 4.1 Hz, 1H, H-7, pyrroloimidazole), 5.20 (s, 2H, CH_2), 2.39 (s, 3H, CH_3). ^{13}C NMR (100.6 MHz, $CDCl_3$): δ 194.7, 143.8, 137.9, 136.8, 136.1, 134.3, 134.2, 133.1, 132.4, 129.1 (2C), 129.0 (2C), 128.9 (2C), 128.3 (2C), 128.2 (2C), 127.9 (2C), 127.8, 126.6, 123.6, 117.7, 104.6, 37.4, 21.1. Anal. Calcd for $C_{28}H_{21}ClN_2O_3S$: C, 67.13; H, 4.23; Cl, 7.08; N, 5.59; S, 6.40%. Found: C, 67.25; H, 4.39; N, 5.78; S, 6.25%.

(1) Trofimov, B. A.; Stepanova, Z. V.; Sobenina, L. N.; Mikhaleva, A. b. I.; Ushakov, I. A., Ethynylation of pyrroles with 1-acyl-2-bromoacetylenes on alumina: a formal 'inverse Sonogashira coupling'. Tetrahedron Lett. 2004, 45, 6513-6516.

5. The NMR spectra of 2,3-bipyrroles (2a-o) and pyrrolo[1,2-c]imidazoles (3a-j) and HRMS spectra

^{1}H NMR spectrum of phenyl(2'-tosyl-1*H*,1'*H*-[2,3'-bipyrrol]-4'-yl)methanone (**2a**) in CDCl$_3$.

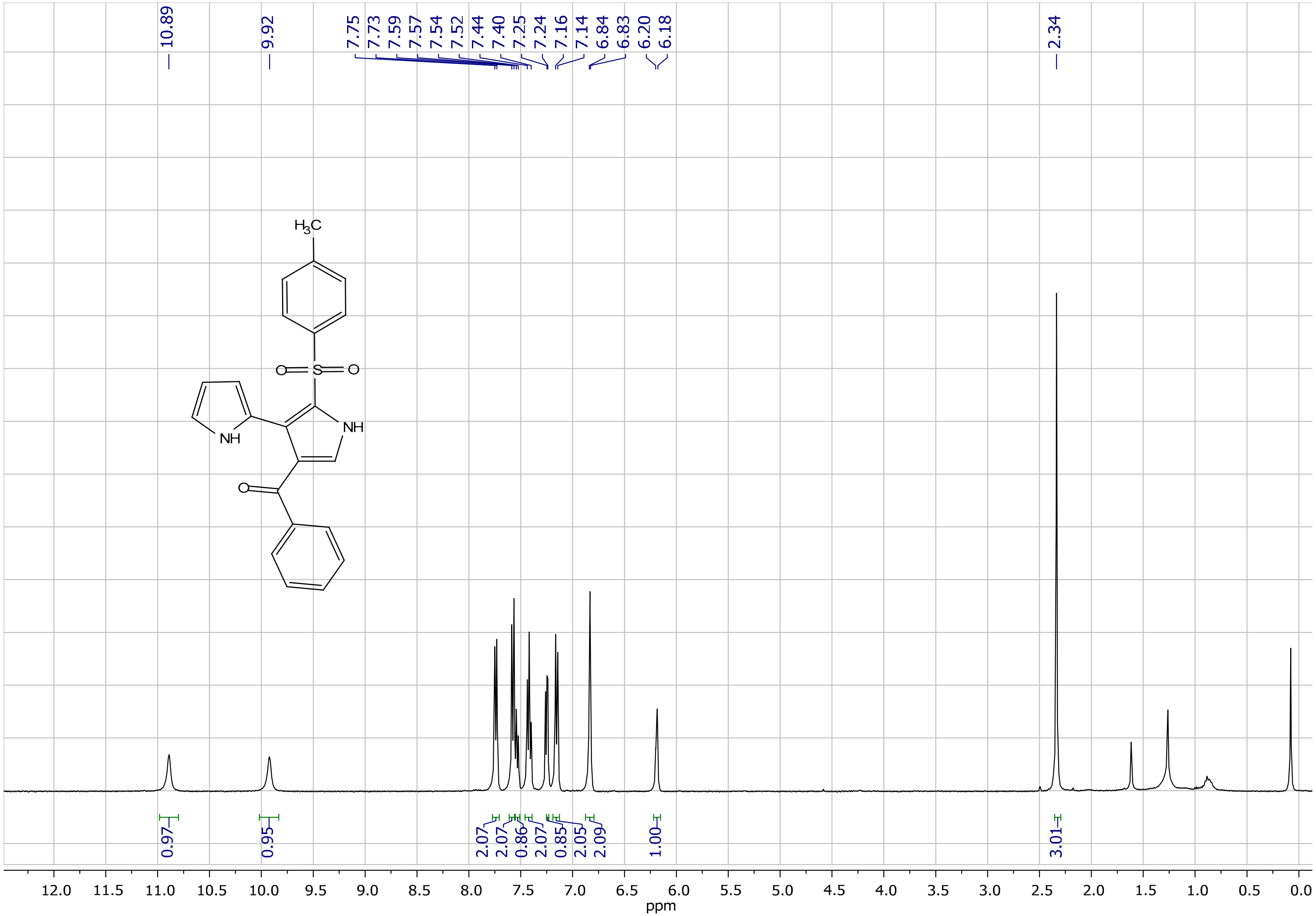

^{13}C NMR spectrum of phenyl(2'-tosyl-1*H*,1'*H*-[2,3'-bipyrrol]-4'-yl)methanone (**2a**) in $CDCl_3$.

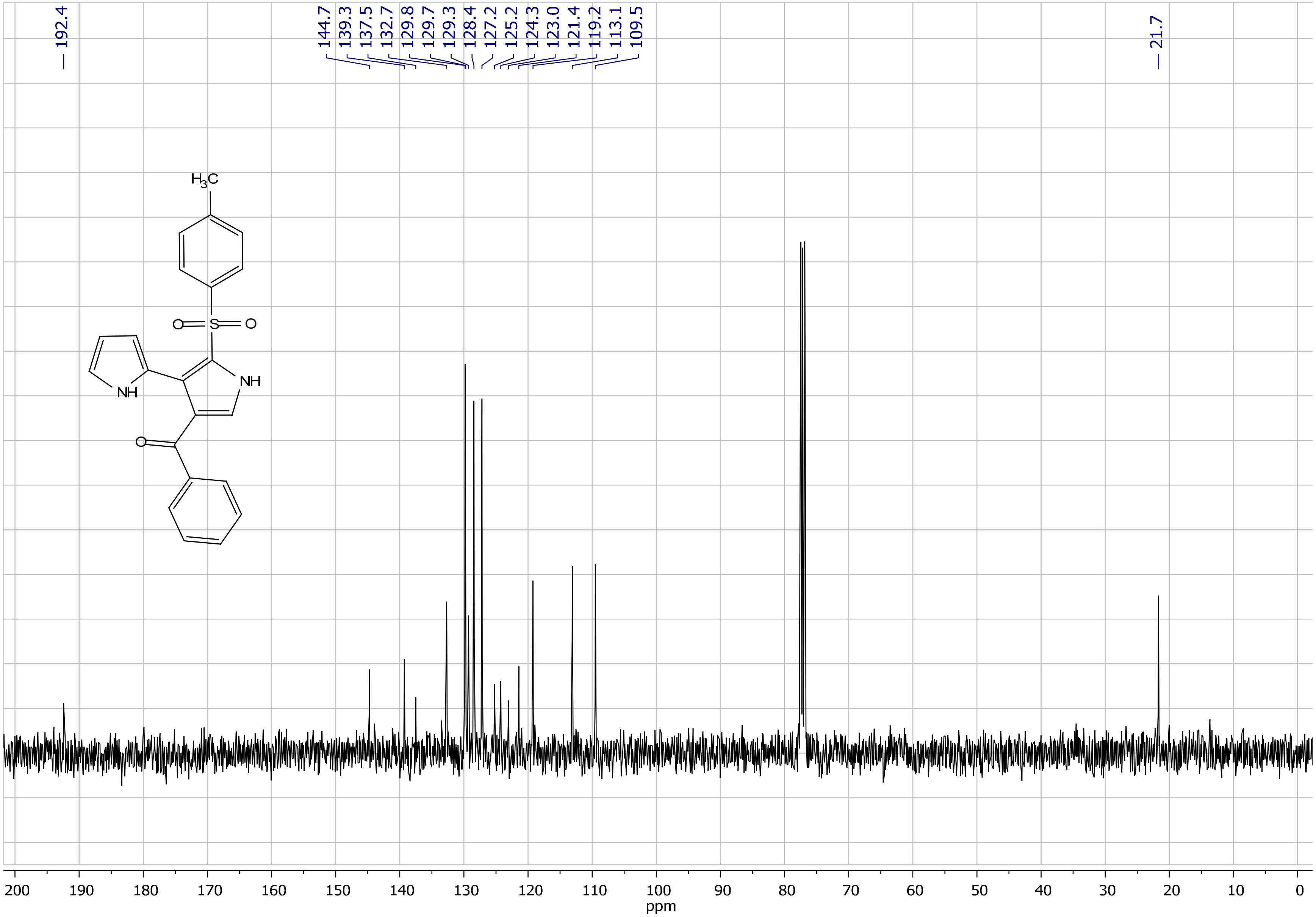

HRMS spectrum of phenyl(2'-tosyl-1*H*,1'*H*-[2,3'-bipyrrol]-4'-yl)methanone (**2a**)

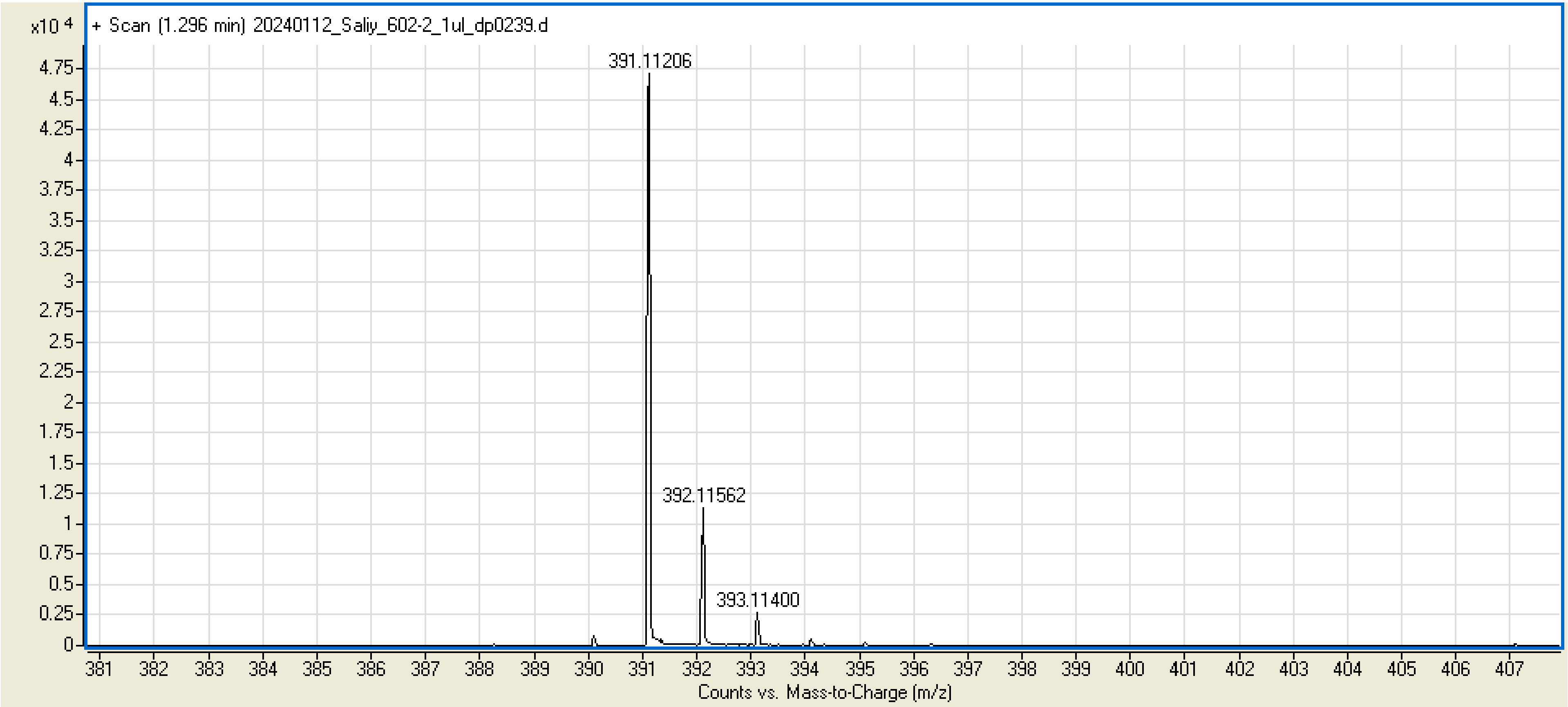

^{1}H NMR spectrum of furan-2-yl(2'-tosyl-1*H*,1'*H*-[2,3'-bipyrrol]-4'-yl)methanone (**2b**) in CDCl3.

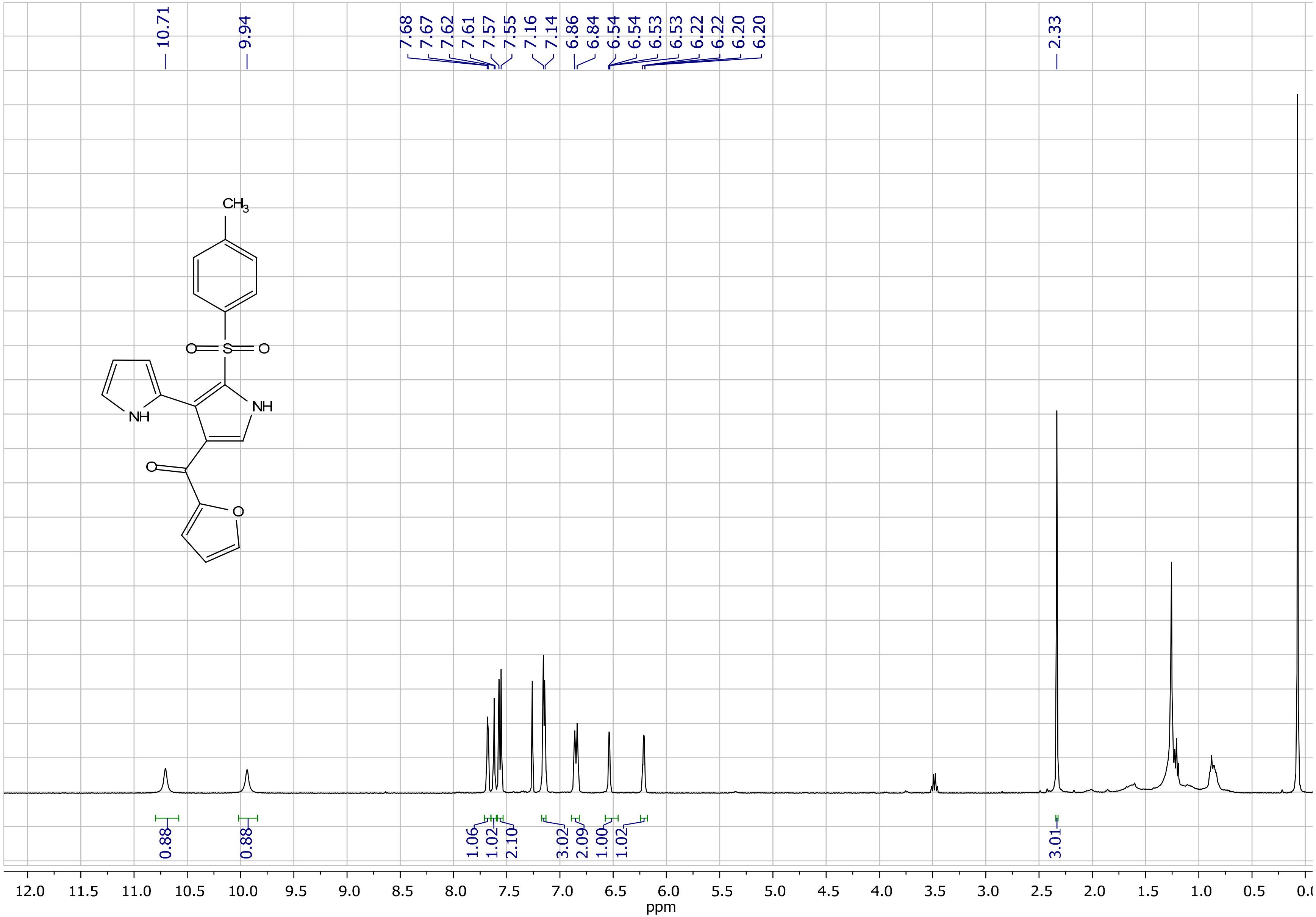

^{13}C NMR spectrum of furan-2-yl(2'-tosyl-1*H*,1'*H*-[2,3'-bipyrrol]-4'-yl)methanone (**2b**) in CDCl3.

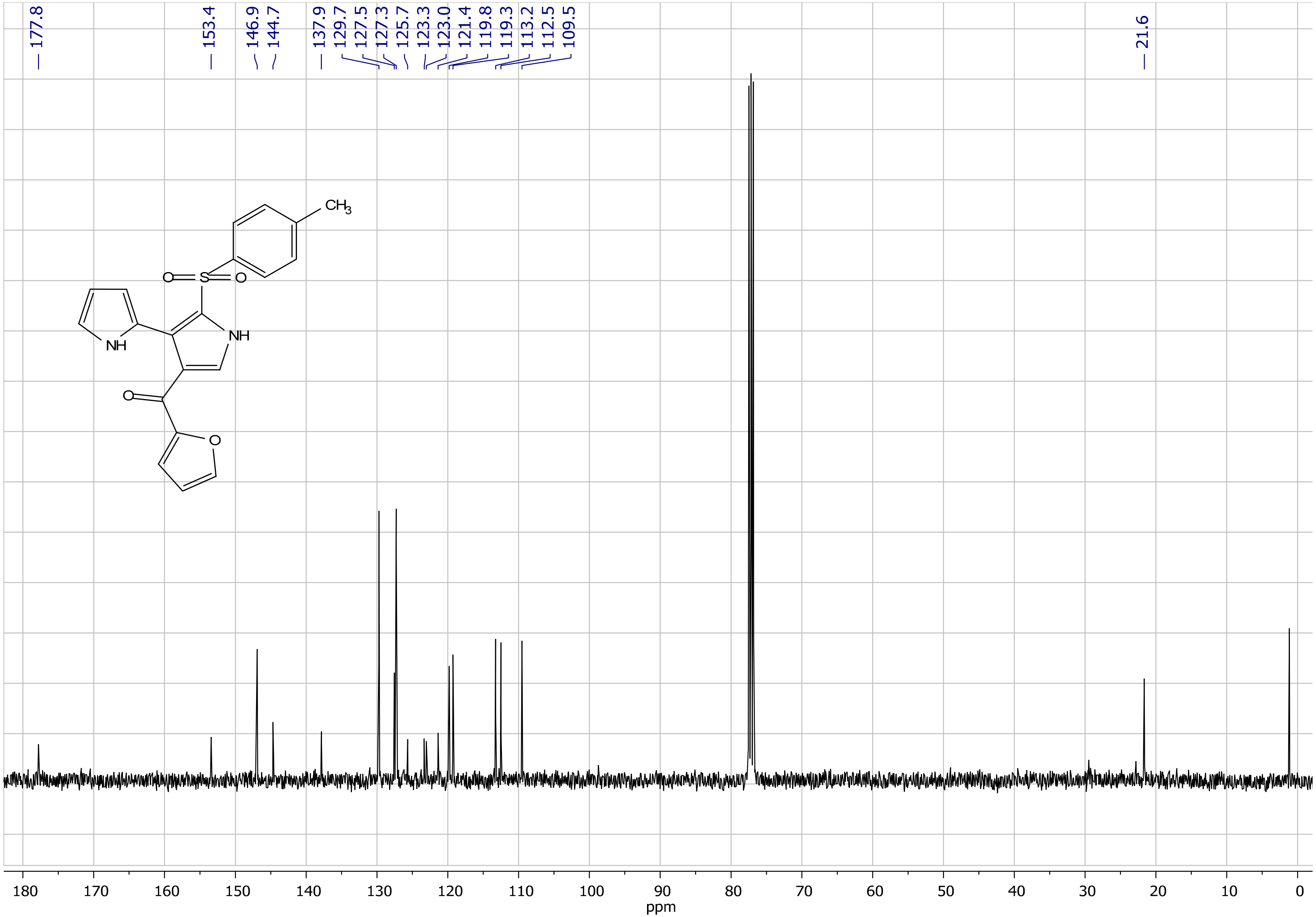

^{1}H NMR spectrum of thiophen-2-yl(2'-tosyl-1*H*,1'*H*-[2,3'-bipyrrol]-4'-yl)methanone (**2c**) in $CDCl_3$.

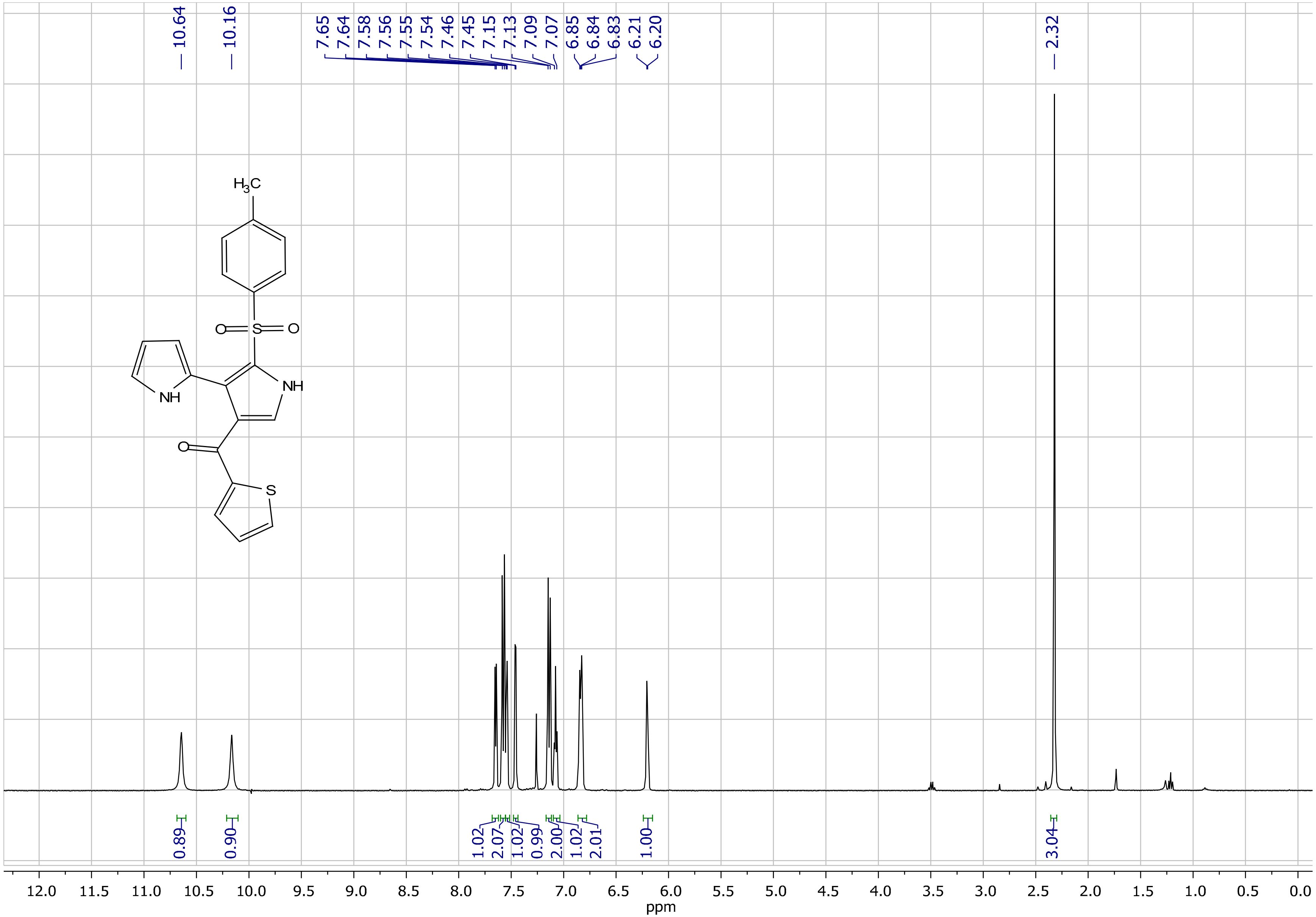

^{13}C NMR spectrum of thiophen-2-yl(2'-tosyl-1*H*,1'*H*-[2,3'-bipyrrol]-4'-yl)methanone (**2c**) in $CDCl_3$.

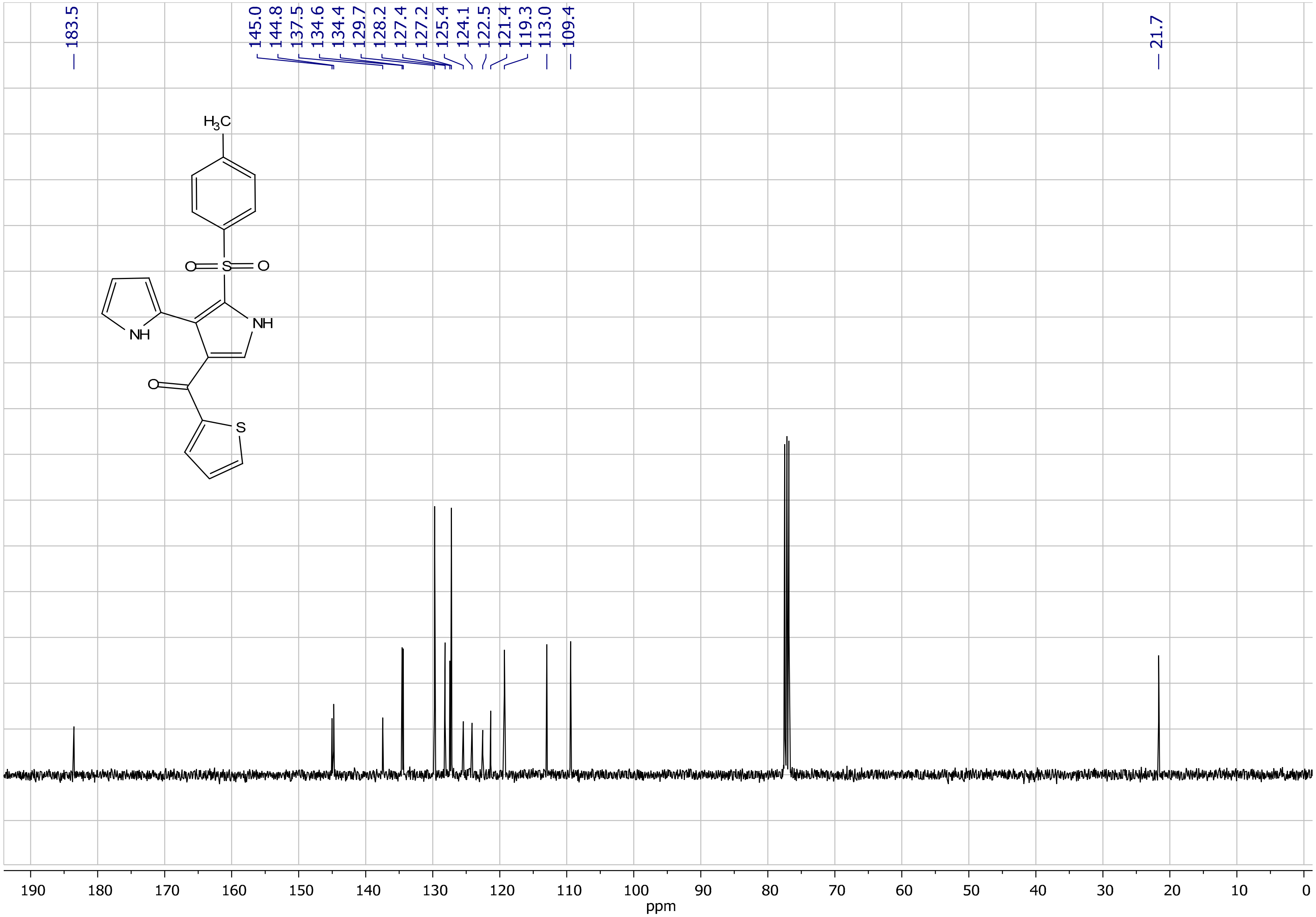

HRMS spectrum of thiophen-2-yl(2'-tosyl-1*H*,1'*H*-[2,3'-bipyrrol]-4'-yl)methanone (**2c)**

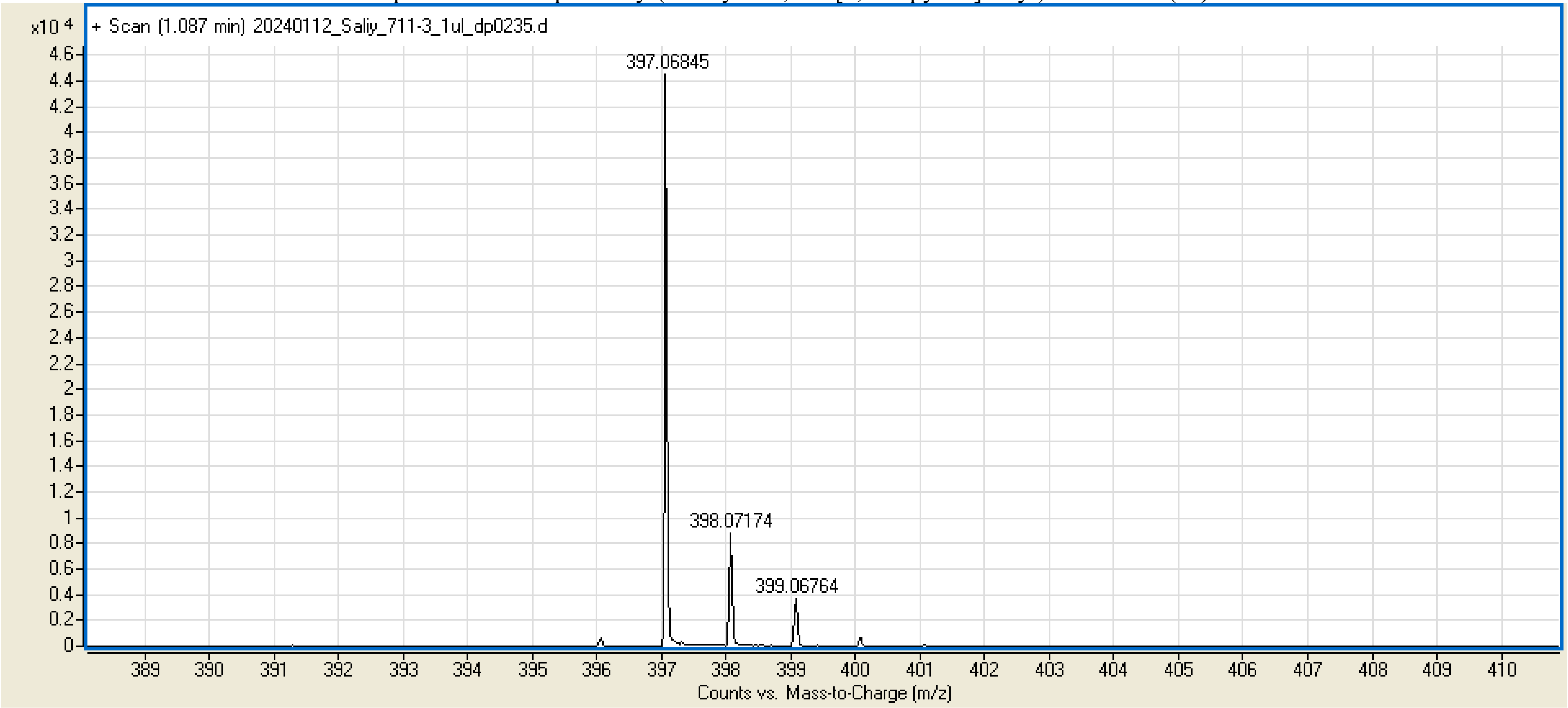

^{1}H NMR spectrum of (4-ethyl-5-propyl-2'-tosyl-1*H*,1'*H*-[2,3'-bipyrrol]-4'-yl)(phenyl)methanone (**2d**) in $CDCl_3$.

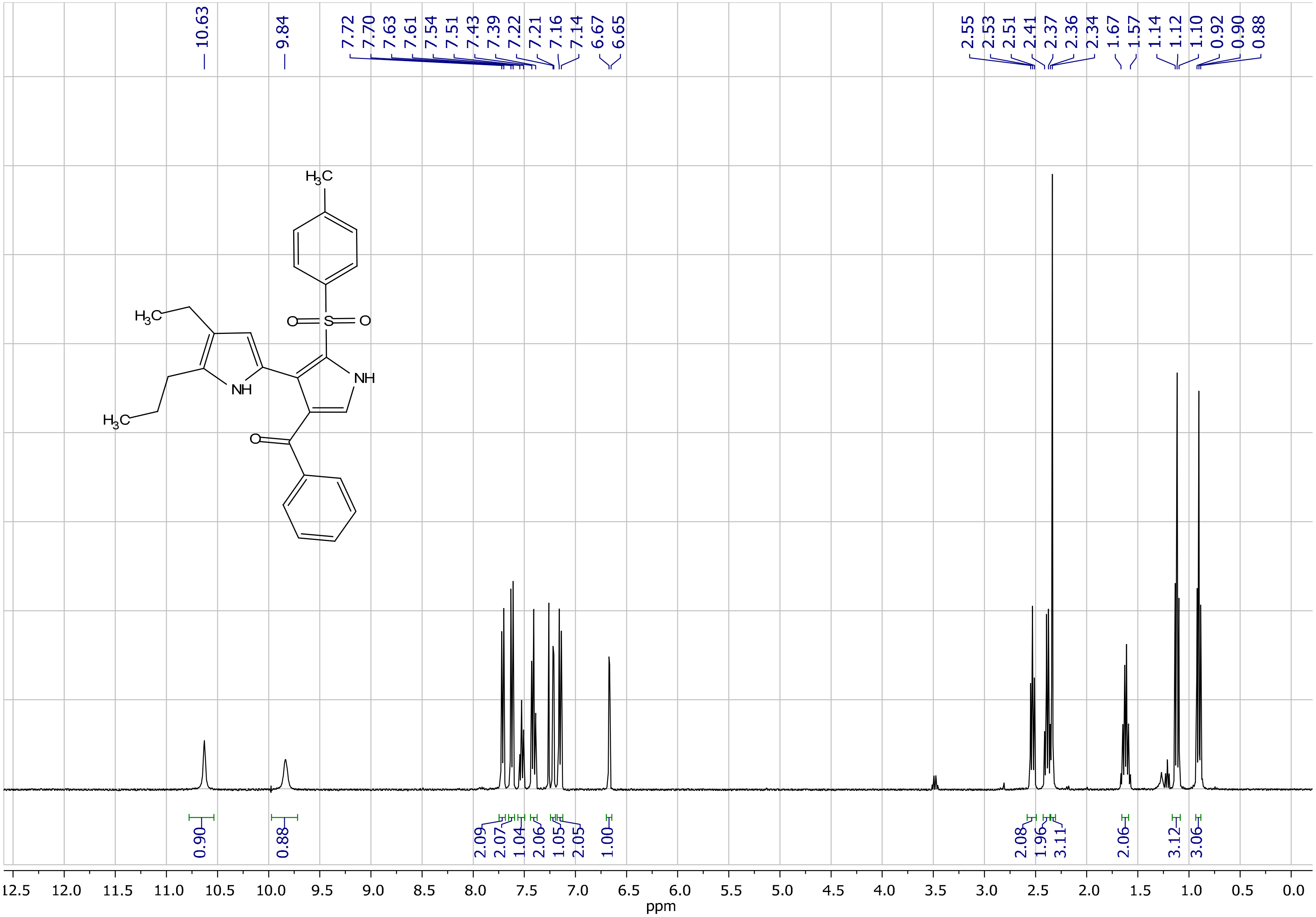

^{13}C NMR spectrum of (4-ethyl-5-propyl-2'-tosyl-1*H*,1'*H*-[2,3'-bipyrrol]-4'-yl)(phenyl)methanone (**2d**) in $CDCl_3$.

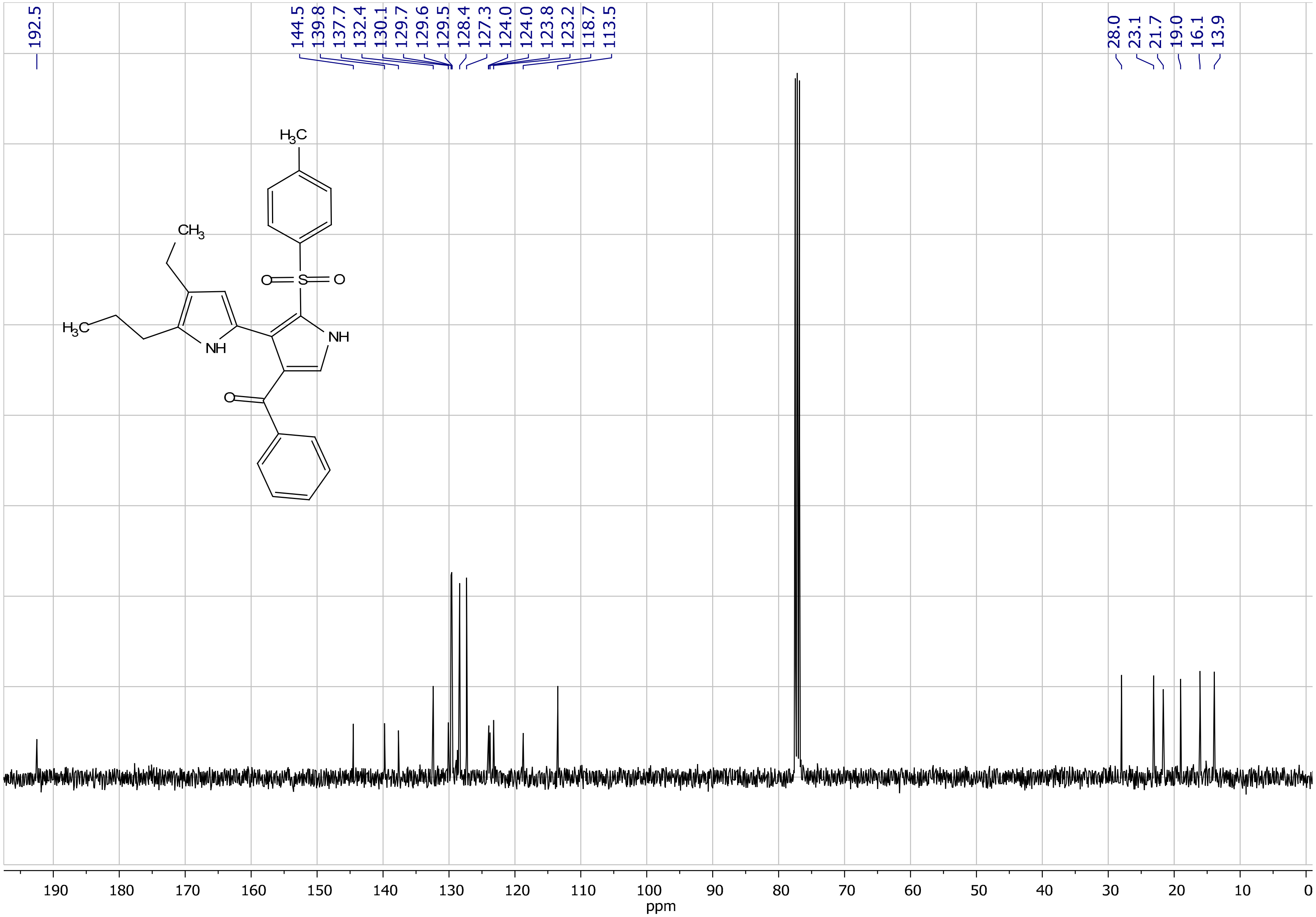

HRMS spectrum of (4-ethyl-5-propyl-2'-tosyl-1*H*,1'*H*-[2,3'-bipyrrol]-4'-yl)(phenyl)methanone (**2d**)

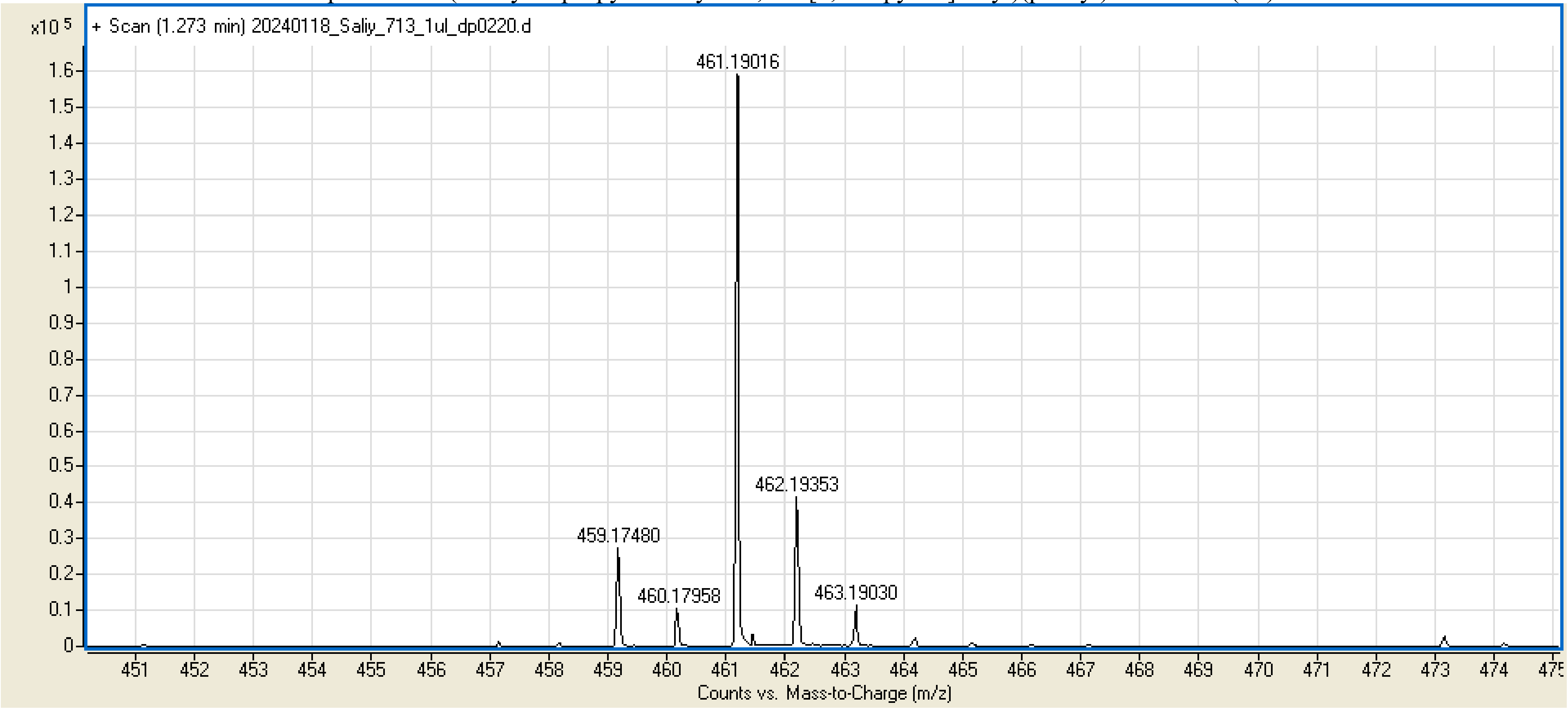

^{1}H NMR spectrum of phenyl(4-(4,5,6,7-tetrahydro-1*H*-indol-2-yl)-5-tosyl-1*H*-pyrrol-3-yl)methanone (**2e**) in $CDCl_3$.

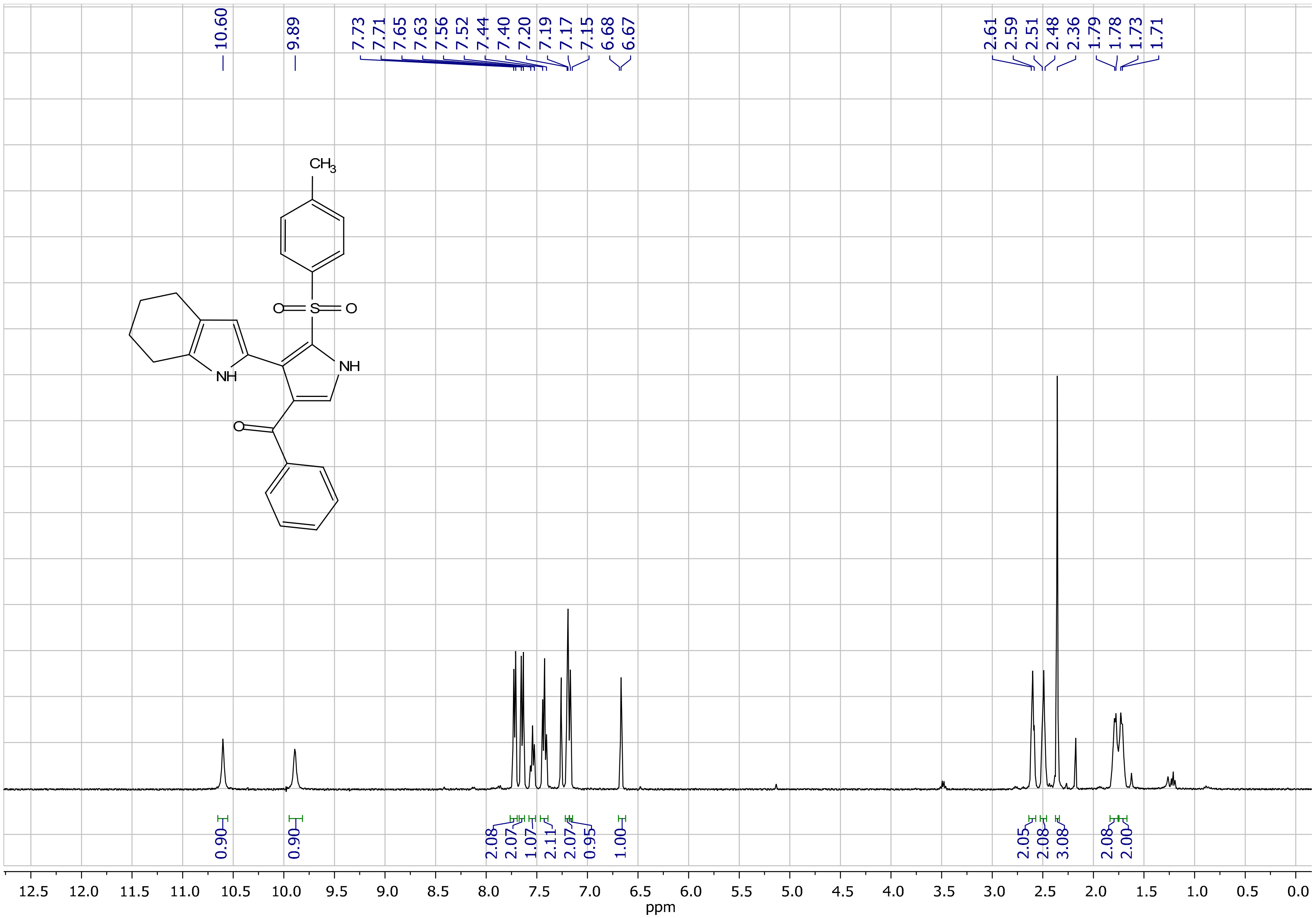

^{13}C NMR spectrum of phenyl(4-(4,5,6,7-tetrahydro-1*H*-indol-2-yl)-5-tosyl-1*H*-pyrrol-3-yl)methanone (**2e**) in $CDCl_3$.

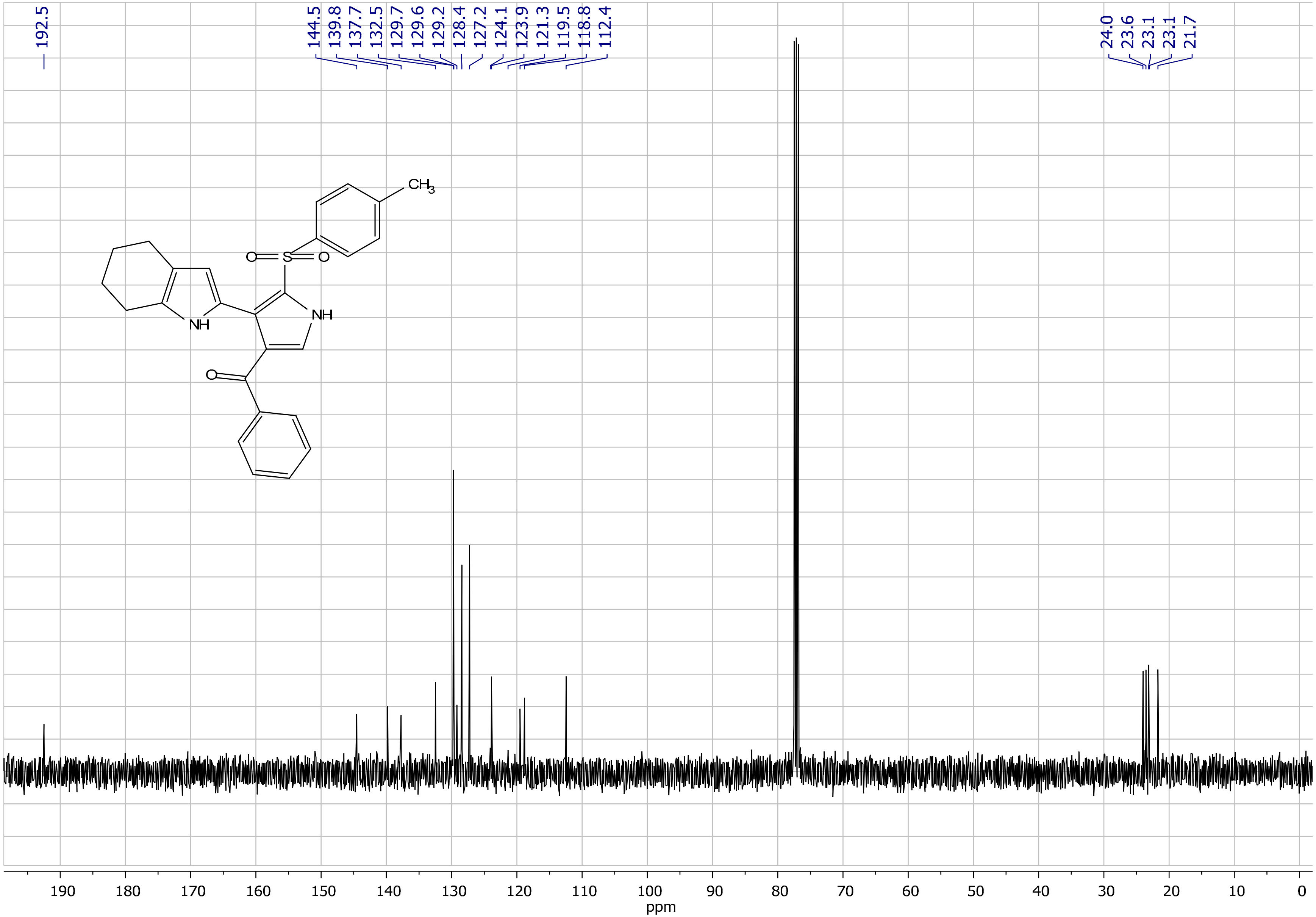

HRMS spectrum of phenyl(4-(4,5,6,7-tetrahydro-1*H*-indol-2-yl)-5-tosyl-1*H*-pyrrol-3-yl)methanone (**2e**)

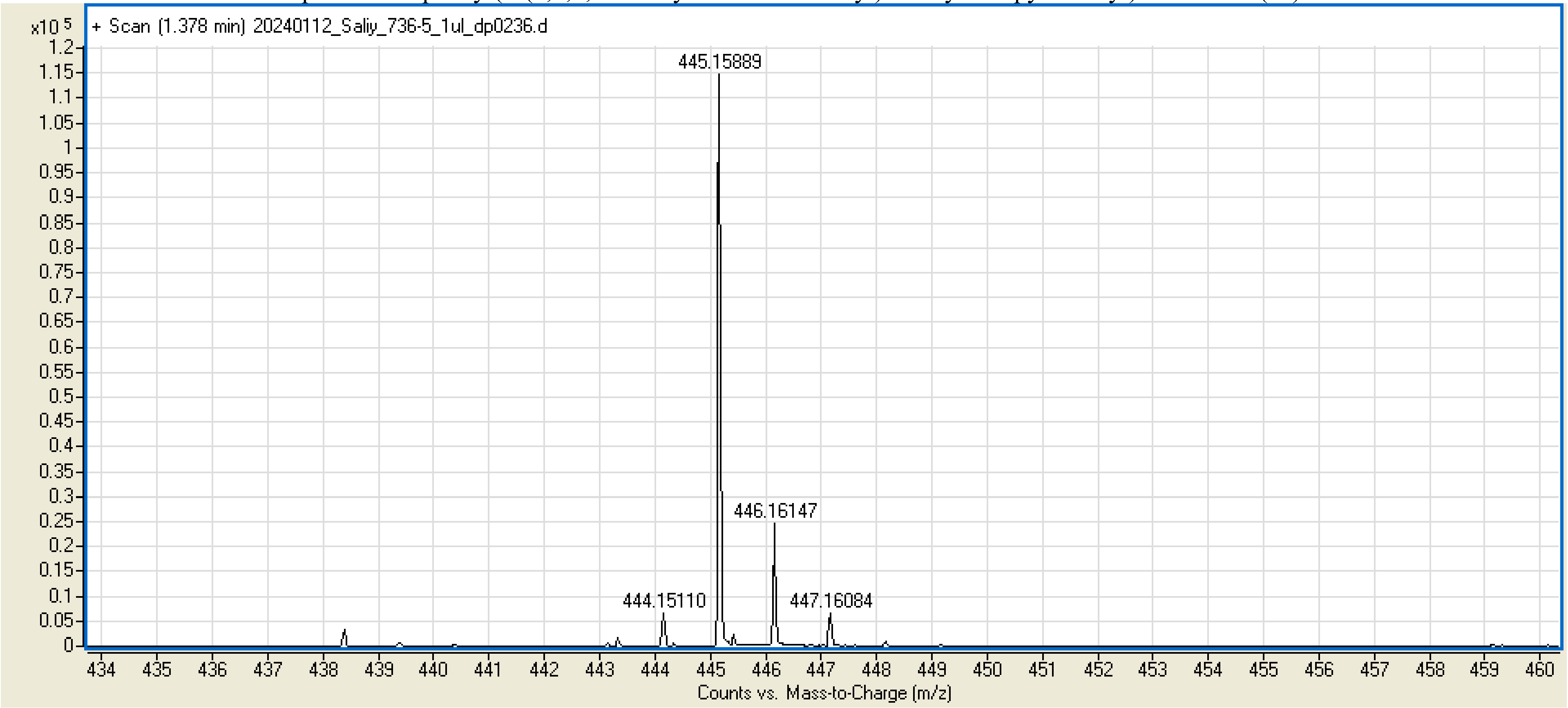

^{1}H NMR spectrum of furan-2-yl(4-(4,5,6,7-tetrahydro-1*H*-indol-2-yl)-5-tosyl-1*H*-pyrrol-3-yl)methanone (**2f**) in $CDCl_3$.

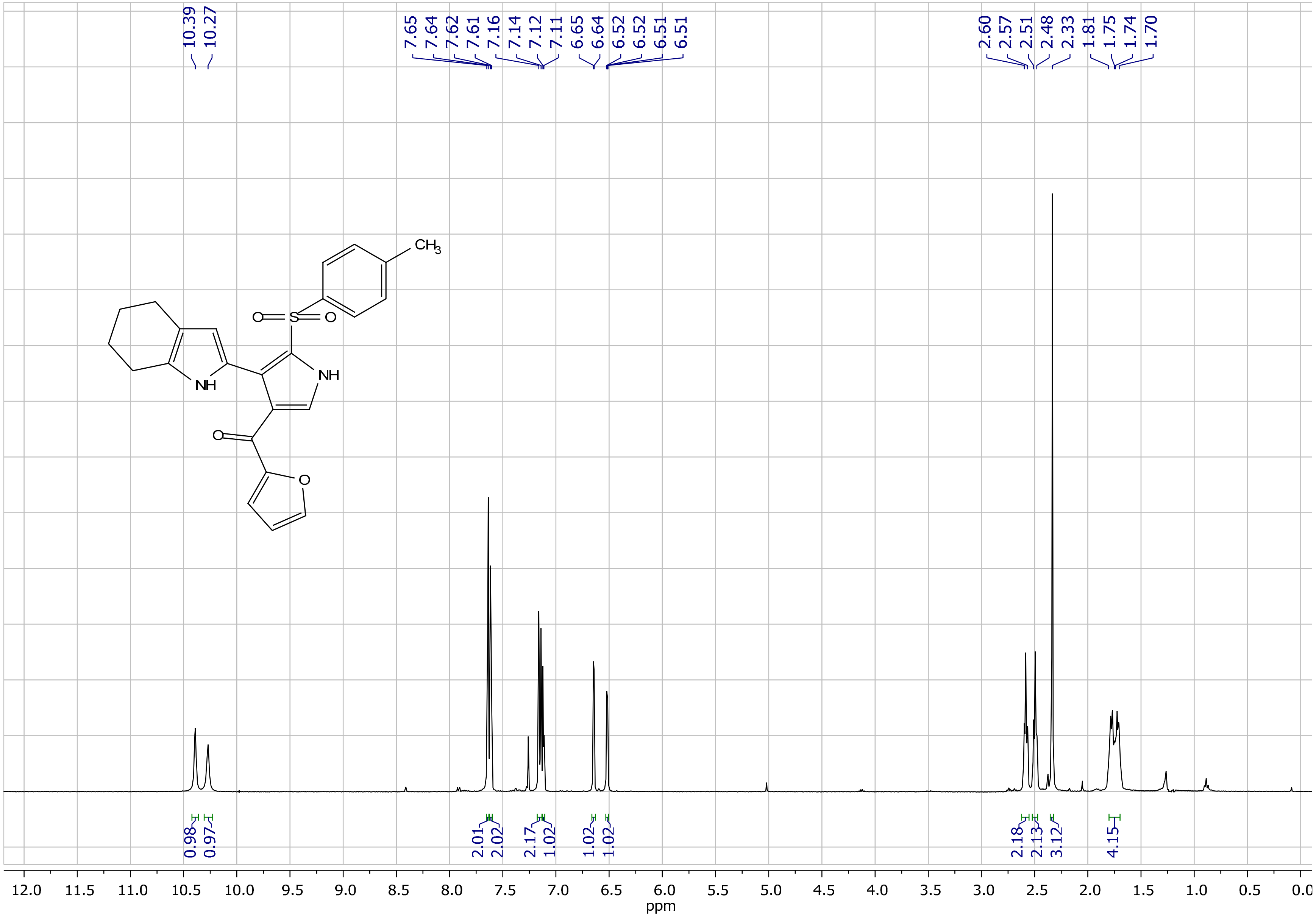

^{13}C NMR spectrum of furan-2-yl(4-(4,5,6,7-tetrahydro-1*H*-indol-2-yl)-5-tosyl-1*H*-pyrrol-3-yl)methanone (**2f**) in $CDCl_3$.

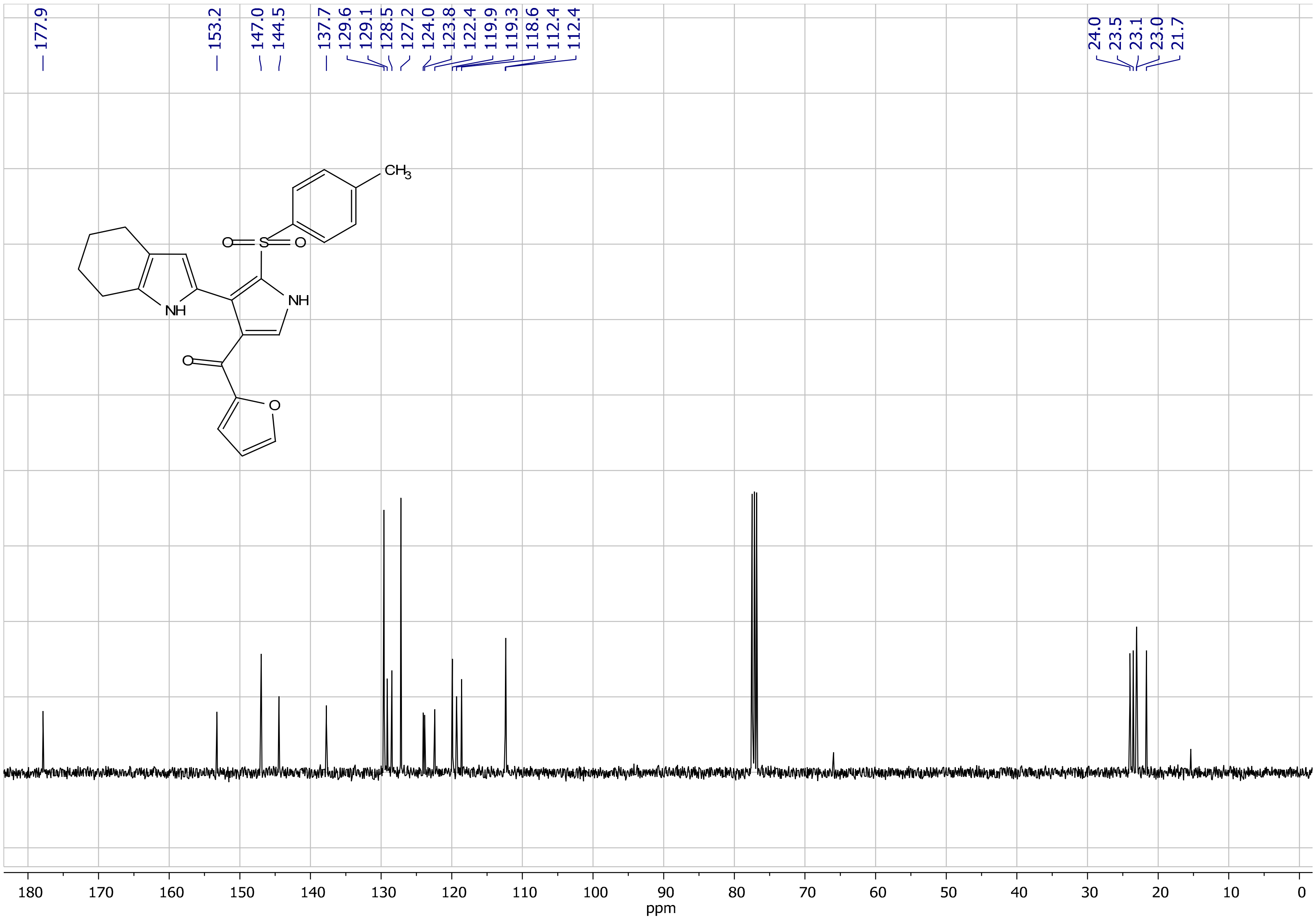

HRMS spectrum of furan-2-yl(4-(4,5,6,7-tetrahydro-1*H*-indol-2-yl)-5-tosyl-1*H*-pyrrol-3-yl)methanone (**2f**)

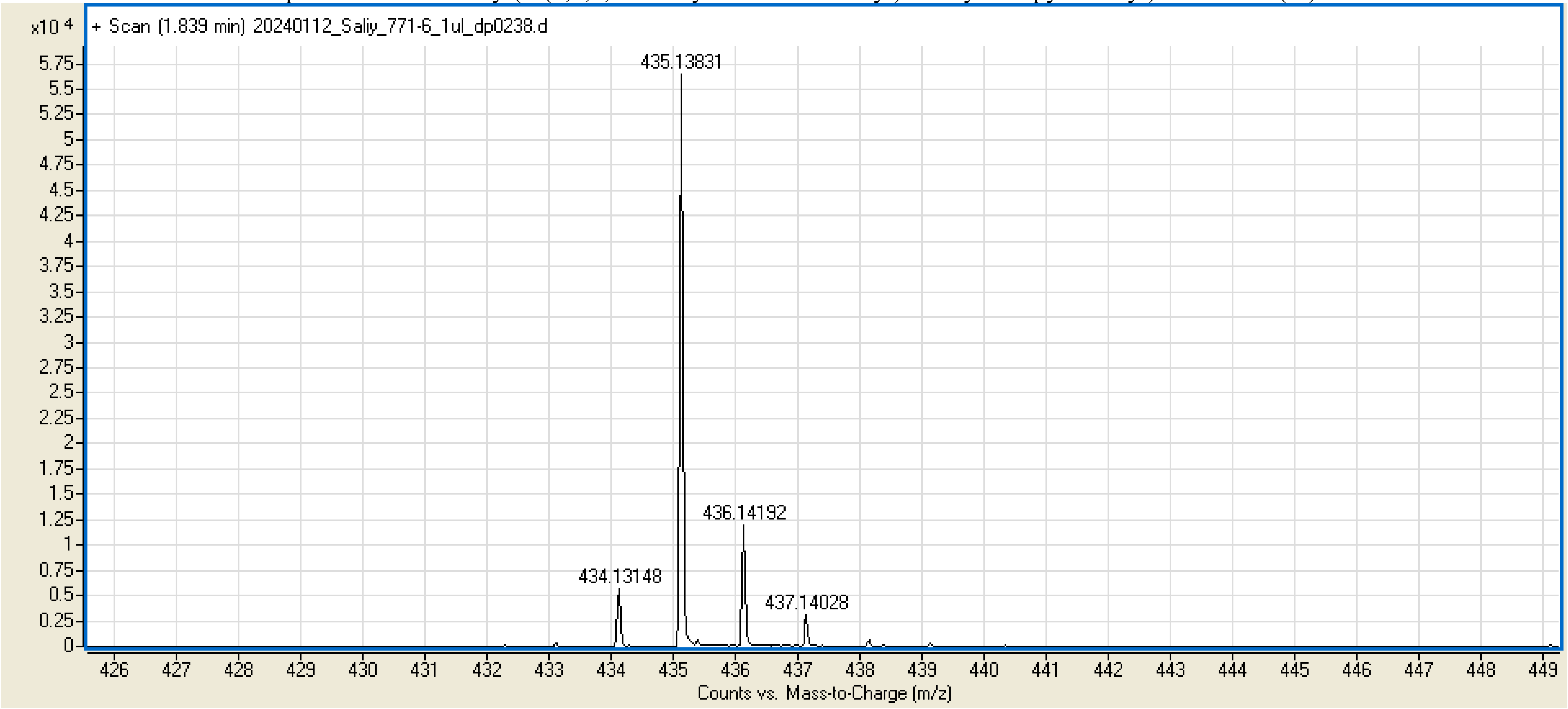

^{1}H NMR spectrum of (4-(4,5,6,7-tetrahydro-1*H*-indol-2-yl)-5-tosyl-1*H*-pyrrol-3-yl)(thiophen-2-yl)methanone (**2g**) in $CDCl_3$.

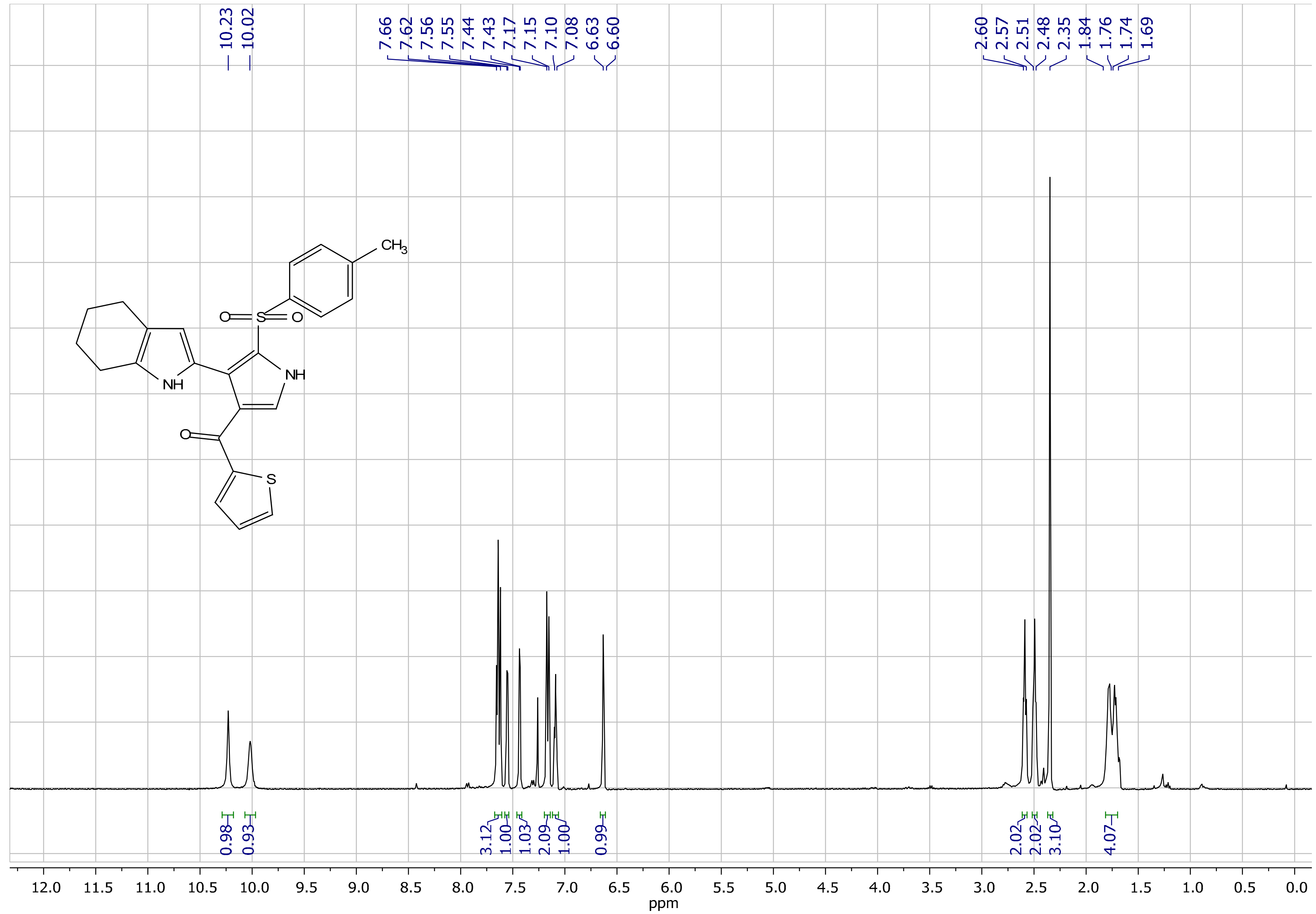

^{13}C NMR spectrum of (4-(4,5,6,7-tetrahydro-1*H*-indol-2-yl)-5-tosyl-1*H*-pyrrol-3-yl)(thiophen-2-yl)methanone (**2g**) in $CDCl_3$.

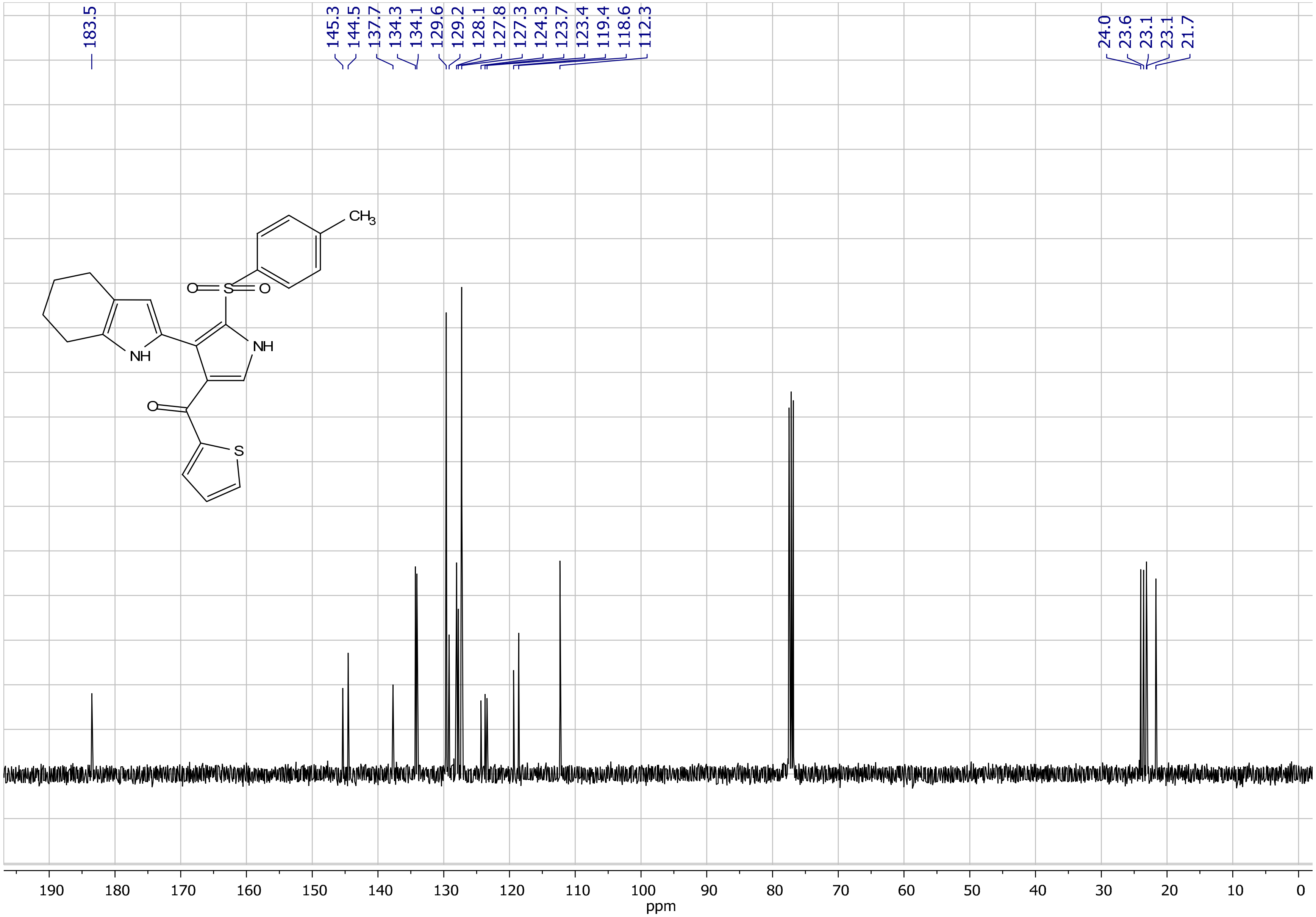

HRMS spectrum of (4-(4,5,6,7-tetrahydro-1*H*-indol-2-yl)-5-tosyl-1*H*-pyrrol-3-yl)(thiophen-2-yl)methanone (**2g**)

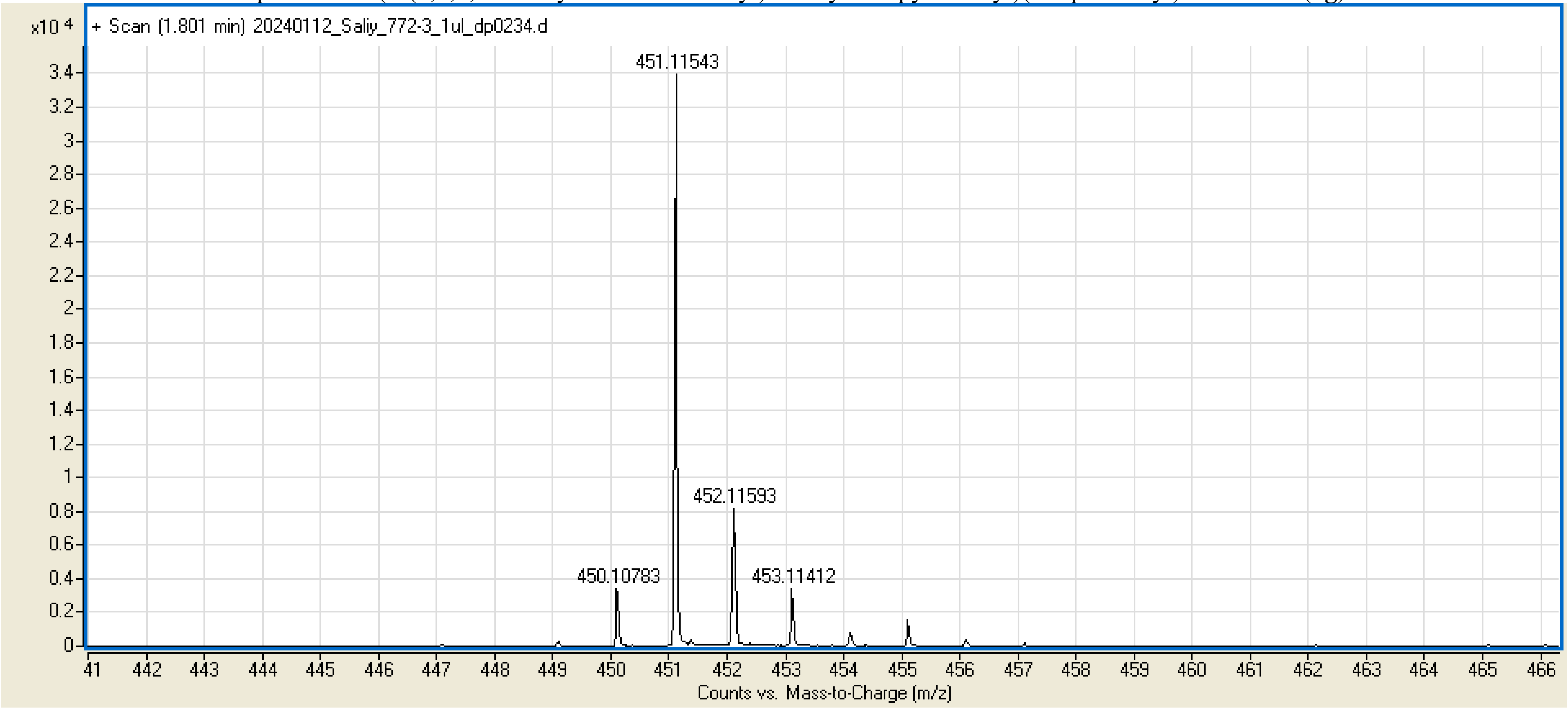

^{1}H NMR spectrum of phenyl(5-phenyl-2'-tosyl-1*H*,1'*H*-[2,3'-bipyrrol]-4'-yl)methanone (**2h**) in $CDCl_3$.

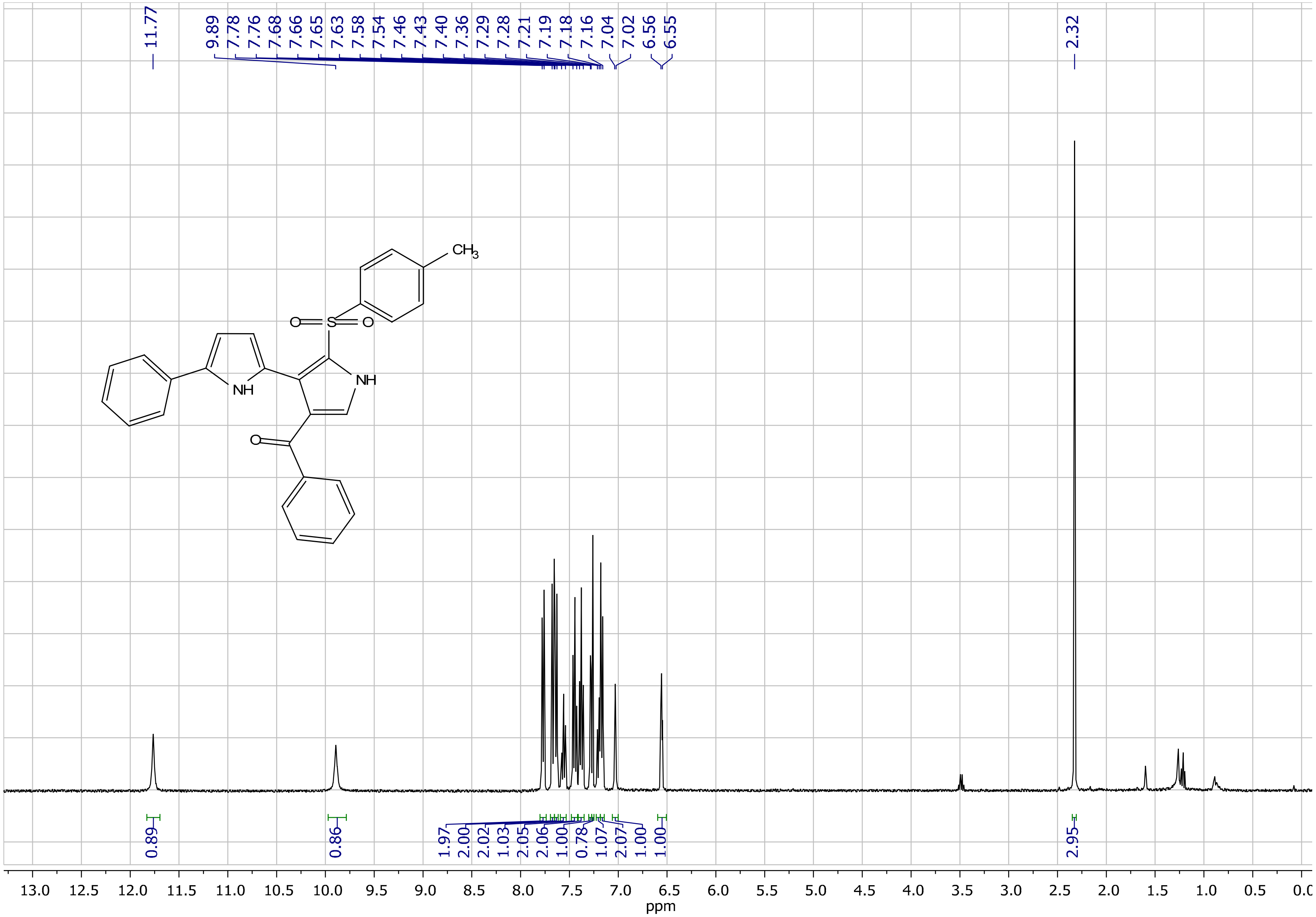

^{13}C NMR spectrum of phenyl(5-phenyl-2'-tosyl-1*H*,1'*H*-[2,3'-bipyrrol]-4'-yl)methanone (**2h**) in $CDCl_3$.

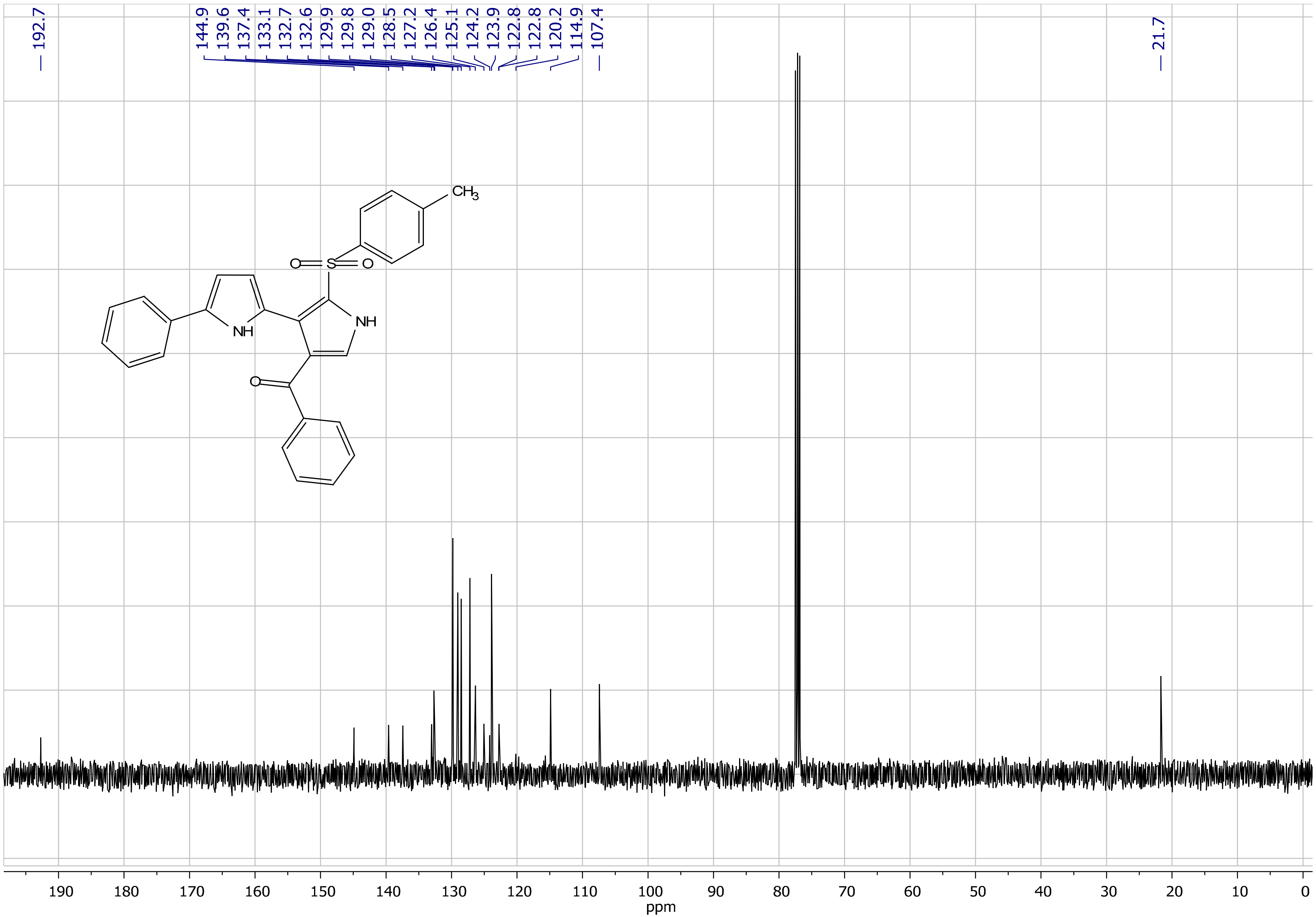

HRMS spectrum of phenyl(5-phenyl-2'-tosyl-1*H*,1'*H*-[2,3'-bipyrrol]-4'-yl)methanone (**2h**)

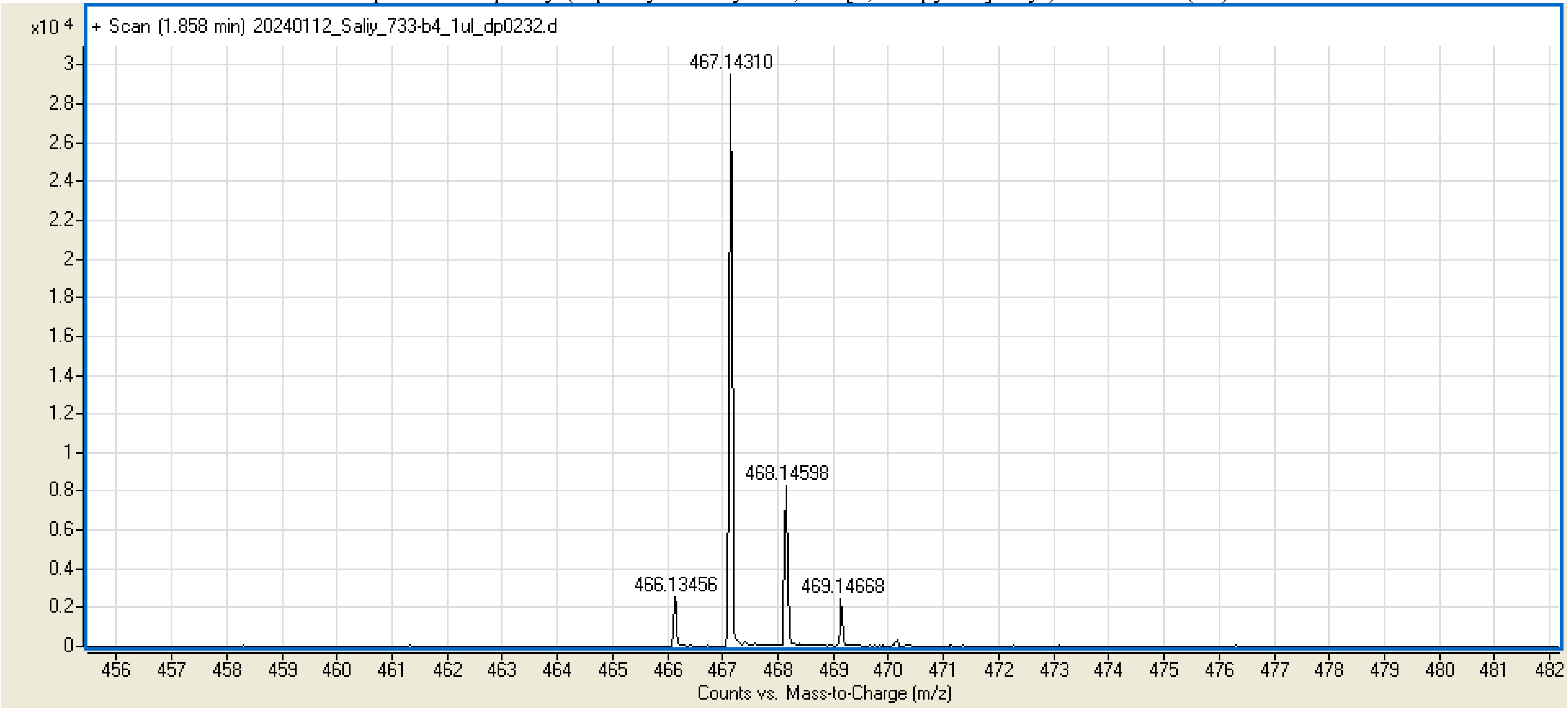

^{1}H NMR spectrum of furan-2-yl(5-phenyl-2'-tosyl-1*H*,1'*H*-[2,3'-bipyrrol]-4'-yl)methanone (**2i**) in $CDCl_3$.

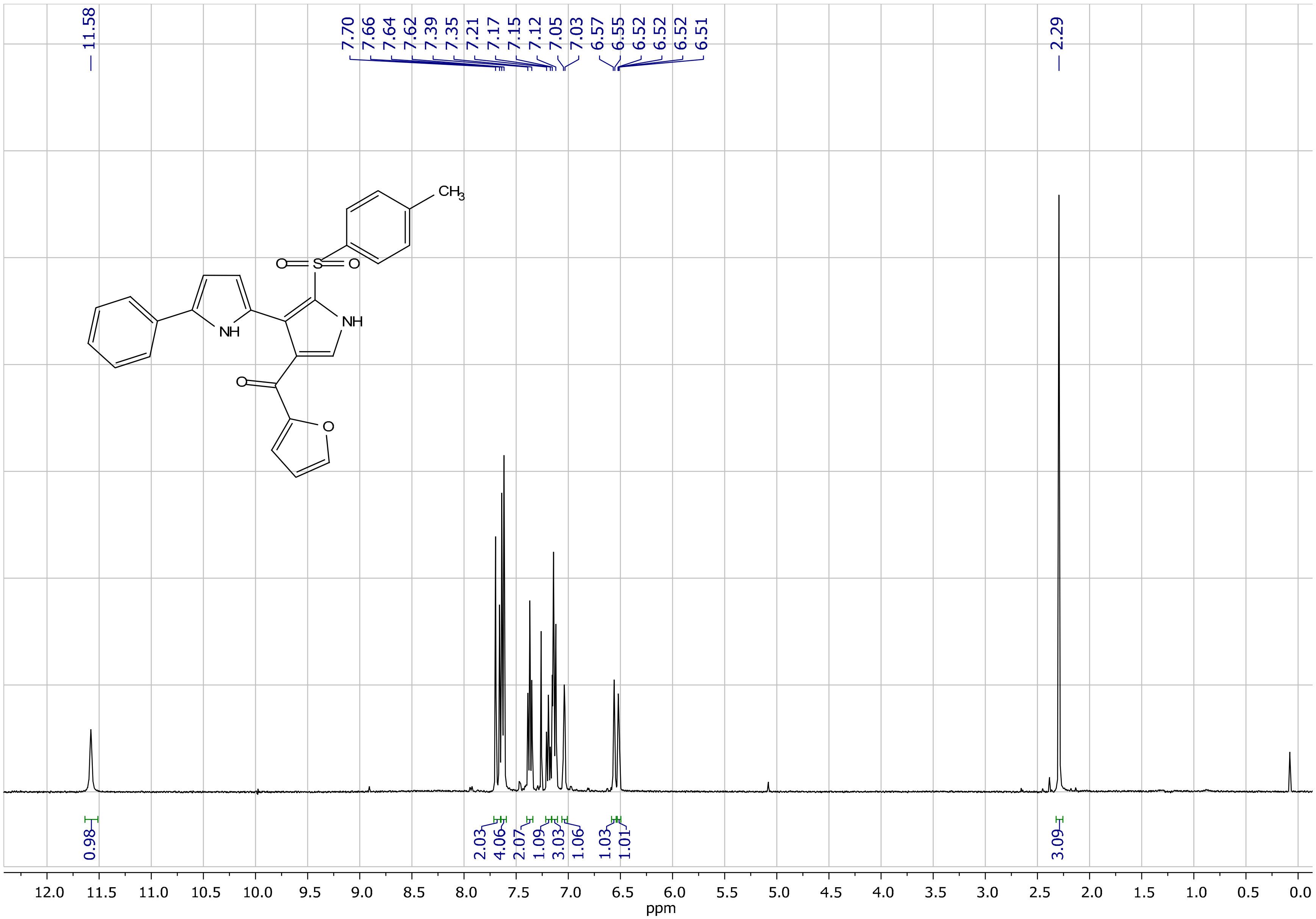

^{1}C NMR spectrum of furan-2-yl(5-phenyl-2'-tosyl-1*H*,1'*H*-[2,3'-bipyrrol]-4'-yl)methanone (**2i**) in $CDCl_3$.

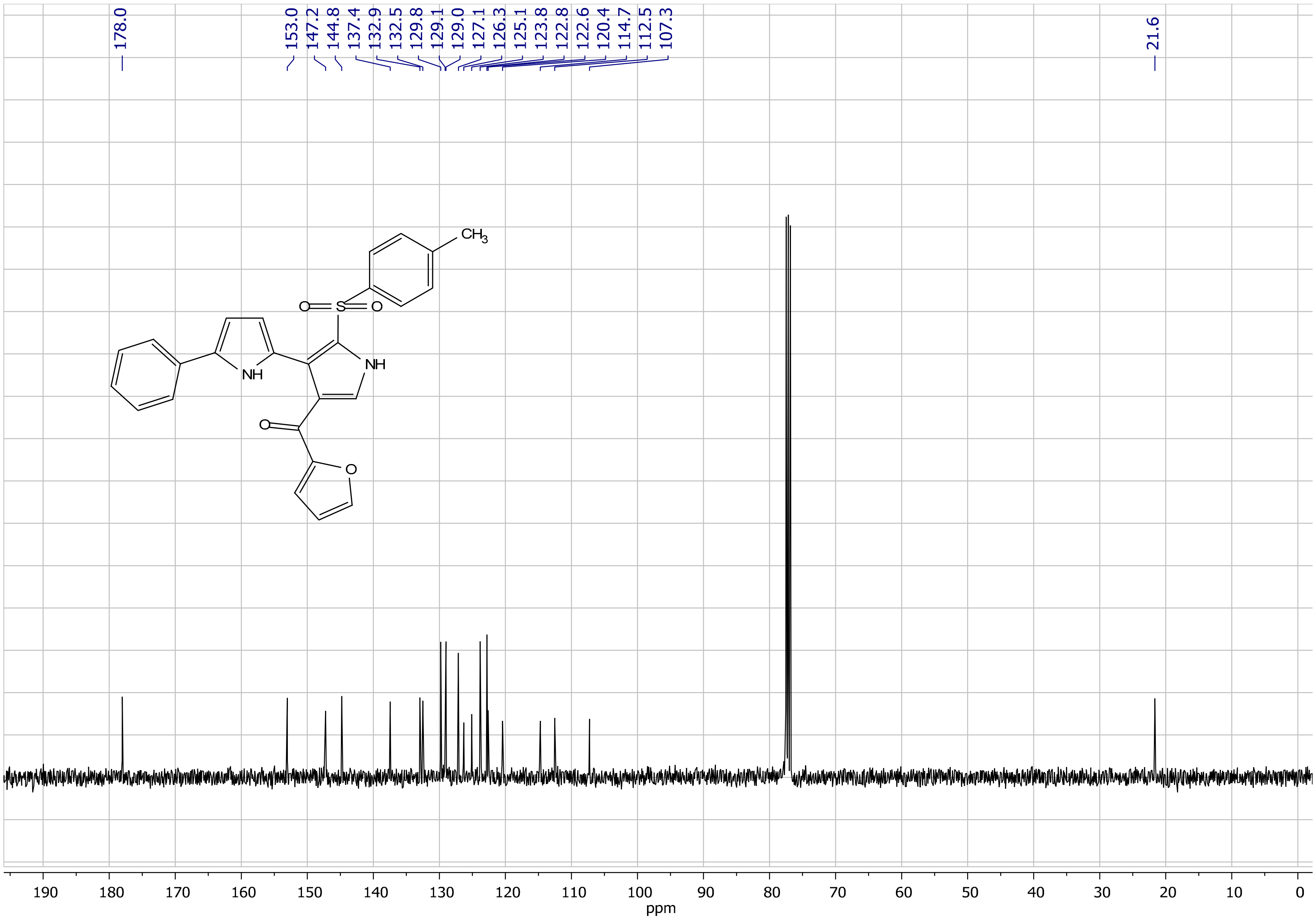

HRMS spectrum of furan-2-yl(5-phenyl-2'-tosyl-1*H*,1'*H*-[2,3'-bipyrrol]-4'-yl)methanone (**2i**)

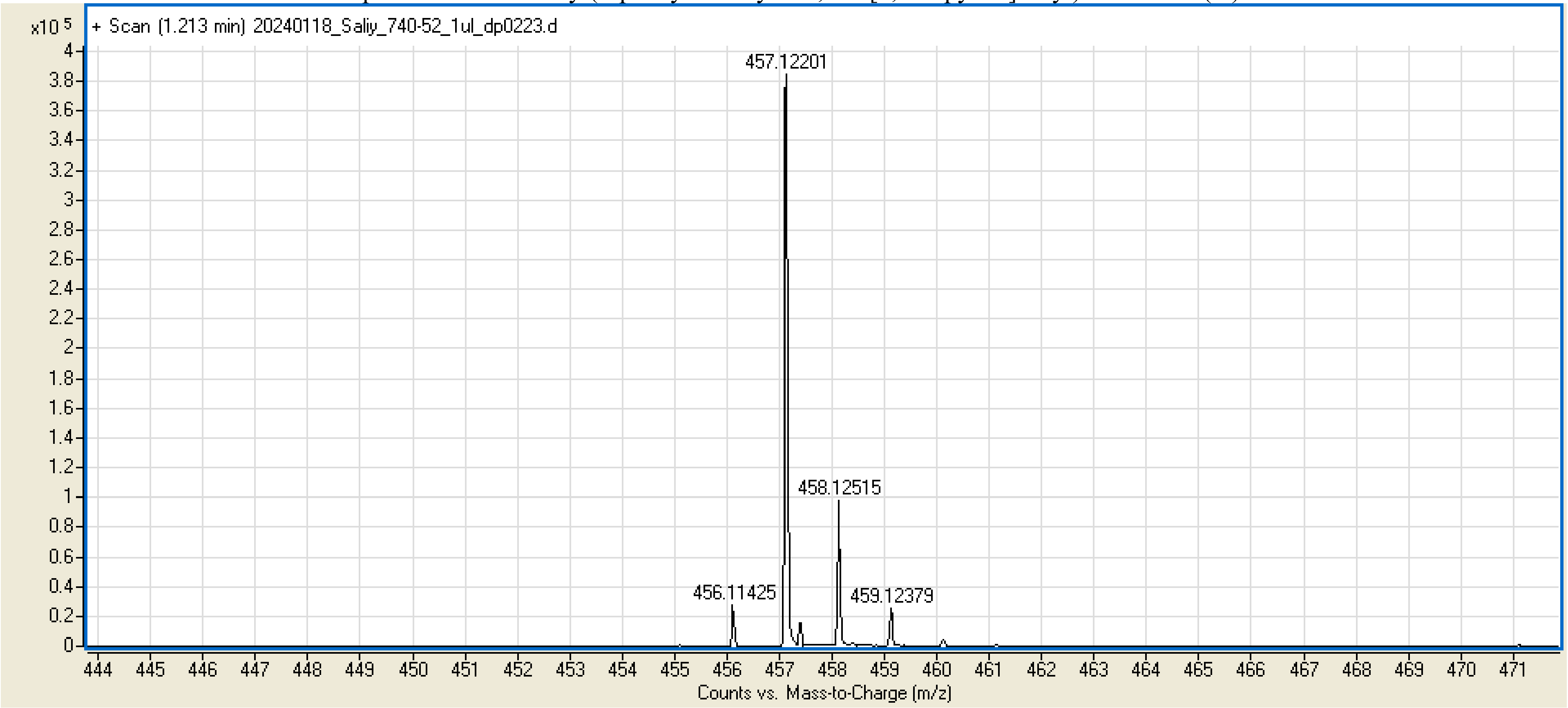

^{1}H NMR spectrum of (5-phenyl-2'-tosyl-1*H*,1'*H*-[2,3'-bipyrrol]-4'-yl)(thiophen-2-yl)methanone (**2j**) in $CDCl_3$.

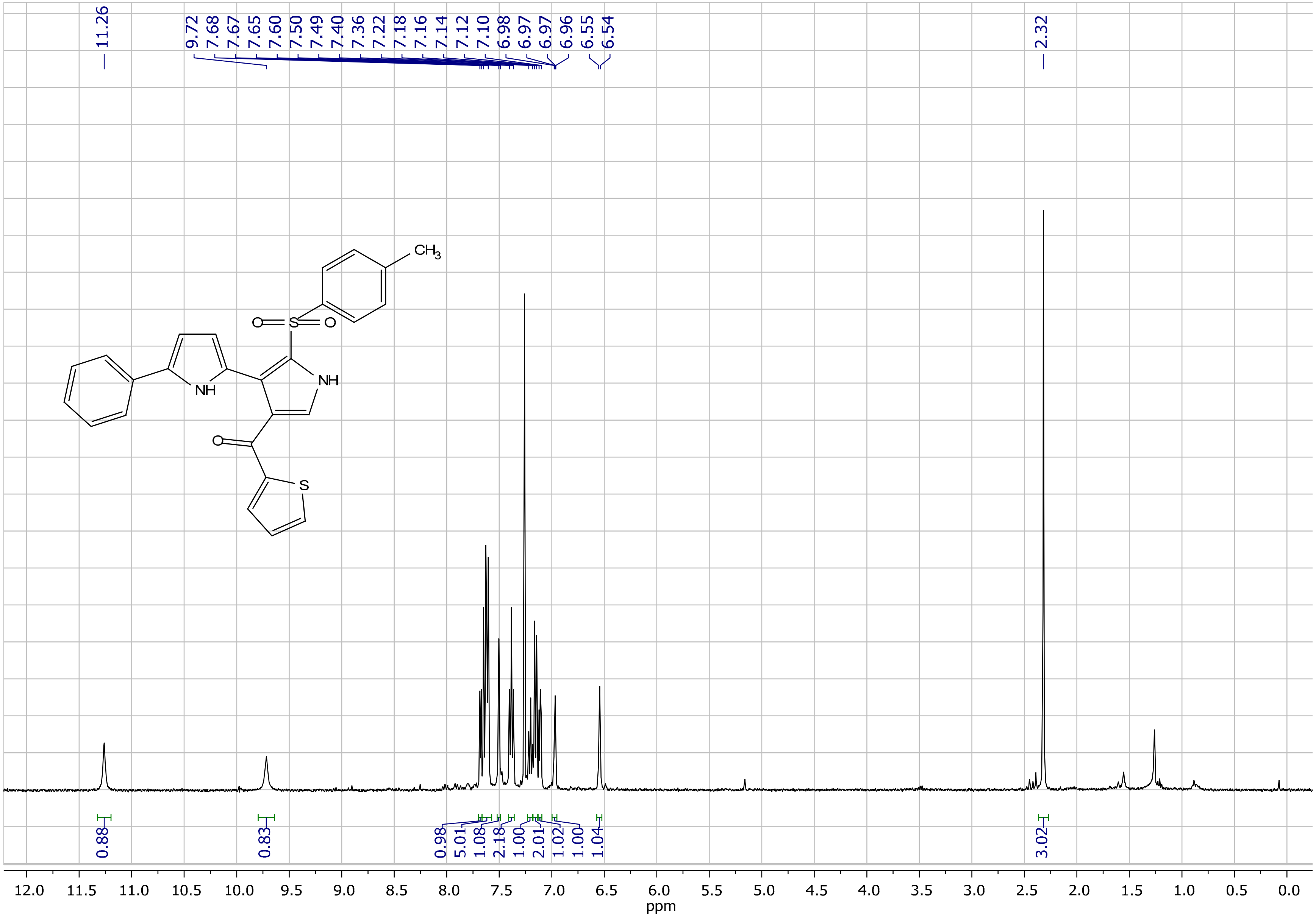

^{13}C NMR spectrum of (5-phenyl-2'-tosyl-1*H*,1'*H*-[2,3'-bipyrrol]-4'-yl)(thiophen-2-yl)methanone (**2j**) in $CDCl_3$.

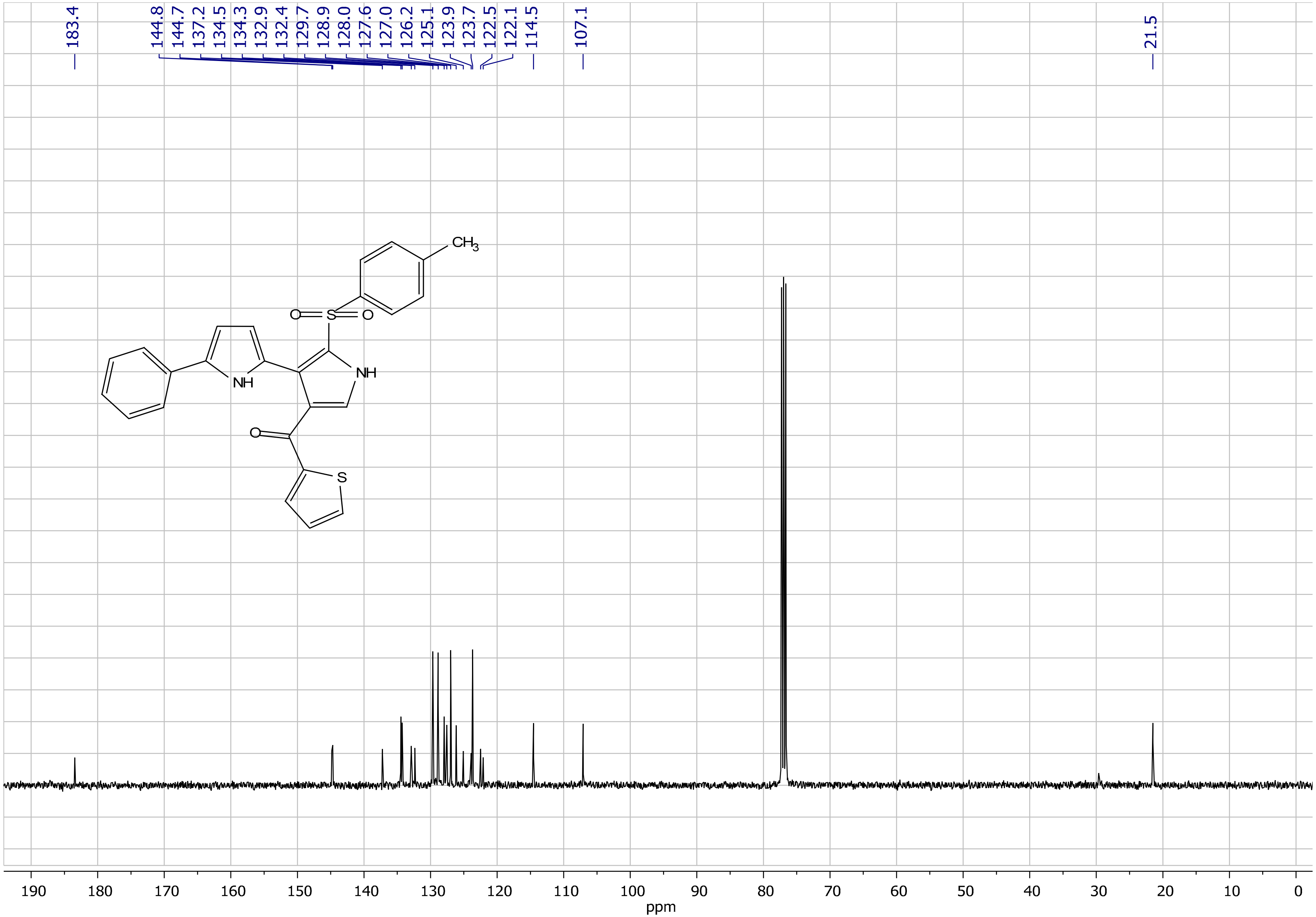

HRMS spectrum of (5-phenyl-2'-tosyl-1*H*,1'*H*-[2,3'-bipyrrol]-4'-yl)(thiophen-2-yl)methanone (**2j**)

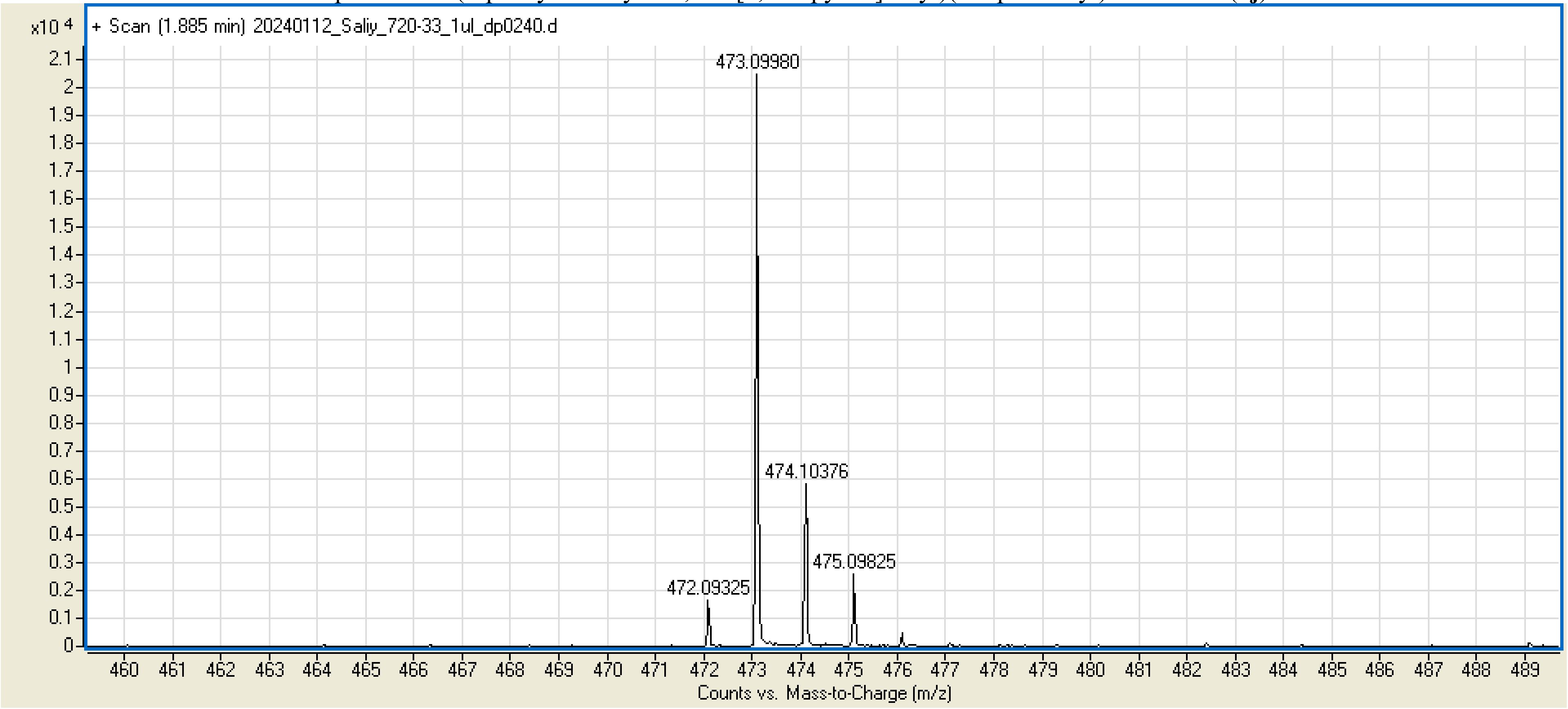

^{1}H NMR spectrum of (5-(4-(dimethylamino)phenyl)-2'-tosyl-1H,1'H-[2,3'-bipyrrol]-4'-yl)(phenyl)methanone (**2k**) in $CDCl_3$.

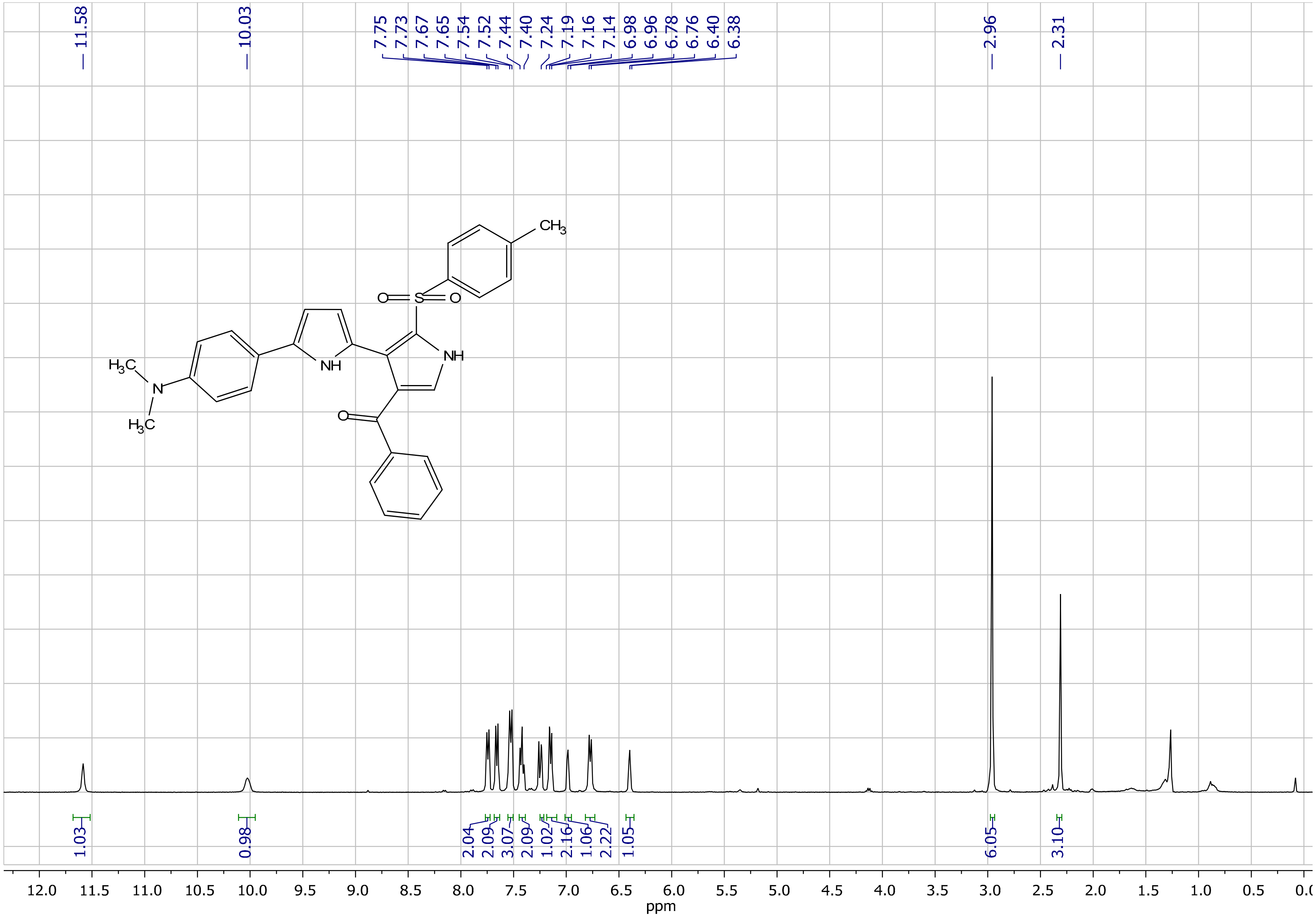

^{13}C NMR spectrum of (5-(4-(dimethylamino)phenyl)-2'-tosyl-1H,1'H-[2,3'-bipyrrol]-4'-yl)(phenyl)methanone (**2k**) in $CDCl_3$.

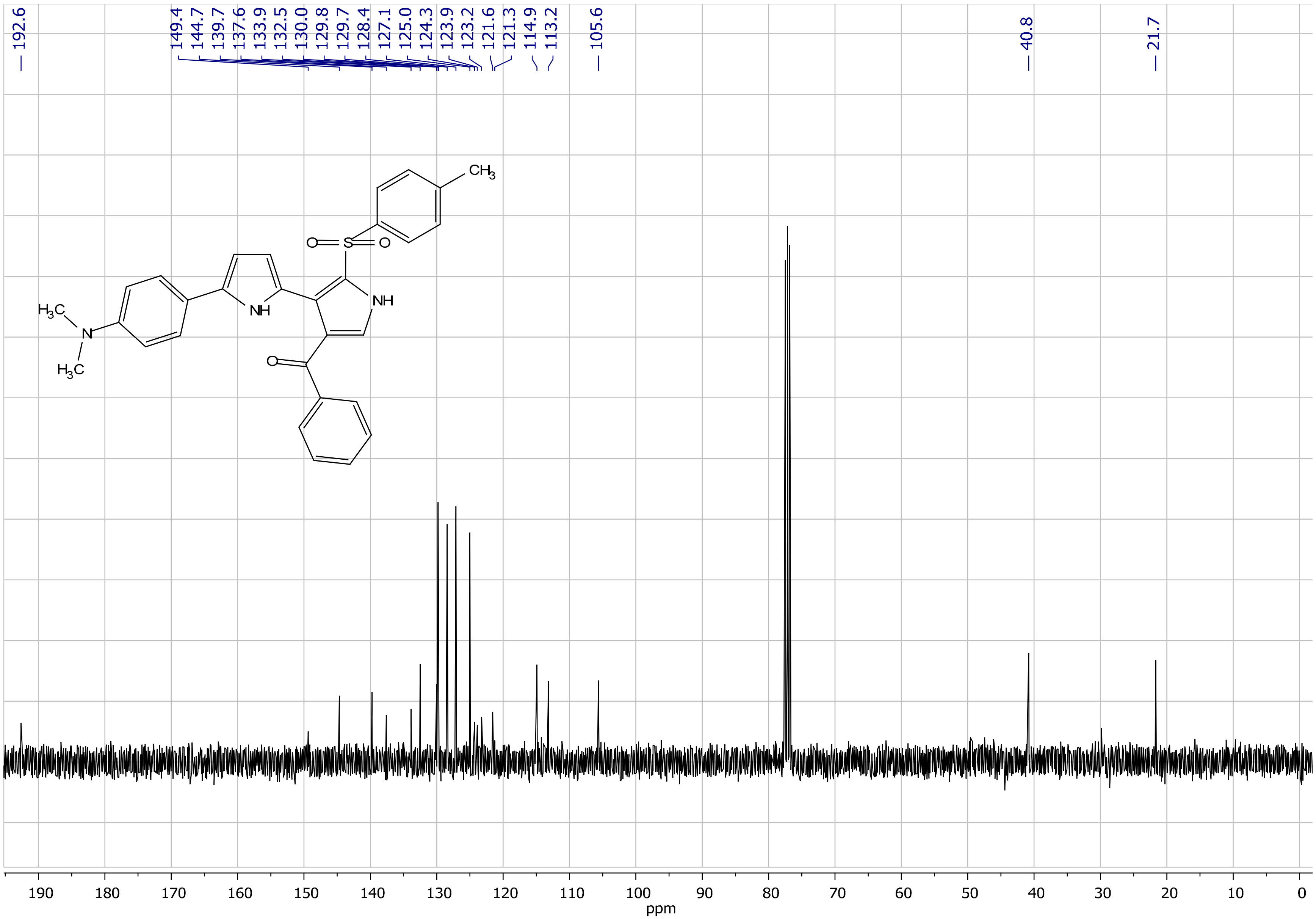

HRMS spectrum of (5-(4-(dimethylamino)phenyl)-2'-tosyl-1H,1'H-[2,3'-bipyrrol]-4'-yl)(phenyl)methanone (**2k**)

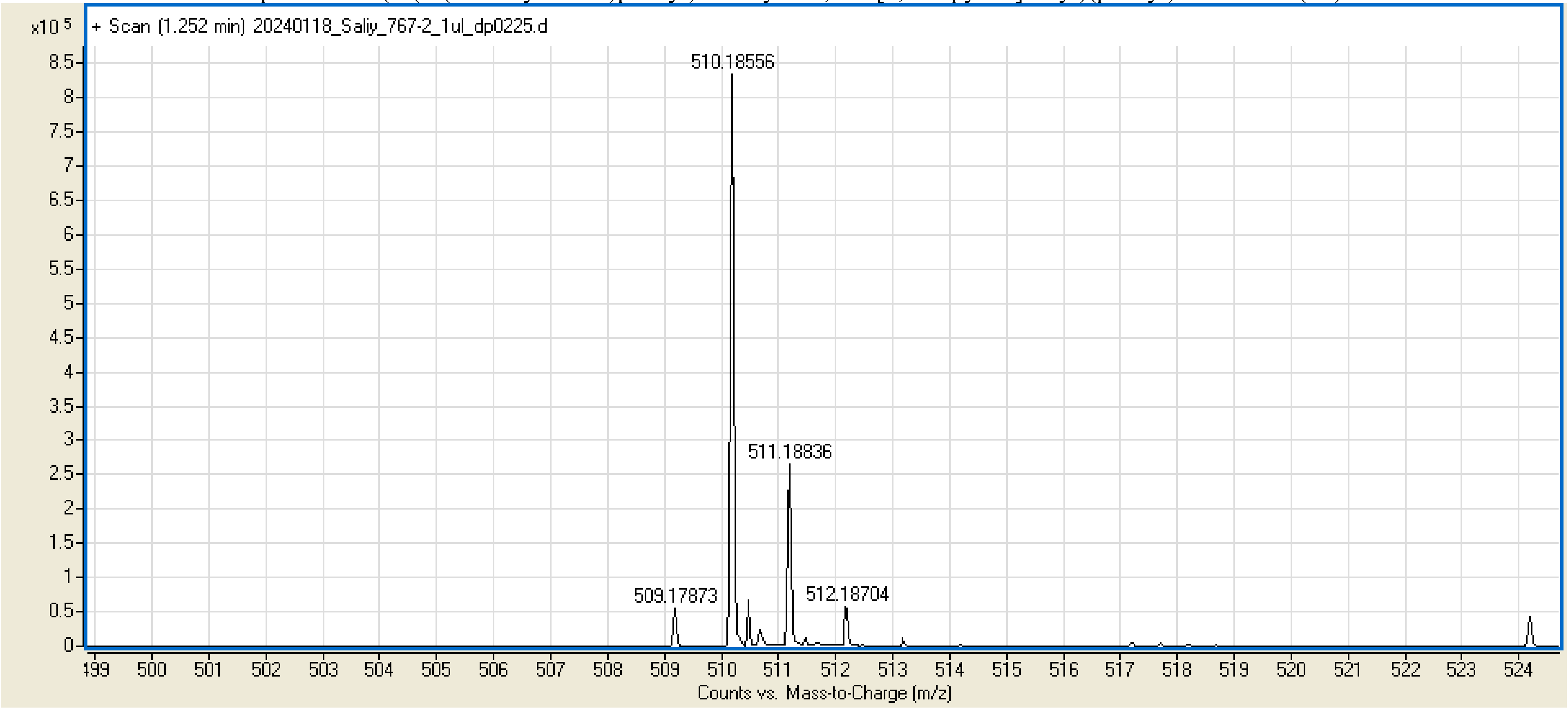

^{1}H NMR spectrum of (5-(4-fluorophenyl)-2'-tosyl-1*H*,1'*H*-[2,3'-bipyrrol]-4'-yl)(phenyl)methanone (**2l**) in $CDCl_3$.

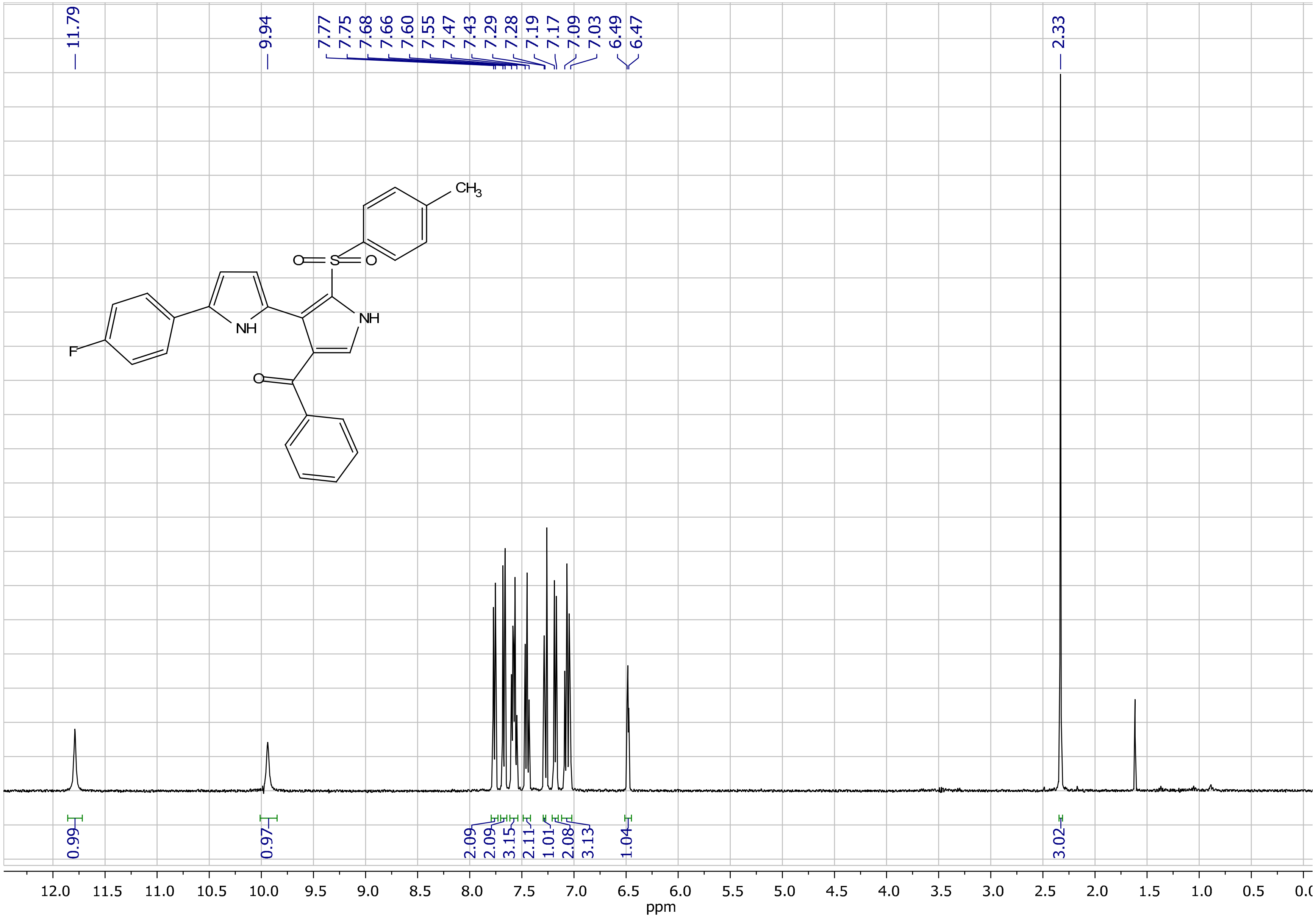

^{13}C NMR spectrum of (5-(4-fluorophenyl)-2'-tosyl-1*H*,1'*H*-[2,3'-bipyrrol]-4'-yl)(phenyl)methanone (**2l**) in DMSO-d6.

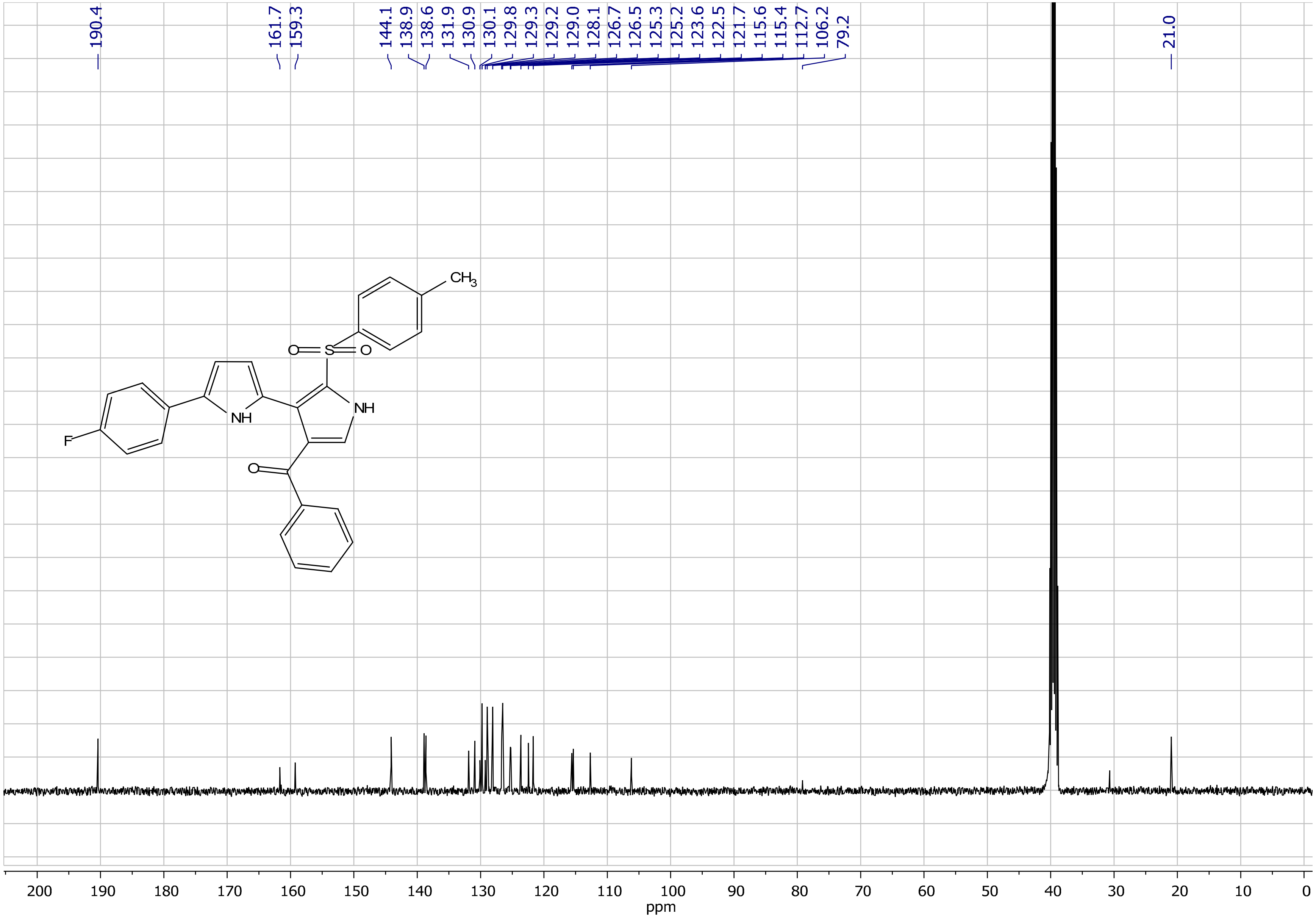

^{1}H NMR spectrum of (5-(4-fluorophenyl)-2'-tosyl-1*H*,1'*H*-[2,3'-bipyrrol]-4'-yl)(furan-2-yl)methanone (**2m**) in $CDCl_3$.

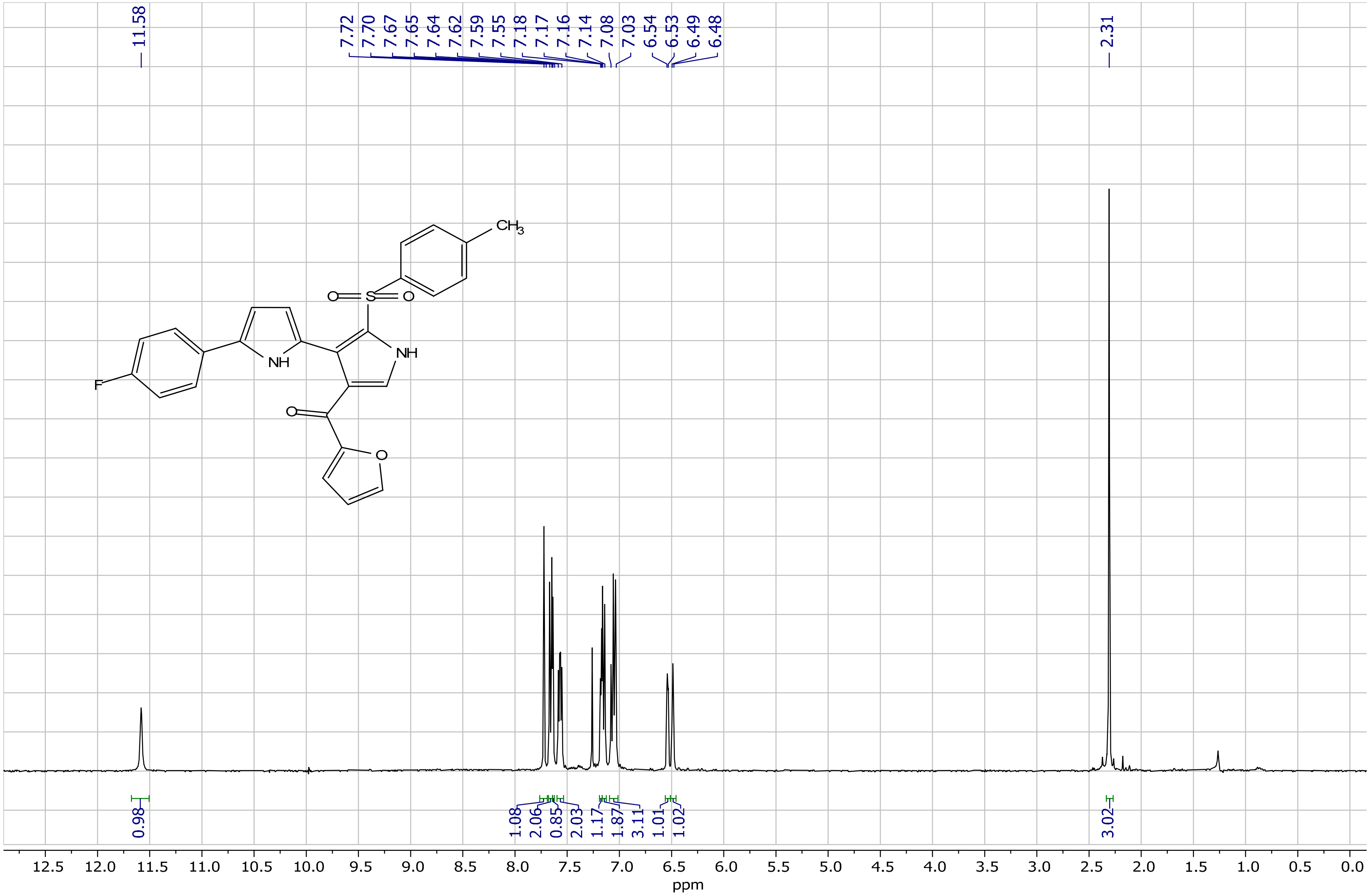

^{13}C NMR spectrum of (5-(4-fluorophenyl)-2'-tosyl-1*H*,1'*H*-[2,3'-bipyrrol]-4'-yl)(furan-2-yl)methanone (**2m**) in $CDCl_3$.

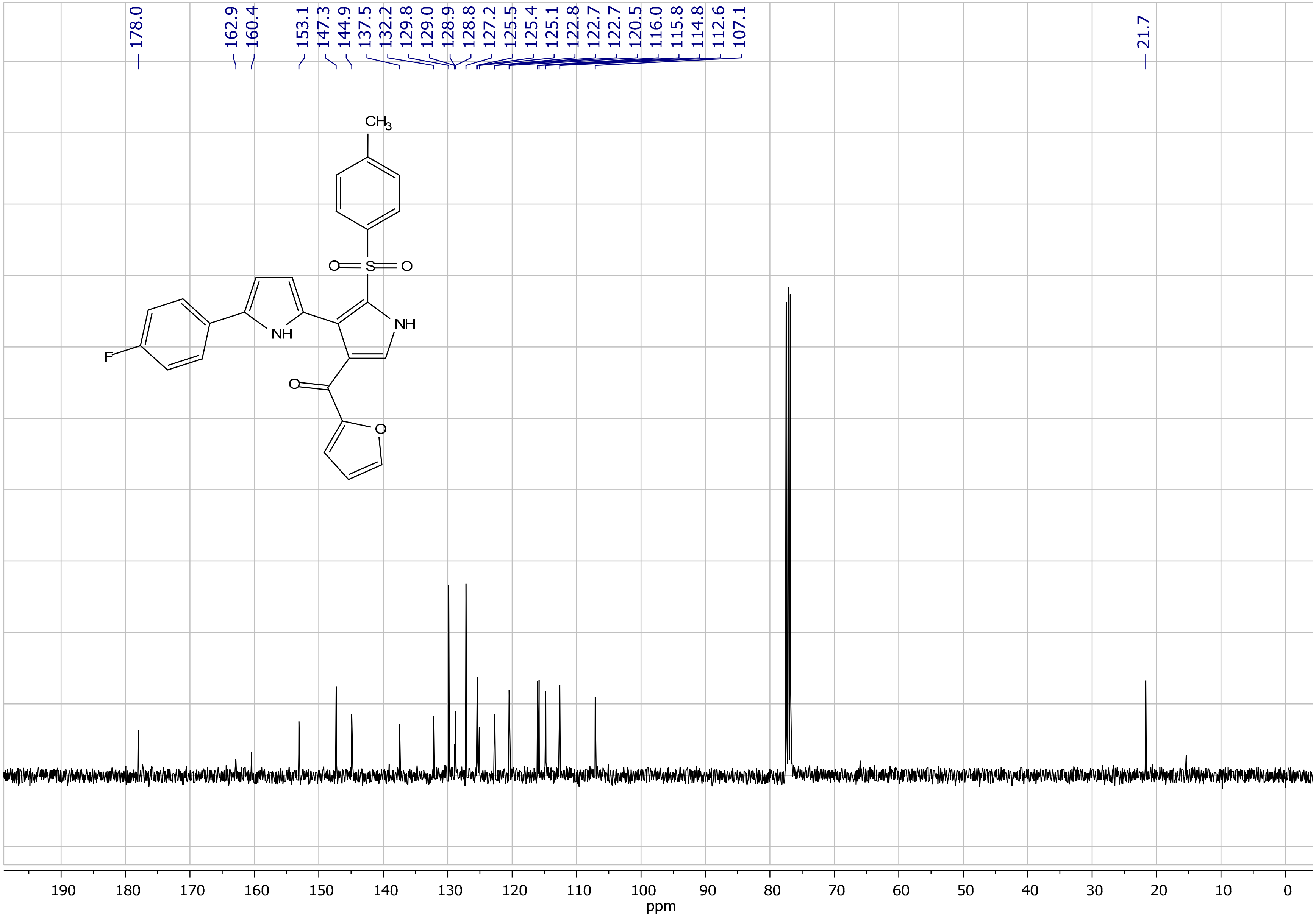

HRMS spectrum of (5-(4-fluorophenyl)-2'-tosyl-1*H*,1'*H*-[2,3'-bipyrrol]-4'-yl)(furan-2-yl)methanone (**2m**)

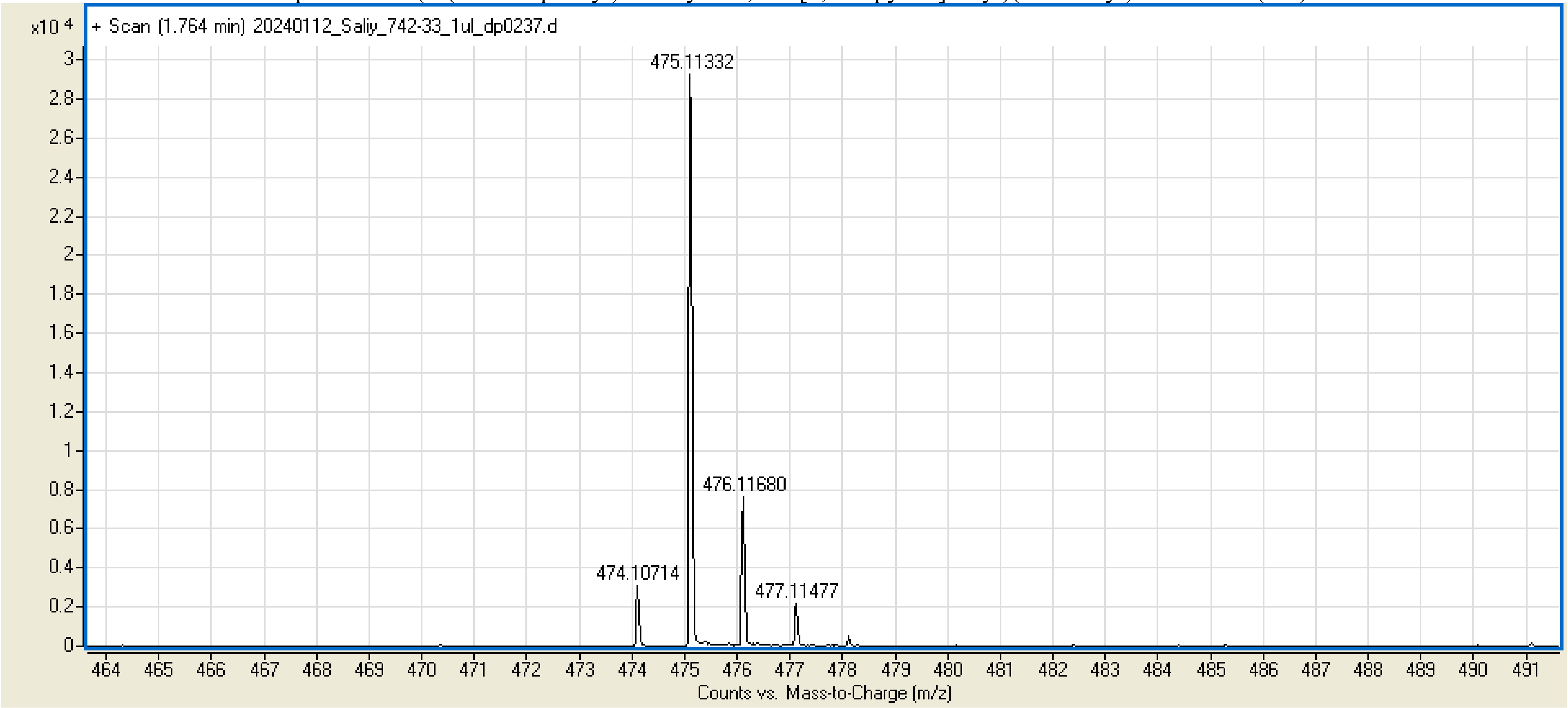

^{1}H NMR spectrum of (5-(4-chlorophenyl)-2'-tosyl-1*H*,1'*H*-[2,3'-bipyrrol]-4'-yl)(phenyl)methanone (**2n**) in $CDCl_3$.

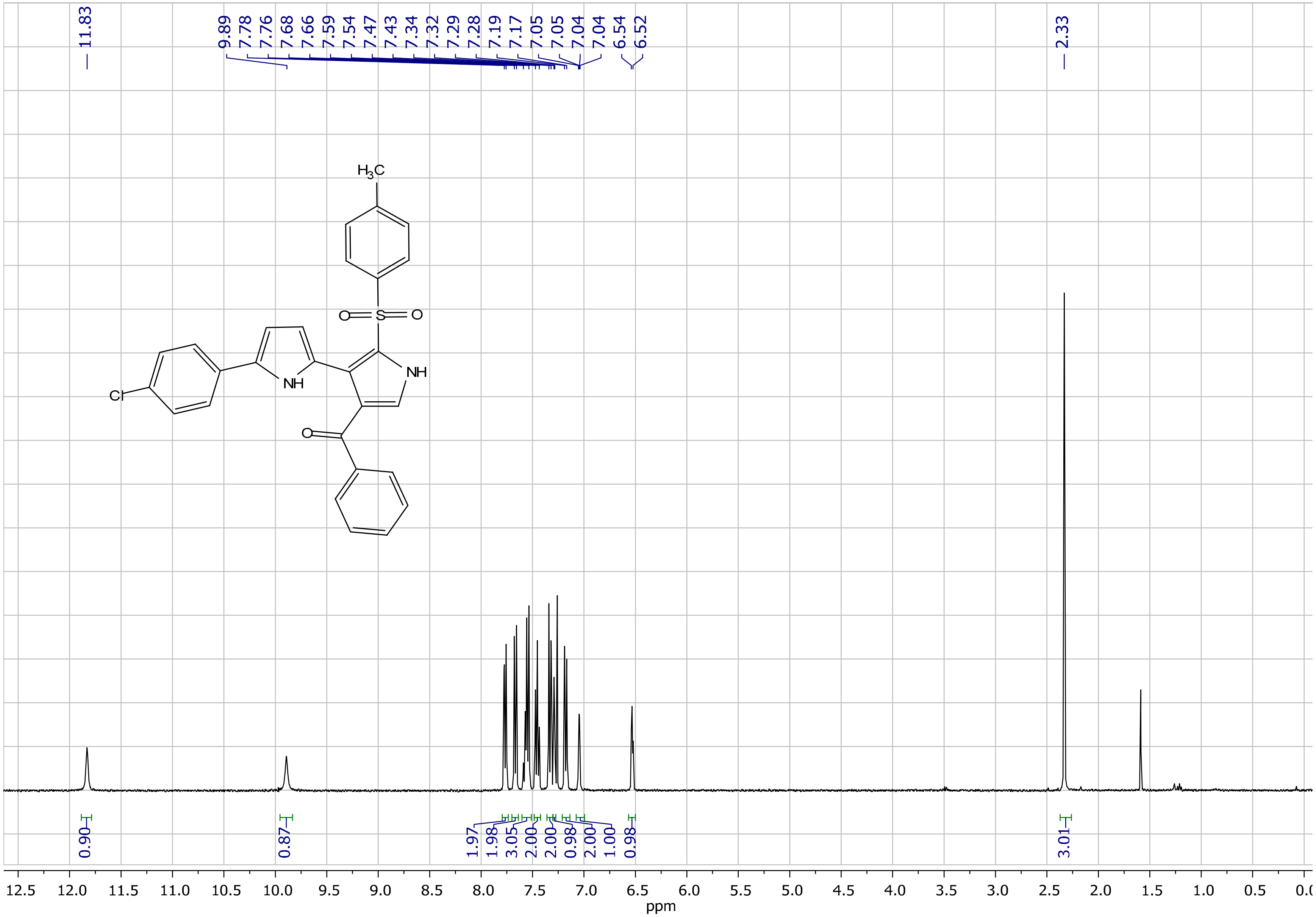

^{13}C NMR spectrum of (5-(4-chlorophenyl)-2'-tosyl-1*H*,1'*H*-[2,3'-bipyrrol]-4'-yl)(phenyl)methanone (**2n**) in $CDCl_3$.

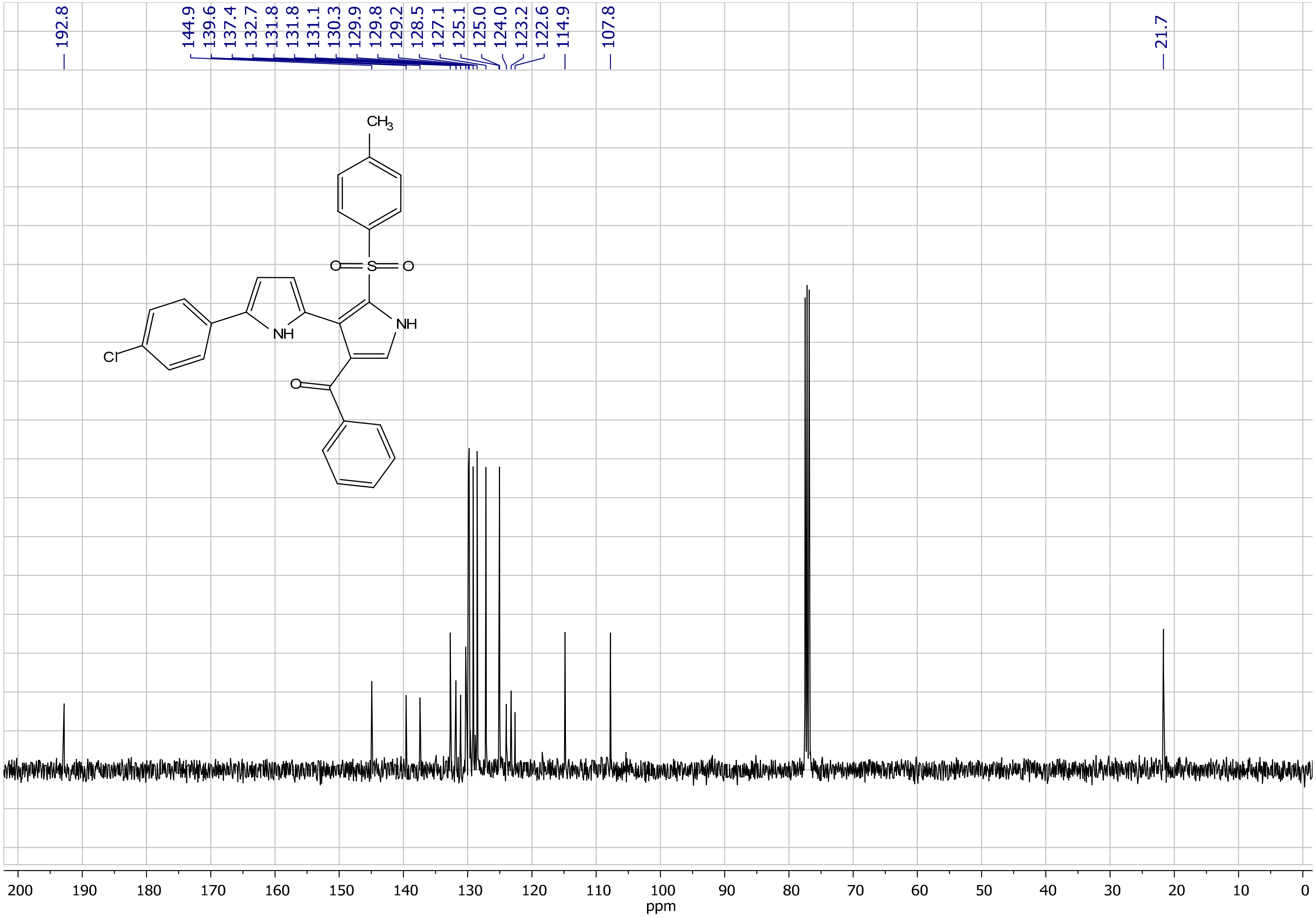

HRMS spectrum of (5-(4-chlorophenyl)-2'-tosyl-1*H*,1'*H*-[2,3'-bipyrrol]-4'-yl)(phenyl)methanone (**2n**)

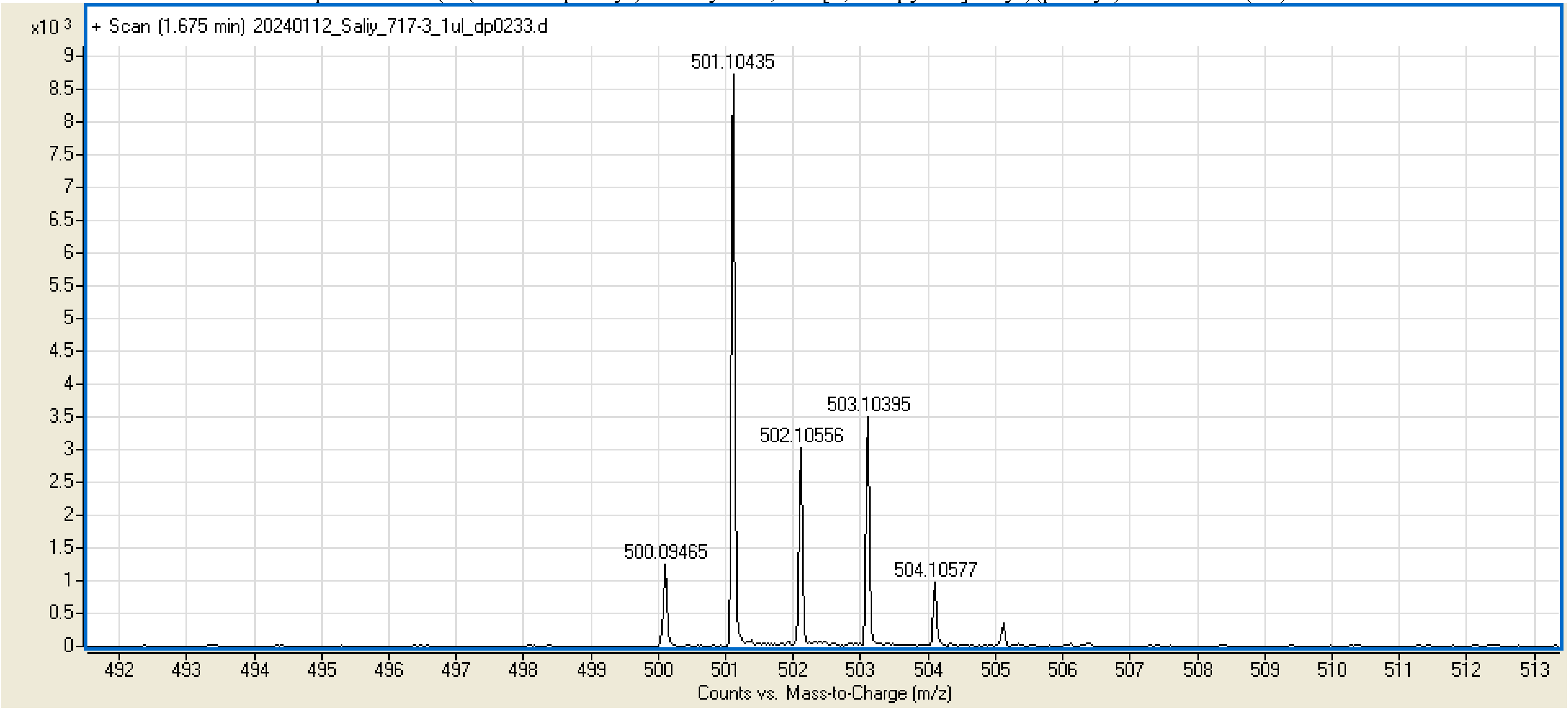

^{1}H NMR spectrum of (4,5-diphenyl-2'-tosyl-1*H*,1'*H*-[2,3'-bipyrrol]-4'-yl)(phenyl)methanone (**2o**) in $CDCl_3$.

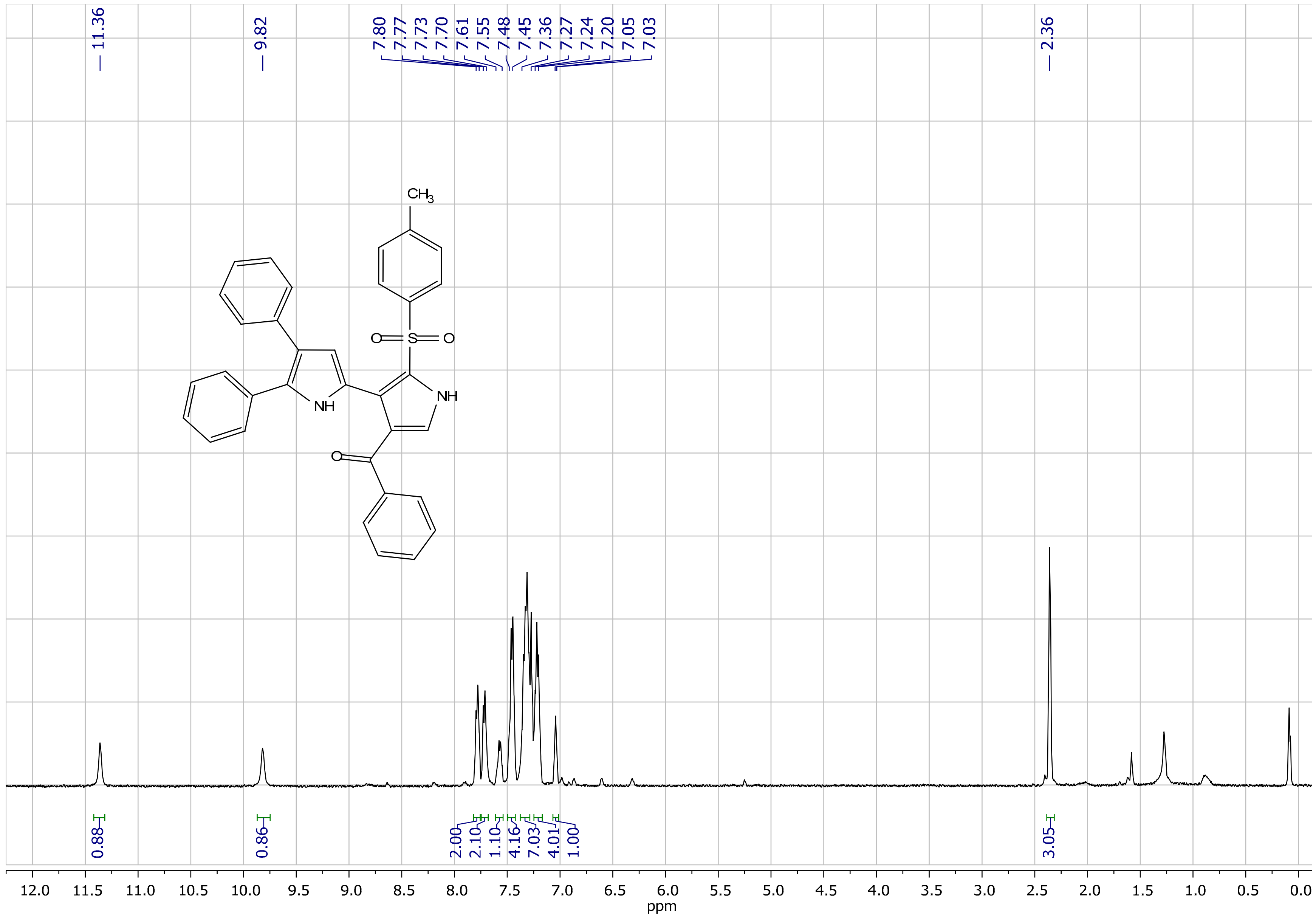

^{13}C NMR spectrum of (4,5-diphenyl-2'-tosyl-1*H*,1'*H*-[2,3'-bipyrrol]-4'-yl)(phenyl)methanone (**2o**) in $CDCl_3$.

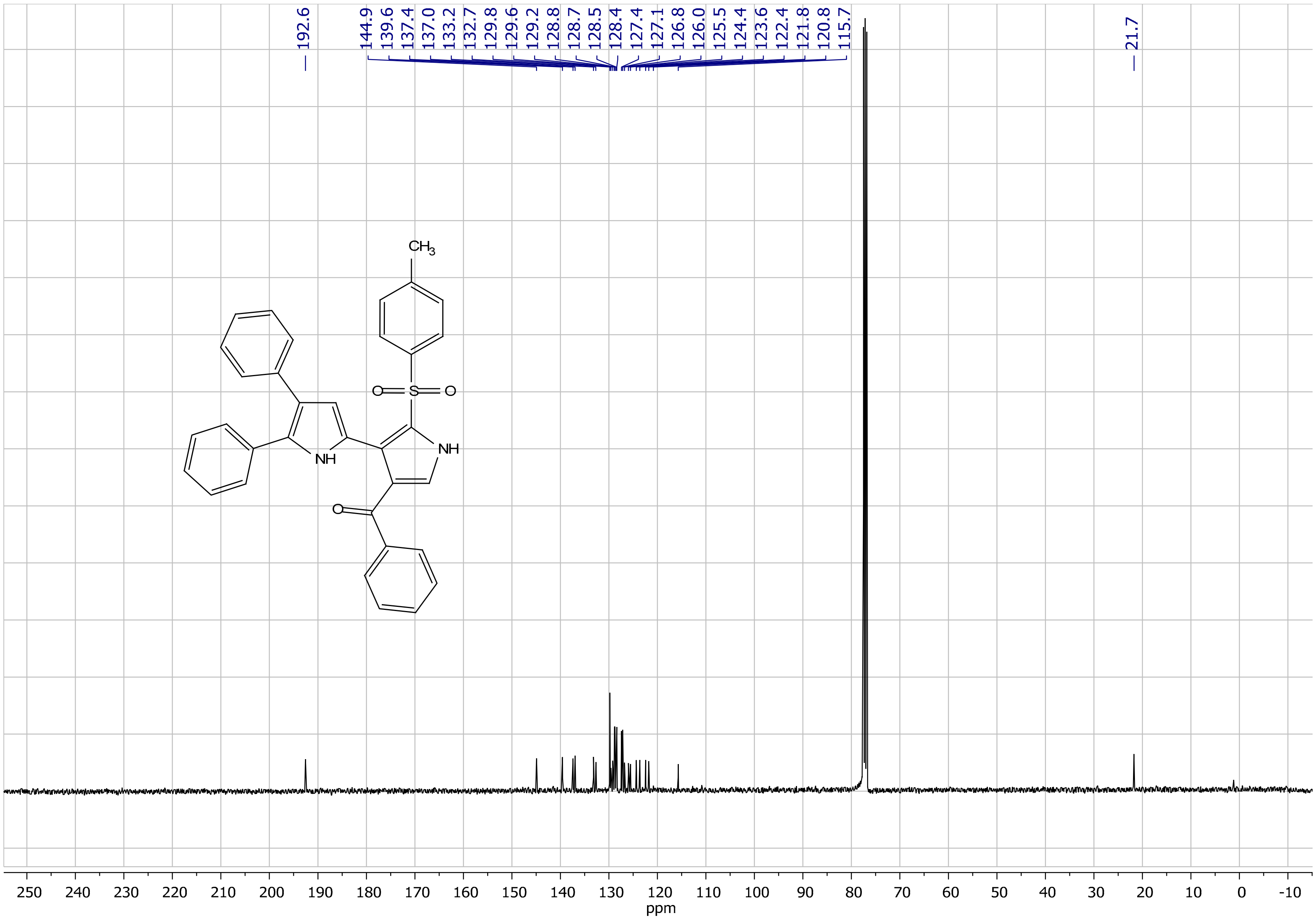

HRMS spectrum of (4,5-diphenyl-2'-tosyl-1*H*,1'*H*-[2,3'-bipyrrol]-4'-yl)(phenyl)methanone (**2o**)

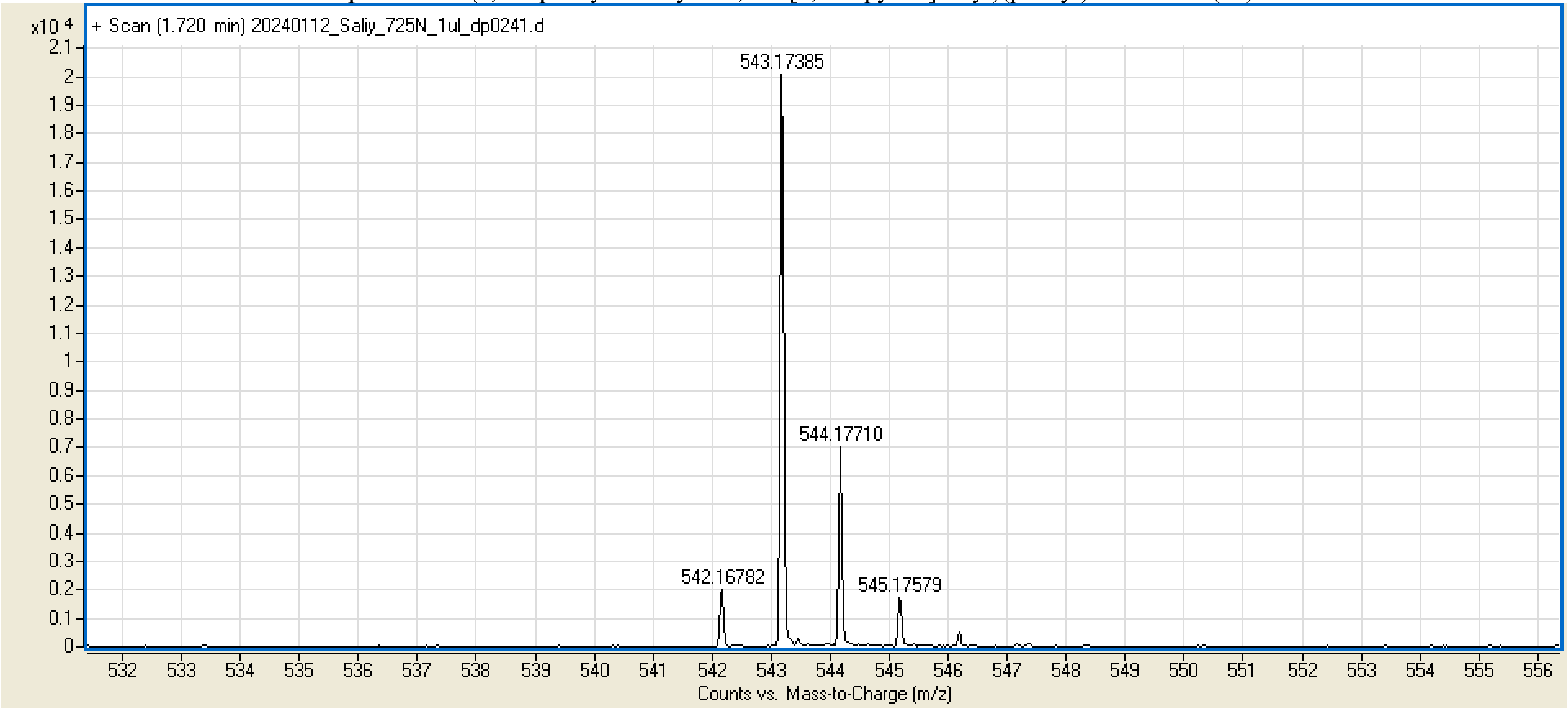

^{1}H NMR spectrum of (*E*)-1-phenyl-2-(1*H*-pyrrolo[1,2-c]imidazol-1-ylidene)-3-tosylpropan-1-one (**3a**) in $CDCl_3$.

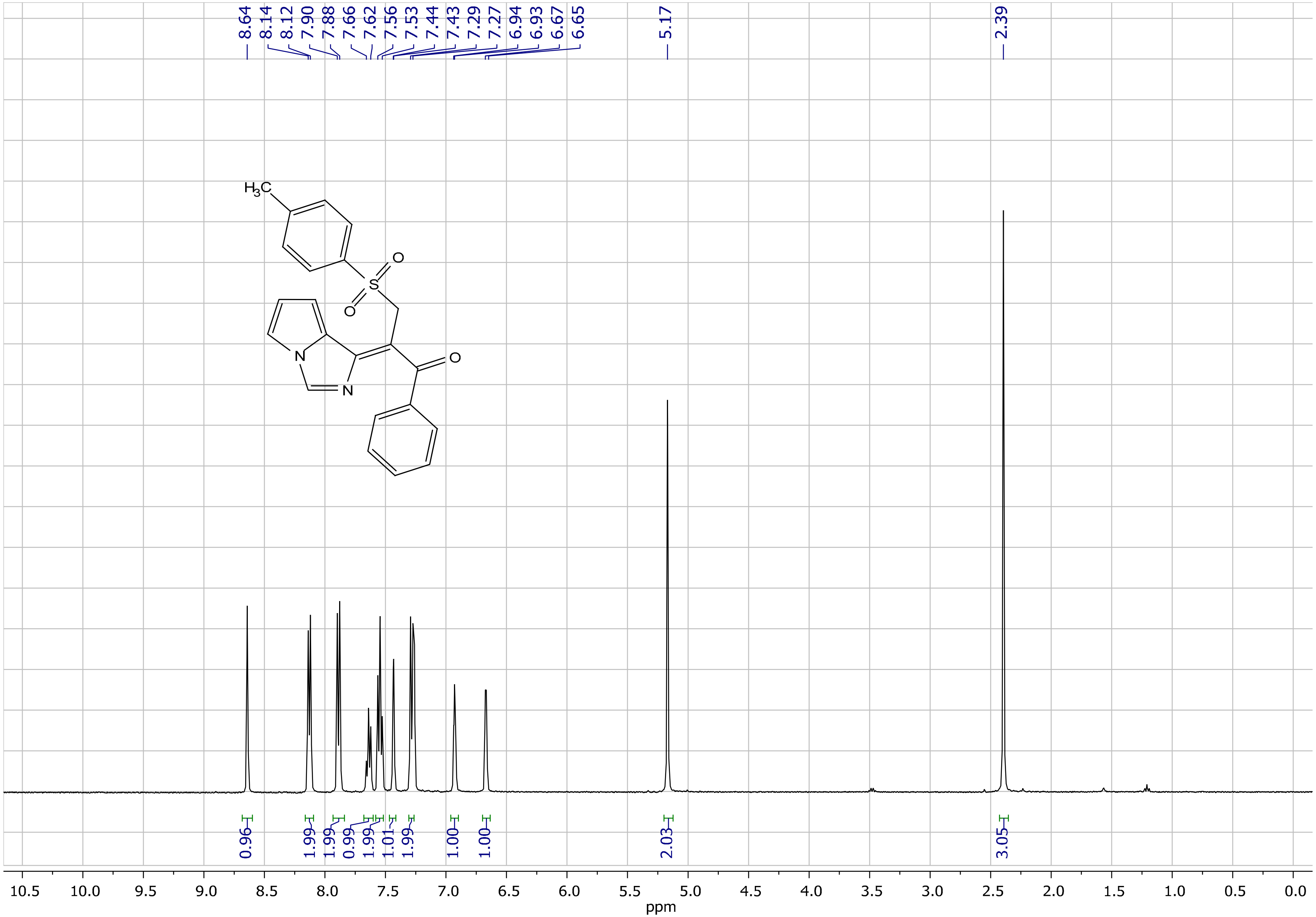

^{13}C NMR spectrum of (*E*)-1-phenyl-2-(1*H*-pyrrolo[1,2-c]imidazol-1-ylidene)-3-tosylpropan-1-one (**3a**) in $CDCl_3$.

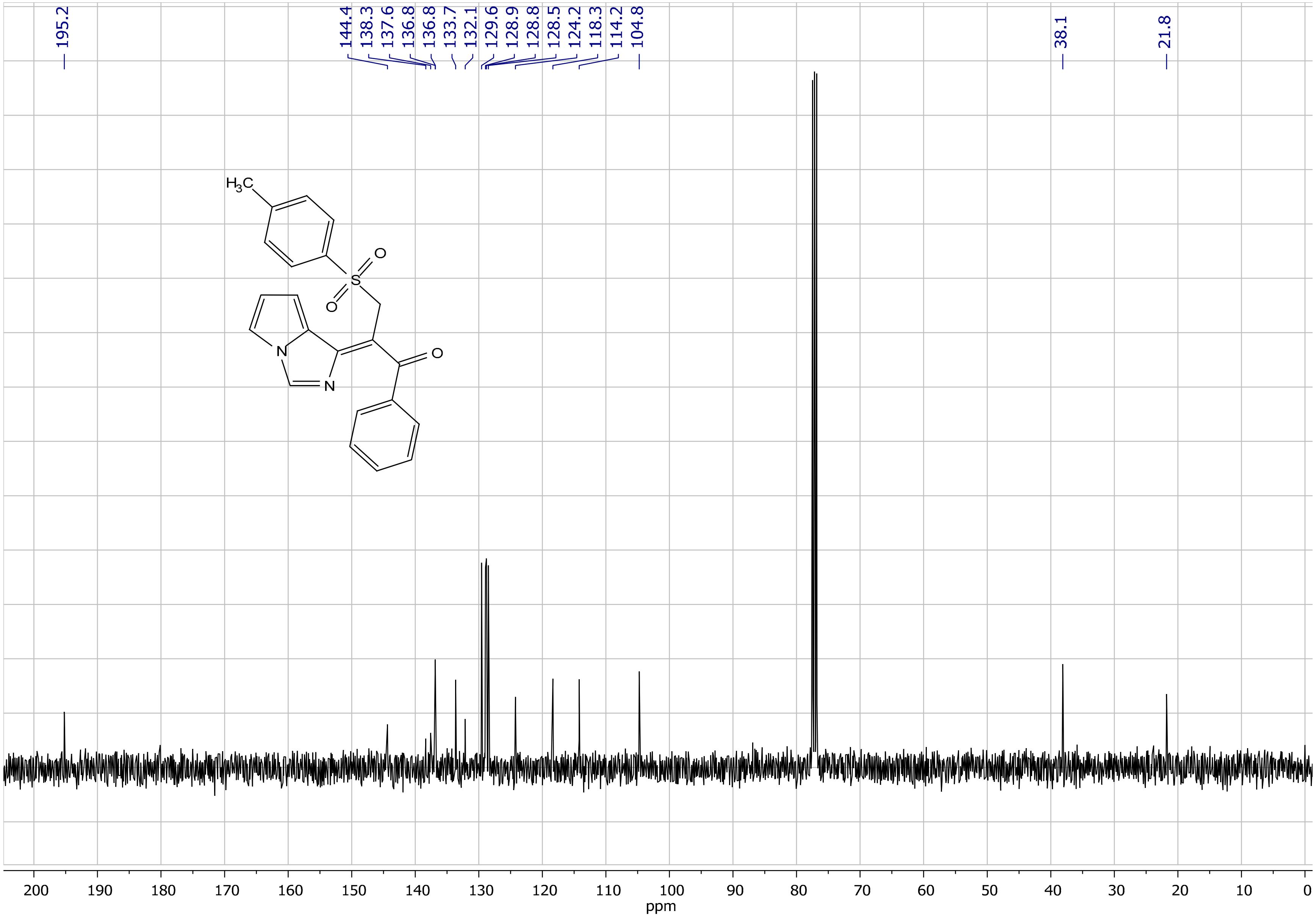

HRMS spectrum of (*E*)-1-phenyl-2-(1*H*-pyrrolo[1,2-c]imidazol-1-ylidene)-3-tosylpropan-1-one (**3a**)

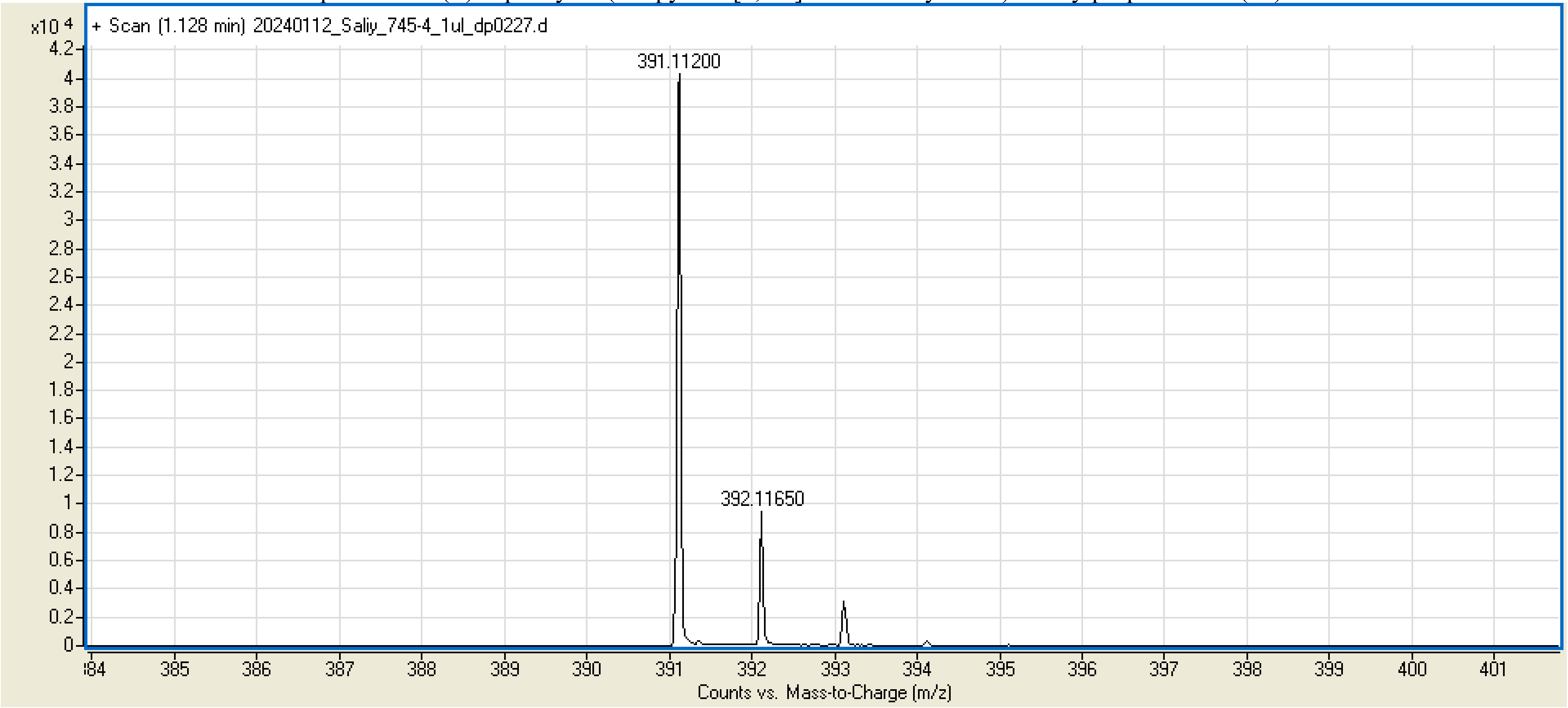

^{1}H NMR spectrum of (*E*)-1-(furan-2-yl)-2-(1*H*-pyrrolo[1,2-c]imidazol-1-ylidene)-3-tosylpropan-1-one (**3b**) in $CDCl_3$.

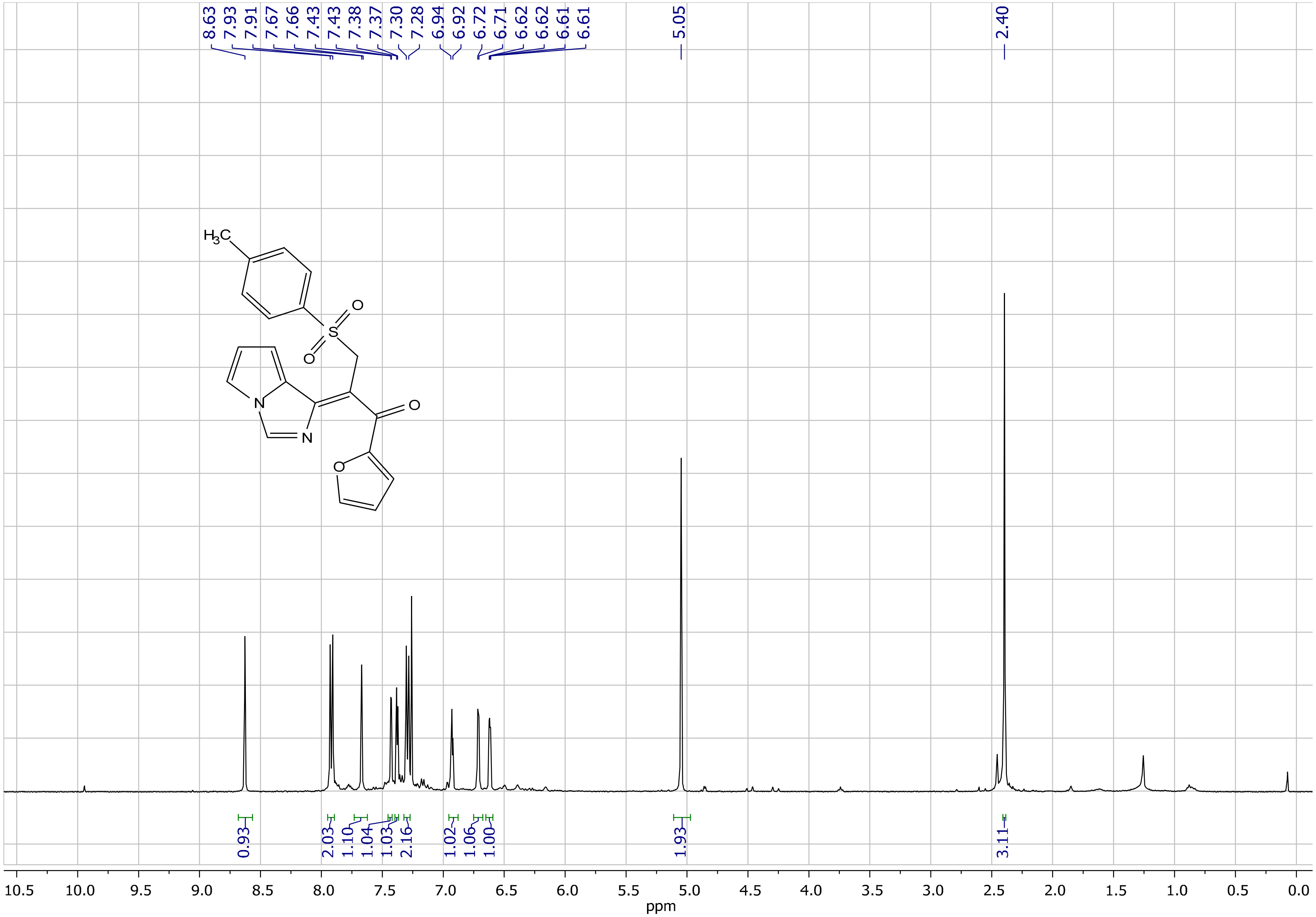

^{13}C NMR spectrum of (*E*)-1-(furan-2-yl)-2-(1*H*-pyrrolo[1,2-c]imidazol-1-ylidene)-3-tosylpropan-1-one (**3b**) in $CDCl_3$.

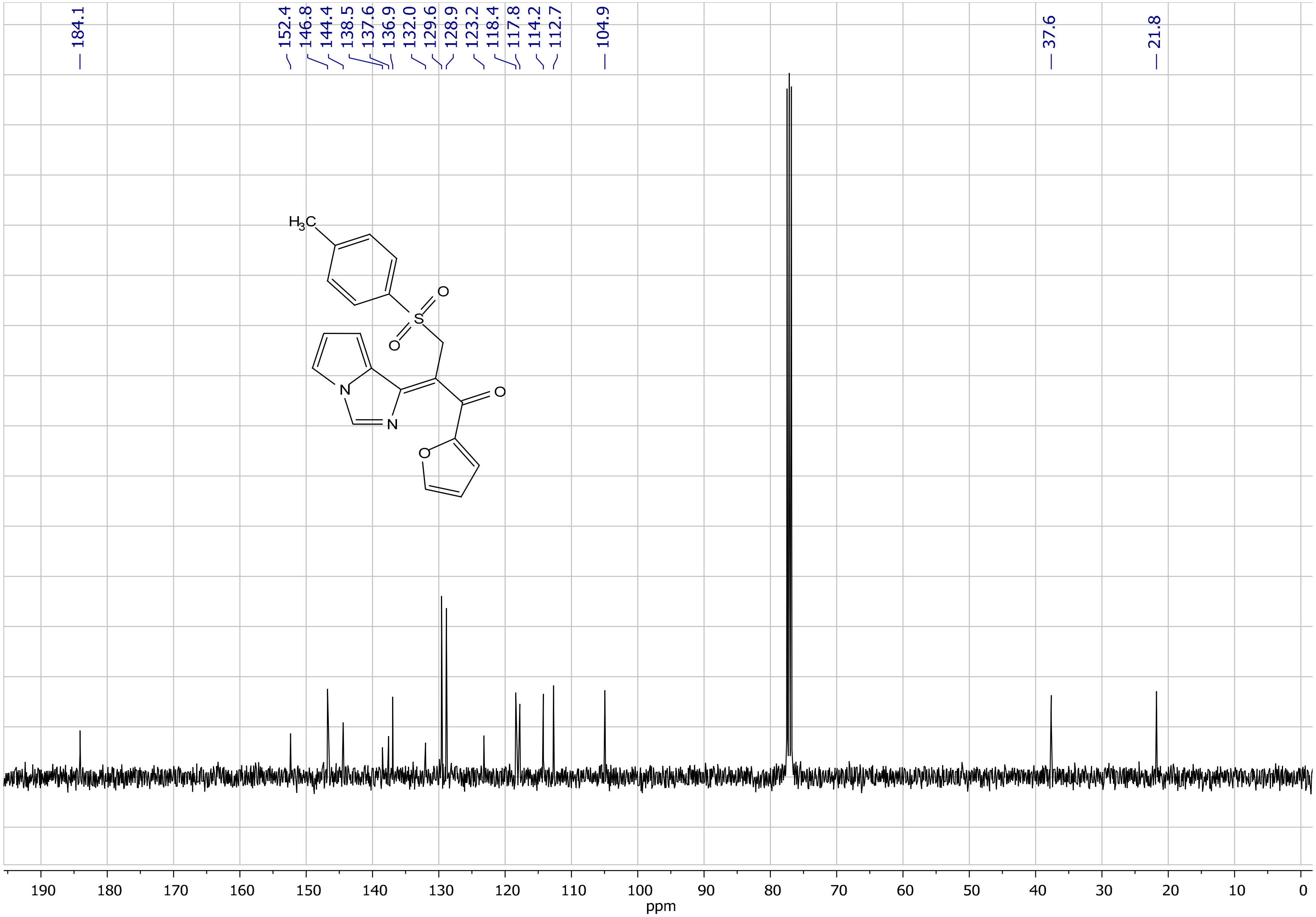

HRMS spectrum of (*E*)-1-(furan-2-yl)-2-(1*H*-pyrrolo[1,2-c]imidazol-1-ylidene)-3-tosylpropan-1-one (**3b**)

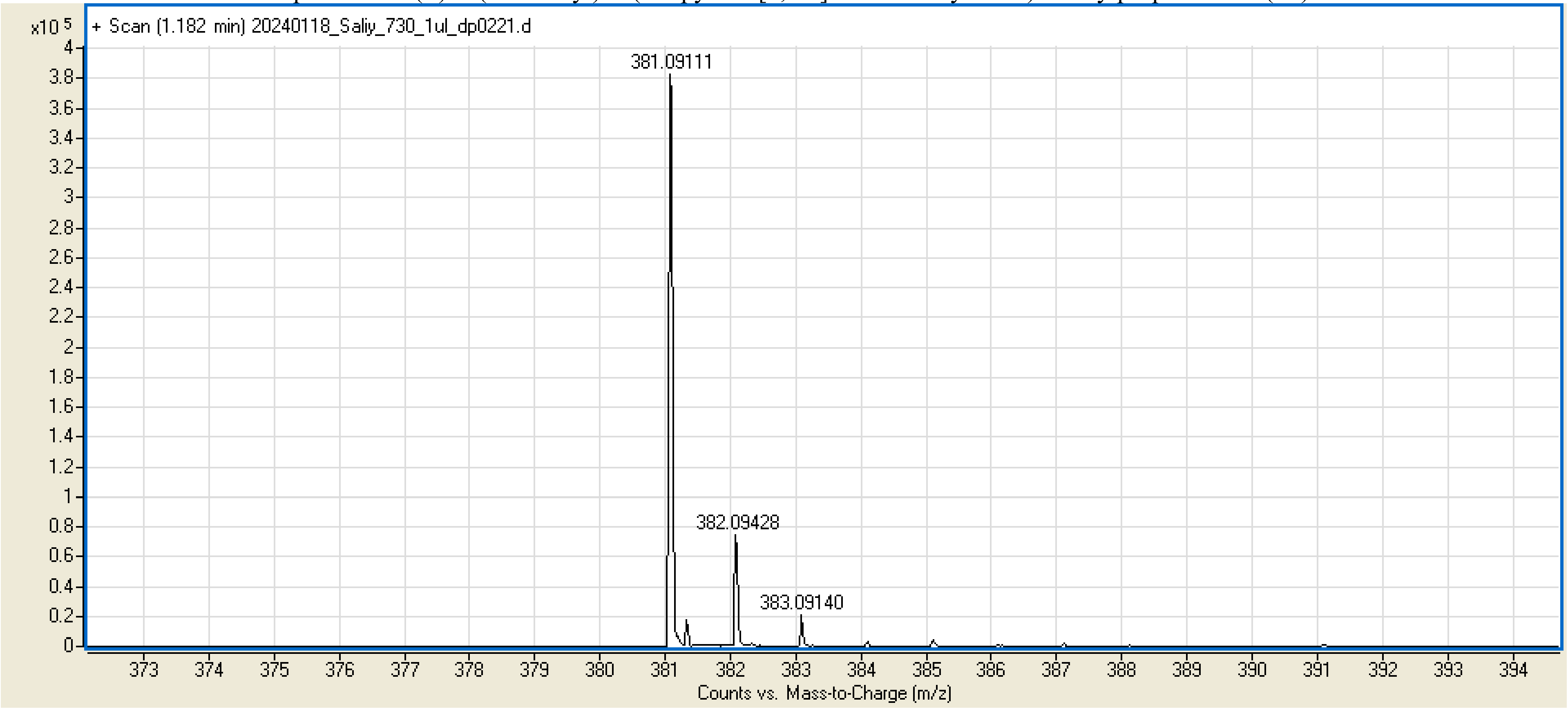

^{1}H NMR spectrum of (*E*)-2-(6-ethyl-5-propyl-1*H*-pyrrolo[1,2-c]imidazol-1-ylidene)-1-phenyl-3-tosylpropan-1-one (**3c**) in $CDCl_3$.

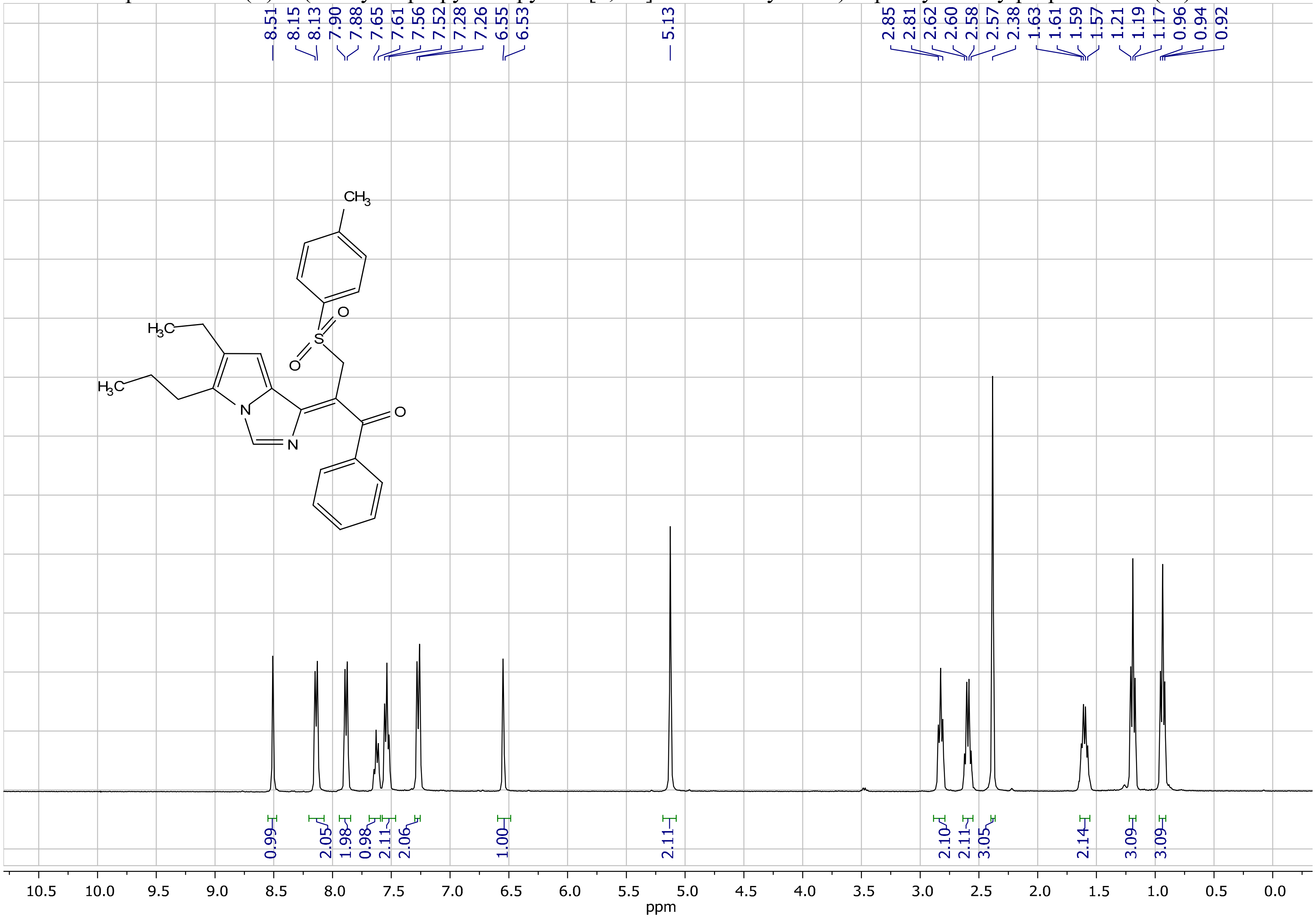

^{13}C NMR spectrum of (*E*)-2-(6-ethyl-5-propyl-1*H*-pyrrolo[1,2-c]imidazol-1-ylidene)-1-phenyl-3-tosylpropan-1-one (**3c**) in $CDCl_3$.

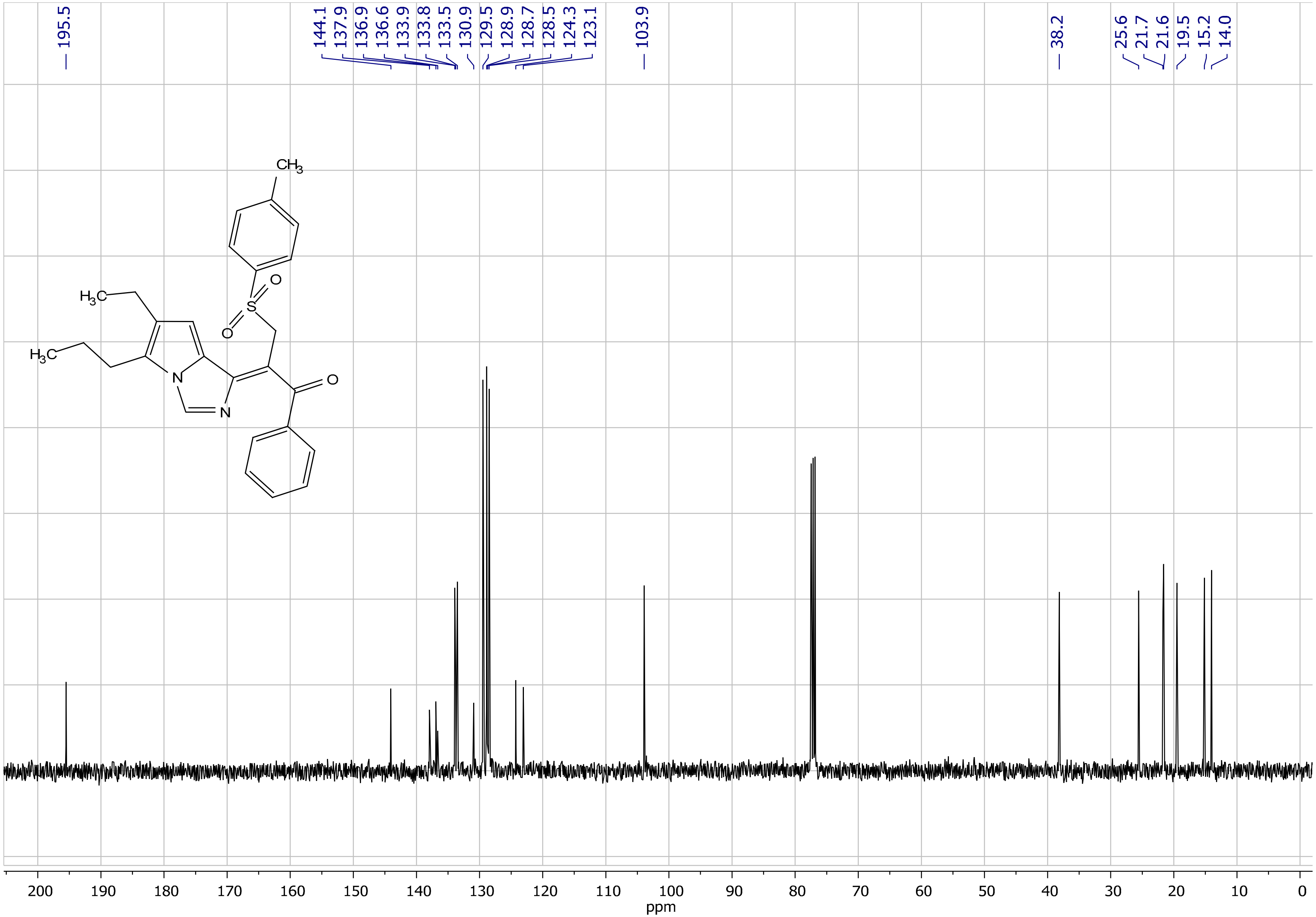

HRMS spectrum of (*E*)-2-(6-ethyl-5-propyl-1*H*-pyrrolo[1,2-c]imidazol-1-ylidene)-1-phenyl-3-tosylpropan-1-one (**3c**)

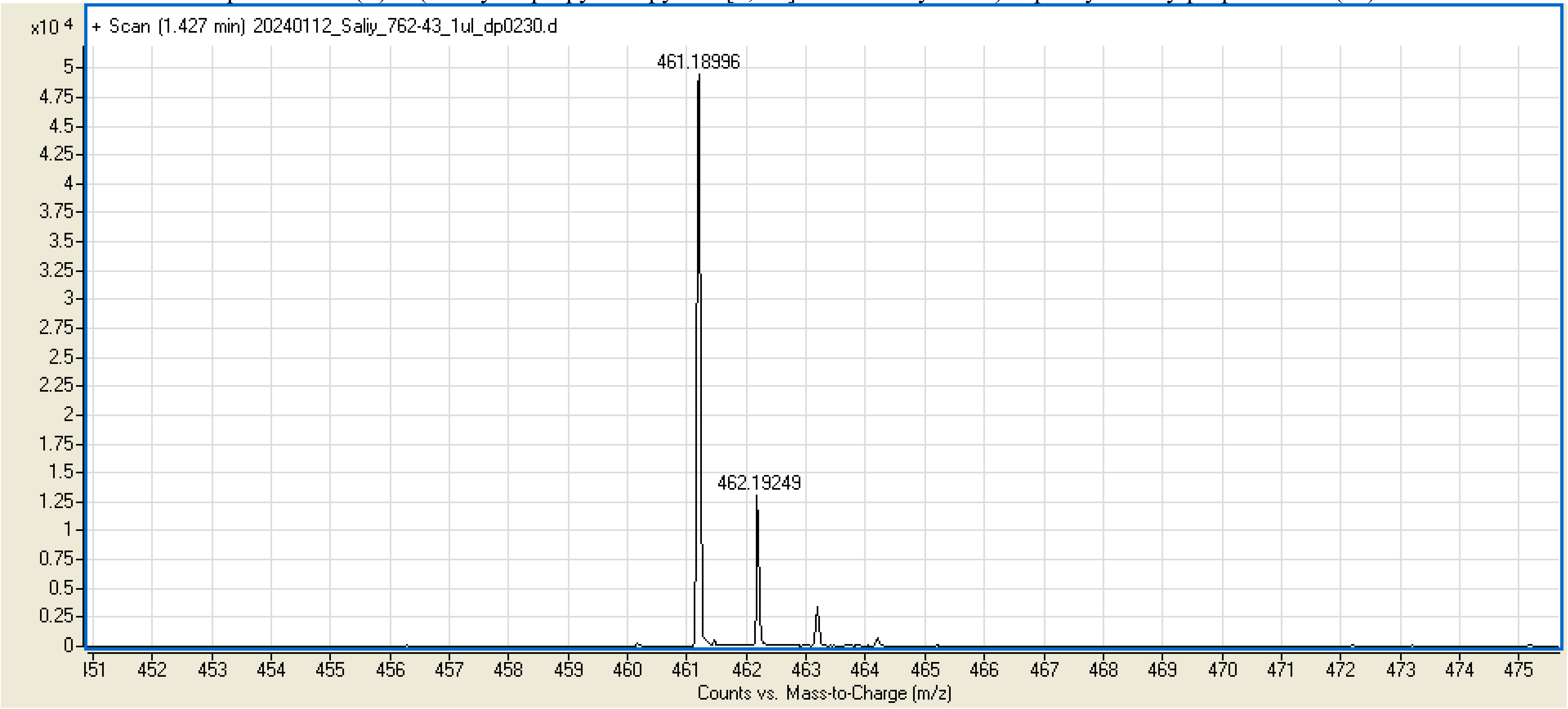

^{1}H NMR spectrum of (*E*)-1-phenyl-2-(5-phenyl-1*H*-pyrrolo[1,2-c]imidazol-1-ylidene)-3-tosylpropan-1-one (**3d**) in $CDCl_3$.

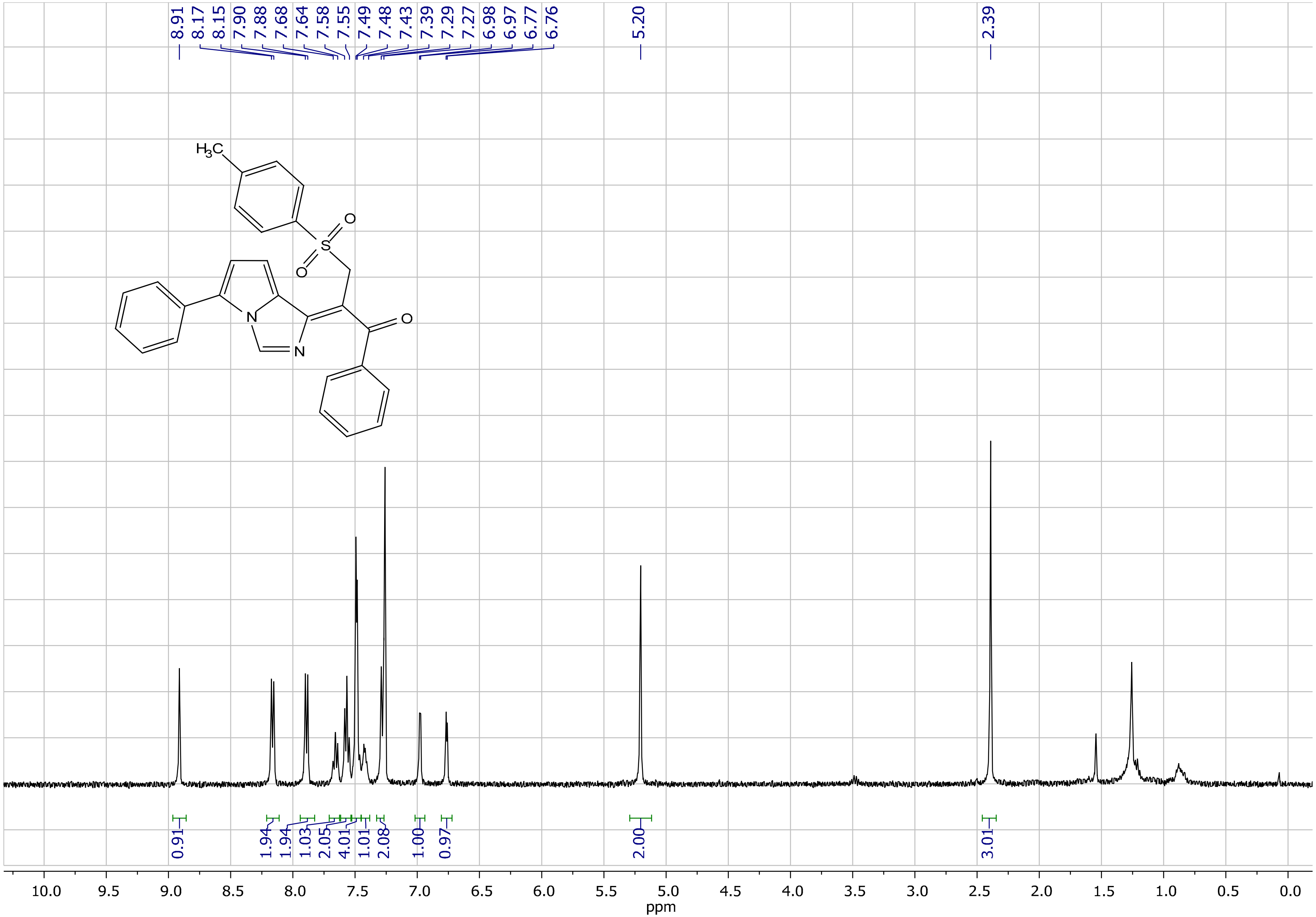

^{13}C NMR spectrum of (*E*)-1-phenyl-2-(5-phenyl-1*H*-pyrrolo[1,2-c]imidazol-1-ylidene)-3-tosylpropan-1-one (**3d**) in $CDCl_3$.

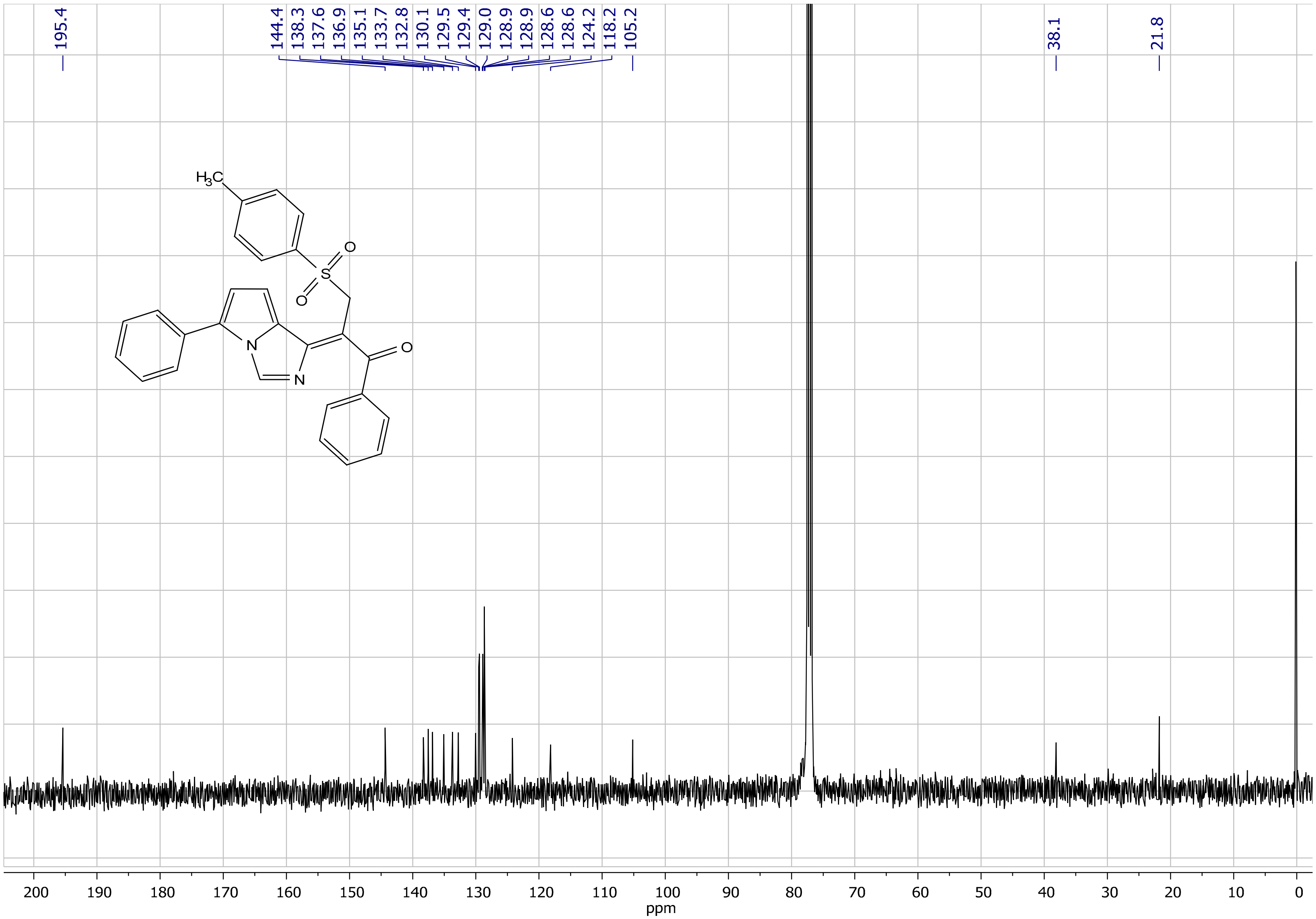

HRMS spectrum of (*E*)-1-phenyl-2-(5-phenyl-1*H*-pyrrolo[1,2-c]imidazol-1-ylidene)-3-tosylpropan-1-one (**3d**)

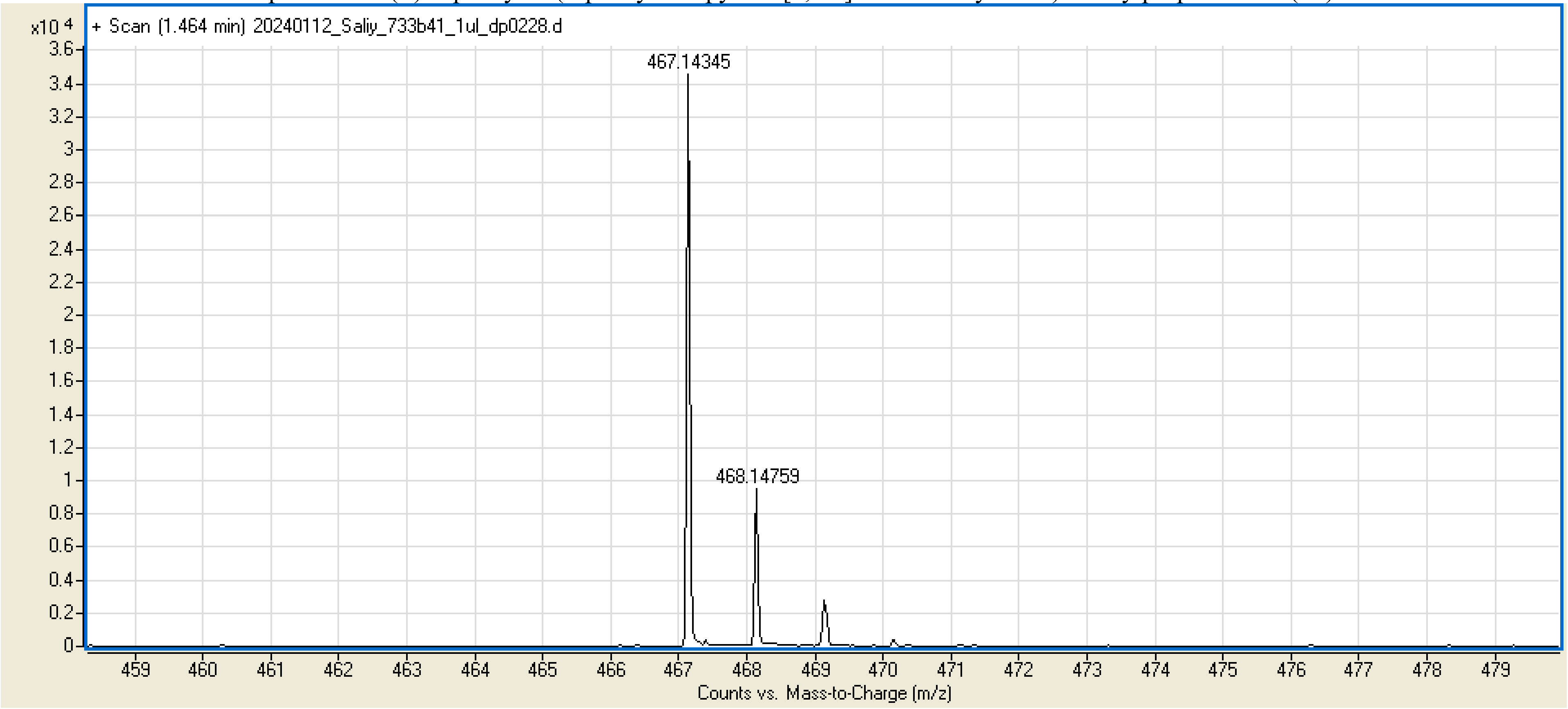

^{1}H NMR spectrum of (*E*)-1-(furan-2-yl)-2-(5-phenyl-1*H*-pyrrolo[1,2-c]imidazol-1-ylidene)-3-tosylpropan-1-one (**3e**) in $CDCl_3$.

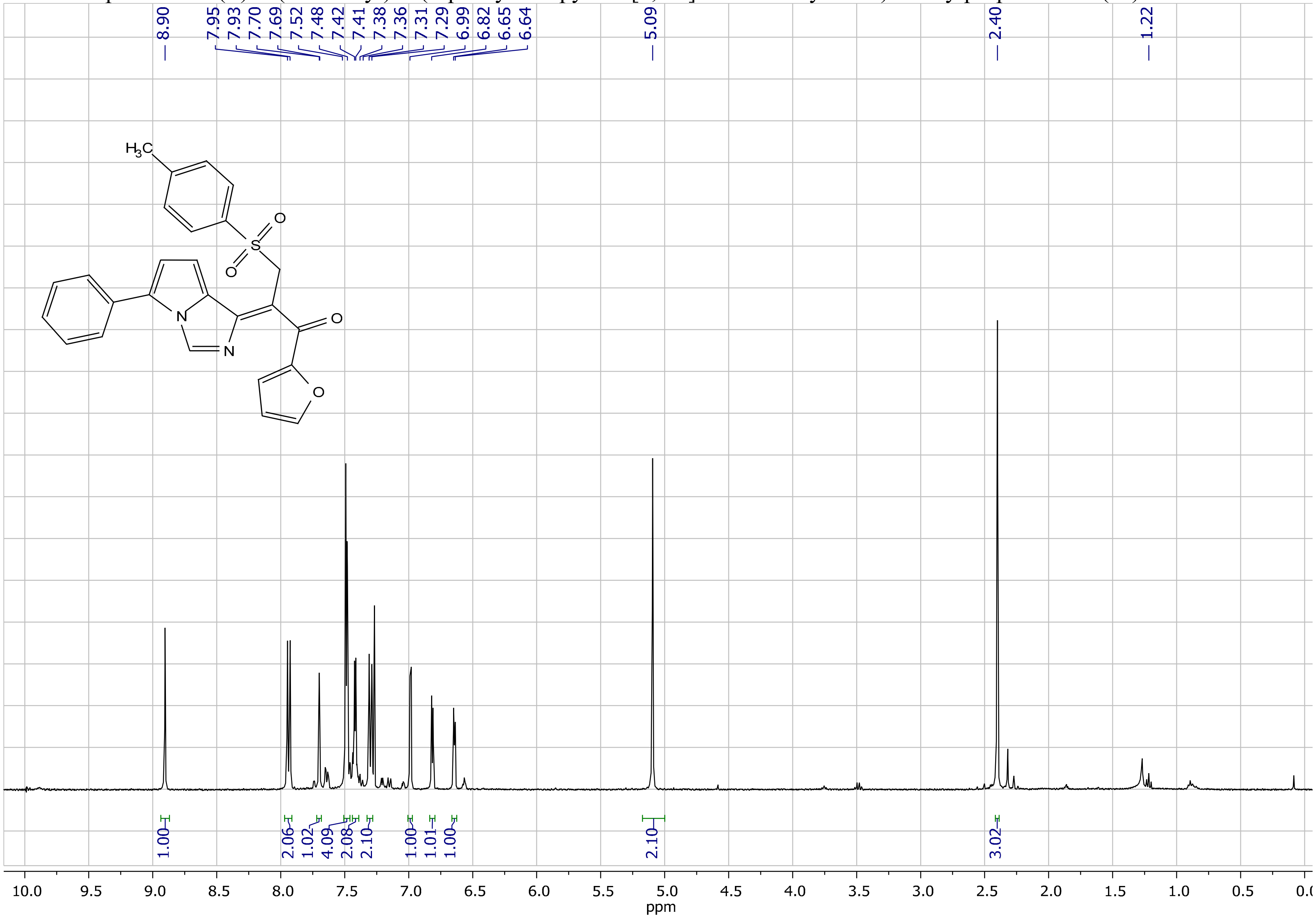

^{13}C NMR spectrum of (*E*)-1-(furan-2-yl)-2-(5-phenyl-1*H*-pyrrolo[1,2-c]imidazol-1-ylidene)-3-tosylpropan-1-one (**3e**) in $CDCl_3$.

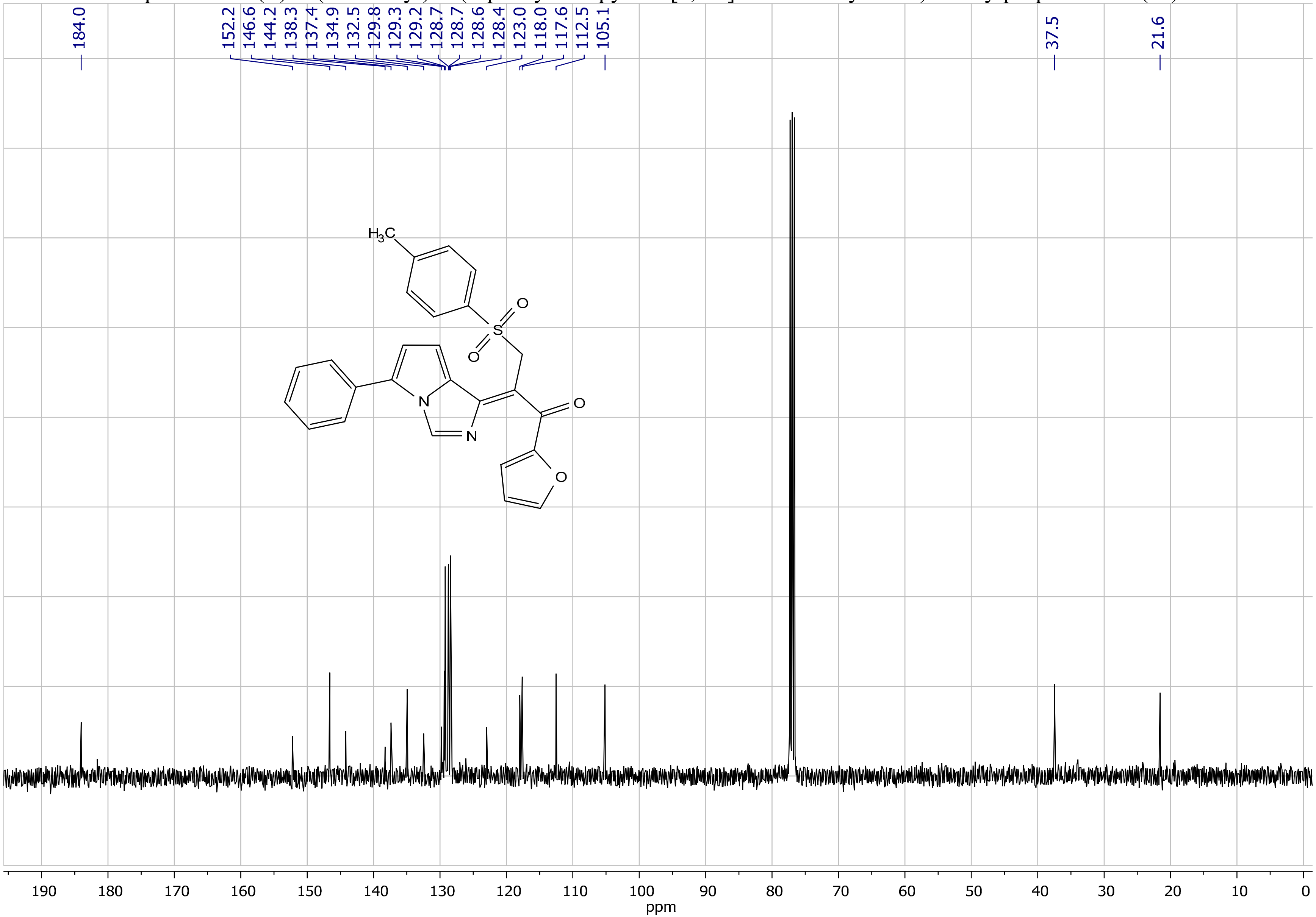

HRMS spectrum of (*E*)-1-(furan-2-yl)-2-(5-phenyl-1*H*-pyrrolo[1,2-c]imidazol-1-ylidene)-3-tosylpropan-1-one (**3e**)

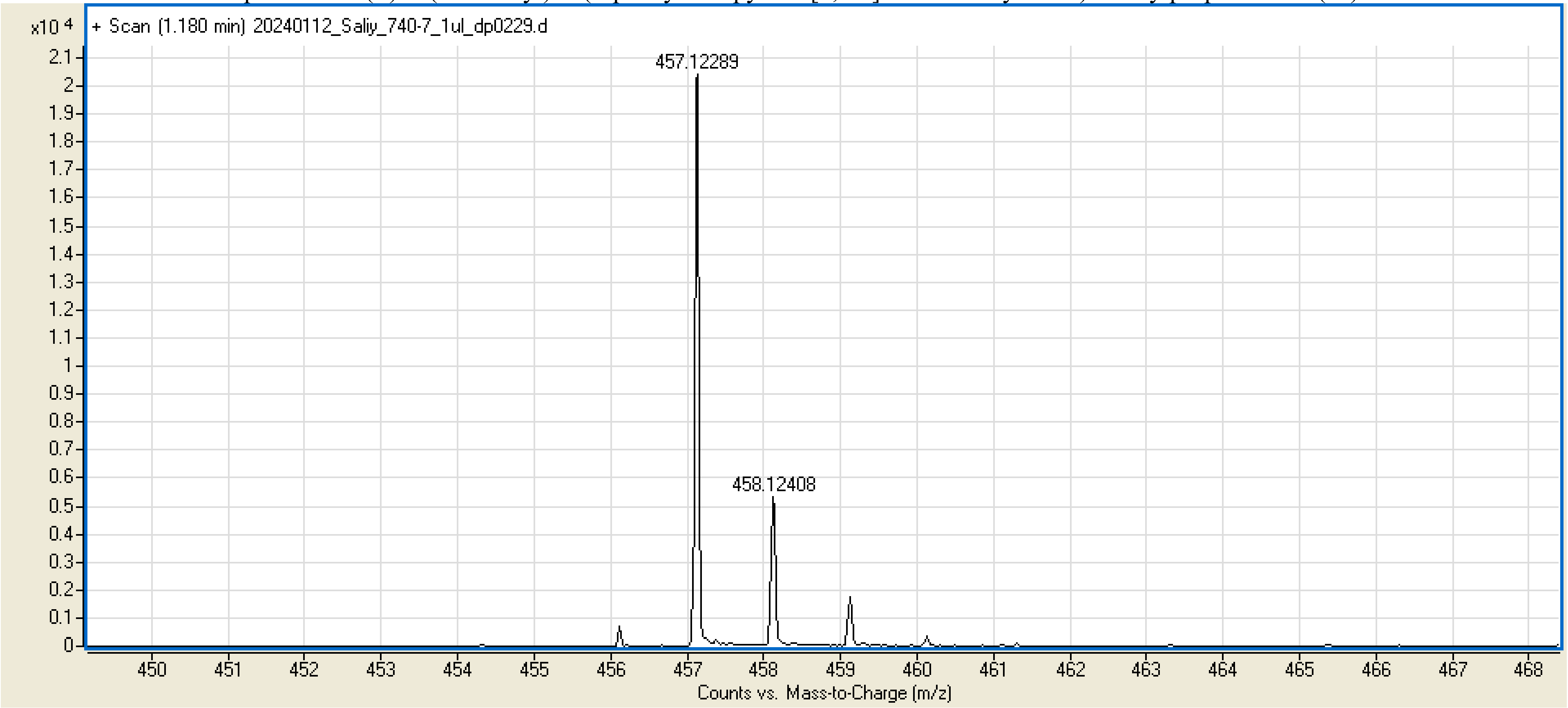

^{1}H NMR spectrum of (*E*)-2-(5-phenyl-1*H*-pyrrolo[1,2-c]imidazol-1-ylidene)-1-(thiophen-2-yl)-3-tosylpropan-1-one (**3f**) in $CDCl_3$.

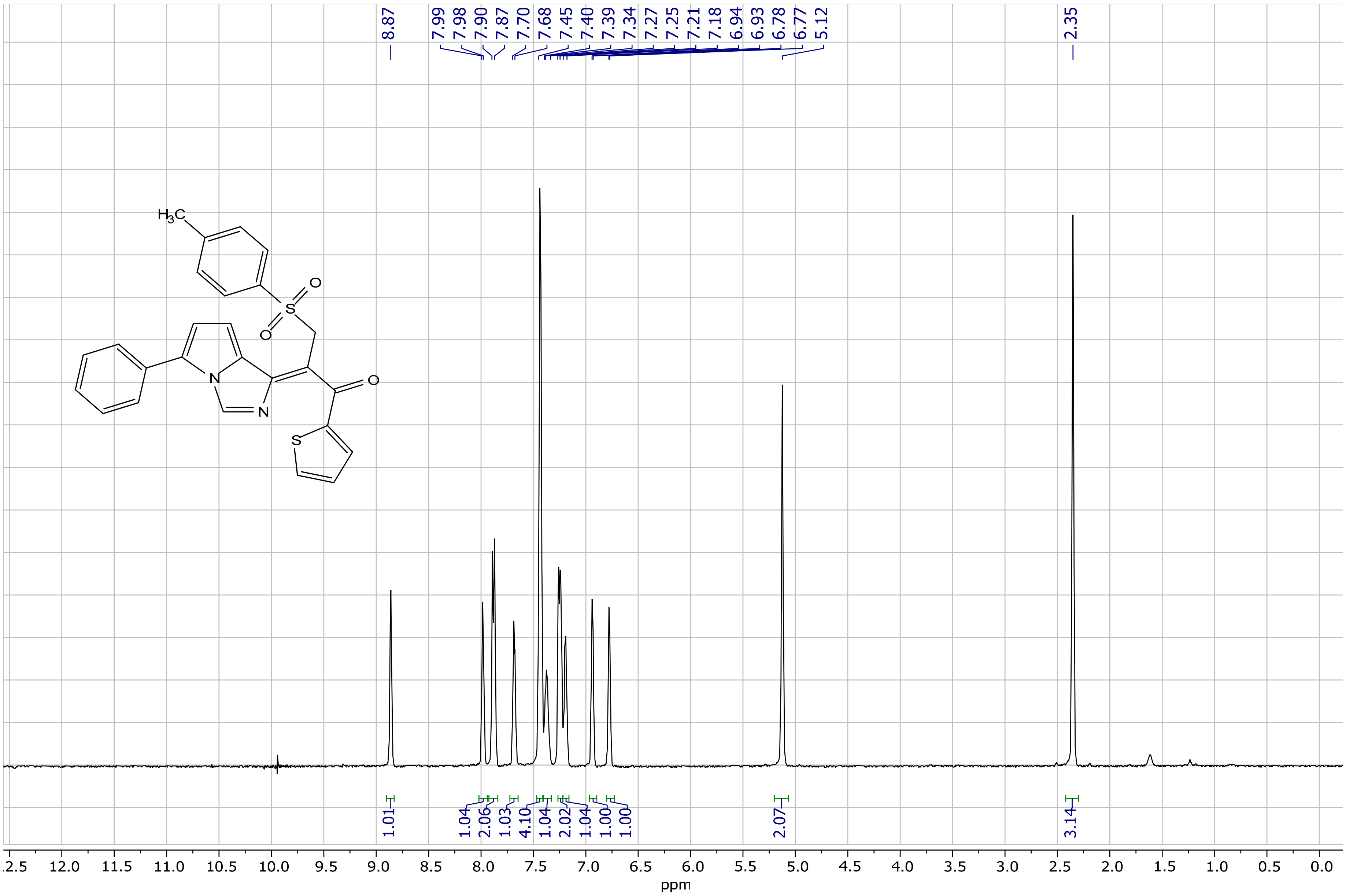

^{13}C NMR spectrum of (*E*)-2-(5-phenyl-1*H*-pyrrolo[1,2-c]imidazol-1-ylidene)-1-(thiophen-2-yl)-3-tosylpropan-1-one (**3f**) in $CDCl_3$.

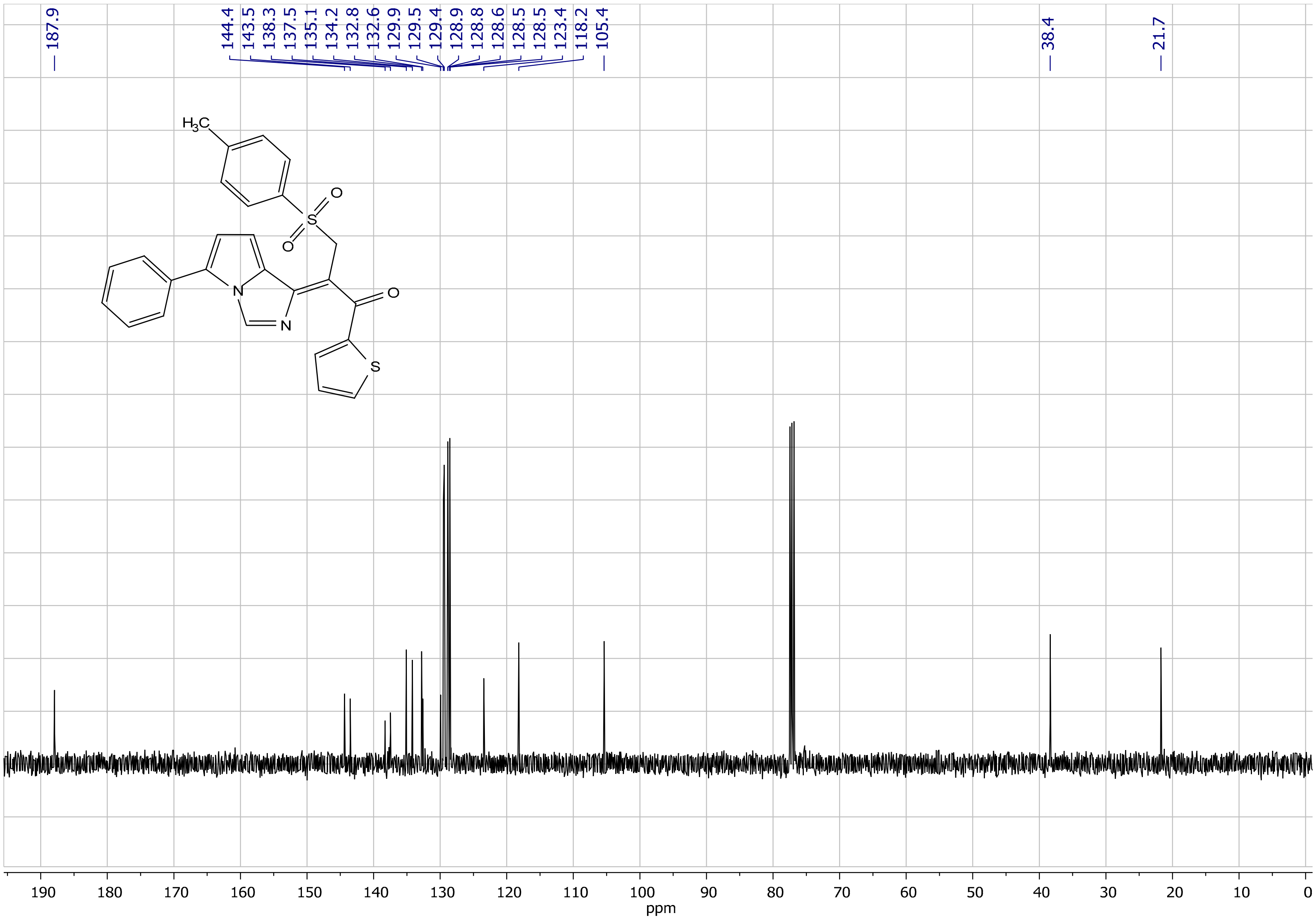

HRMS spectrum of (*E*)-2-(5-phenyl-1*H*-pyrrolo[1,2-c]imidazol-1-ylidene)-1-(thiophen-2-yl)-3-tosylpropan-1-one (**3f**)

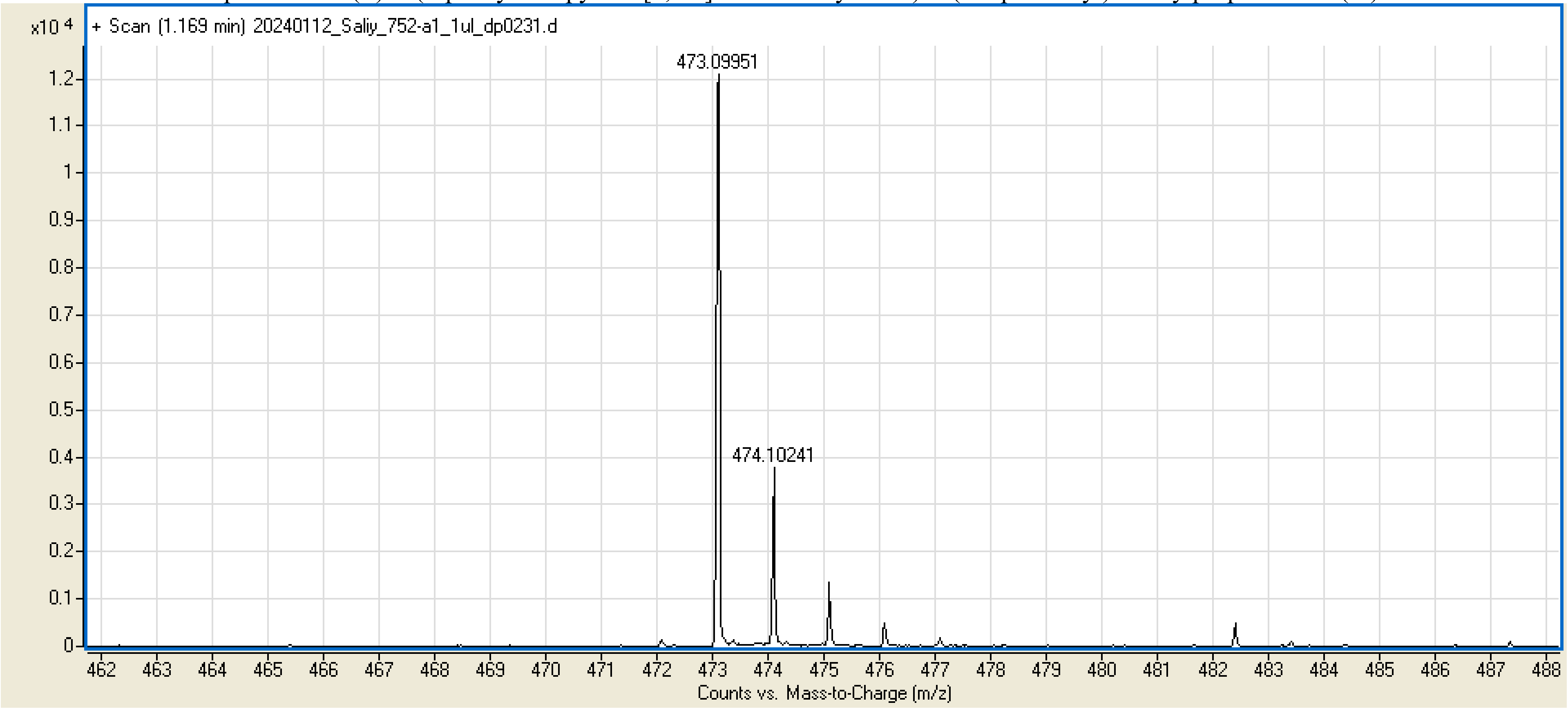

^{1}H NMR spectrum of (*E*)-2-(5-(4-(dimethylamino)phenyl)-1*H*-pyrrolo[1,2-c]imidazol-1-ylidene)-1-phenyl-3-tosylpropan-1-one (**3g**) in $CDCl_3$.

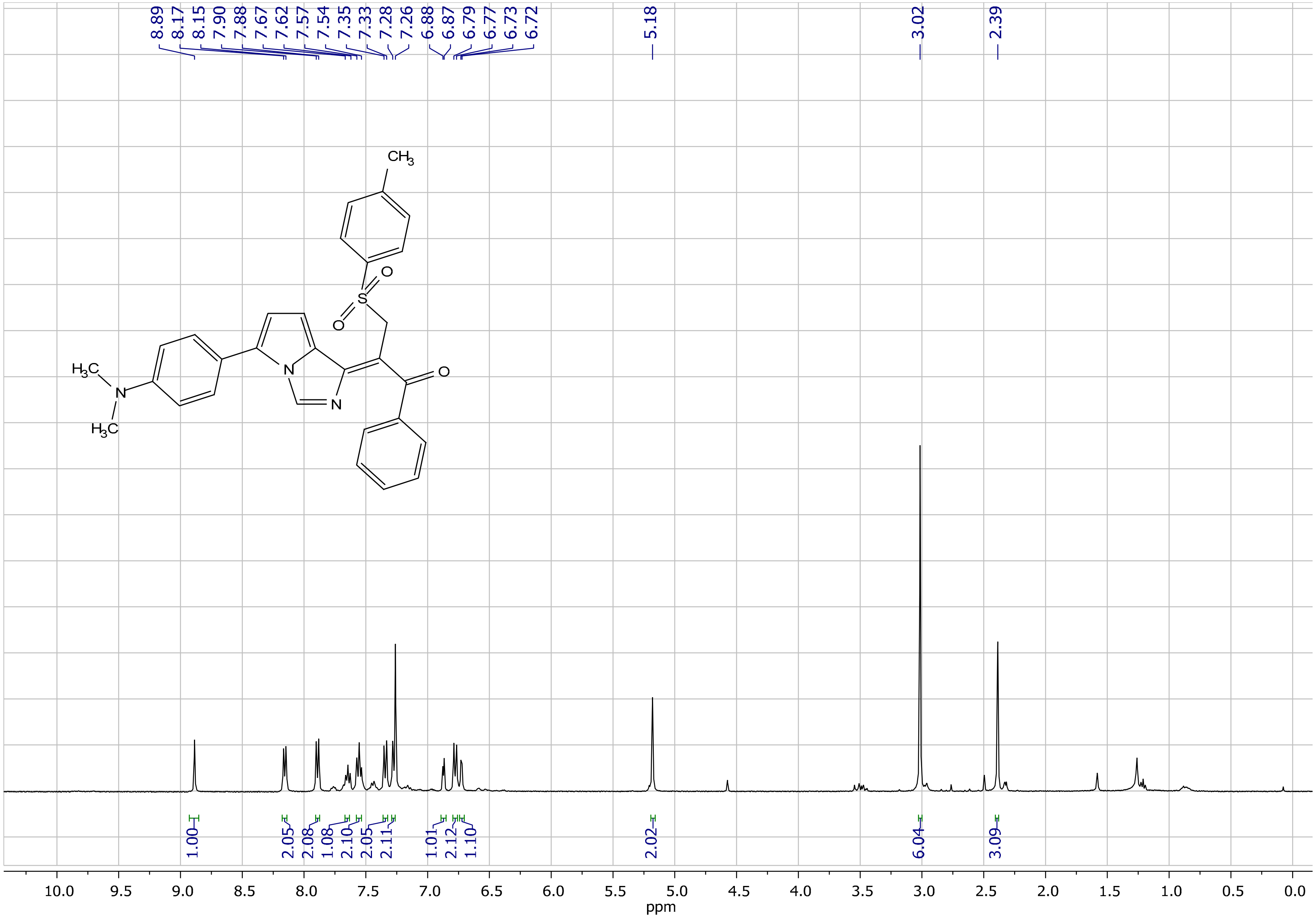

^{13}C NMR spectrum of (*E*)-2-(5-(4-(dimethylamino)phenyl)-1*H*-pyrrolo[1,2-c]imidazol-1-ylidene)-1-phenyl-3-tosylpropan-1-one (**3g**) in $CDCl_3$.

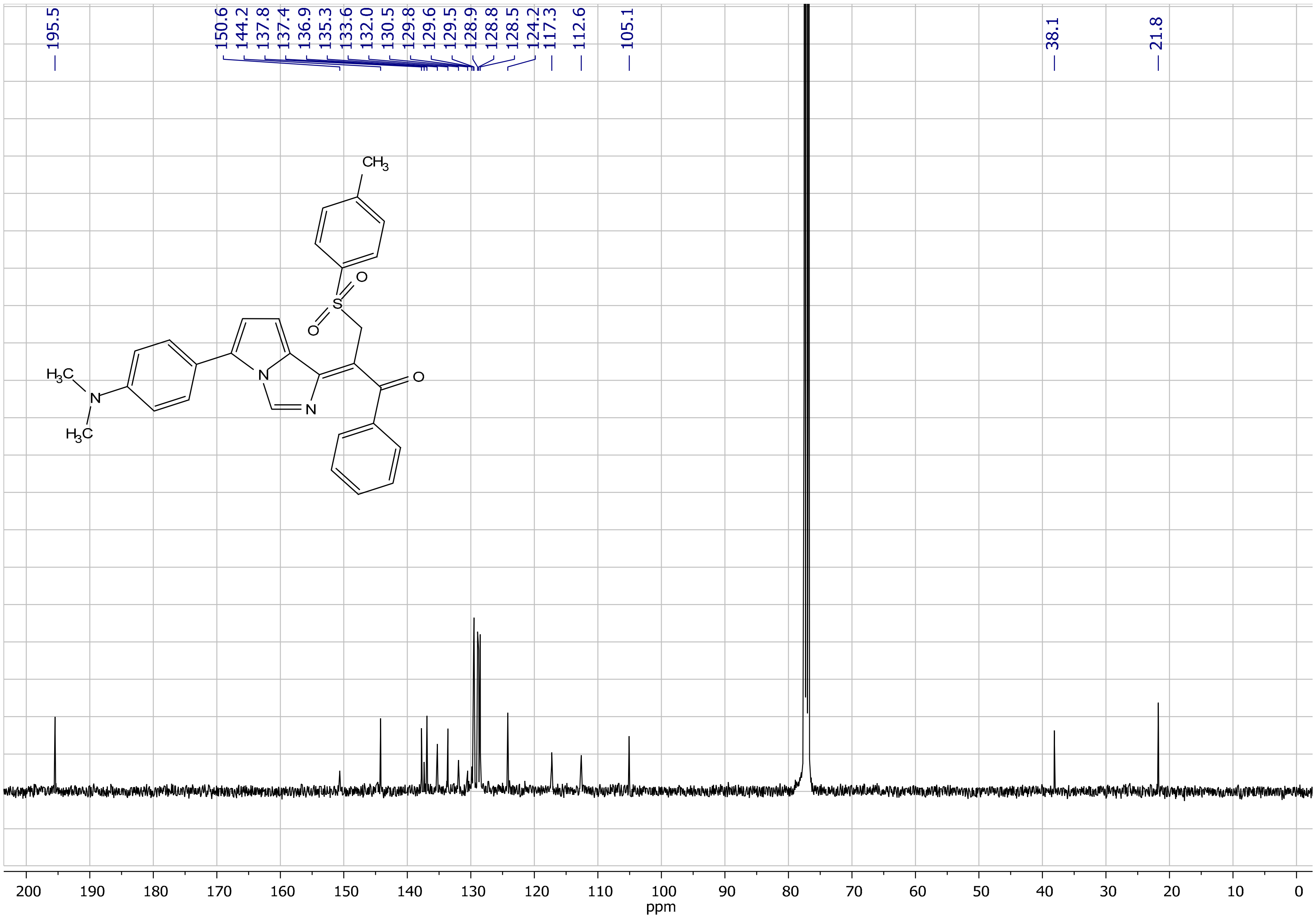

HRMS spectrum of (*E*)-2-(5-(4-(dimethylamino)phenyl)-1*H*-pyrrolo[1,2-c]imidazol-1-ylidene)-1-phenyl-3-tosylpropan-1-one (**3g**)

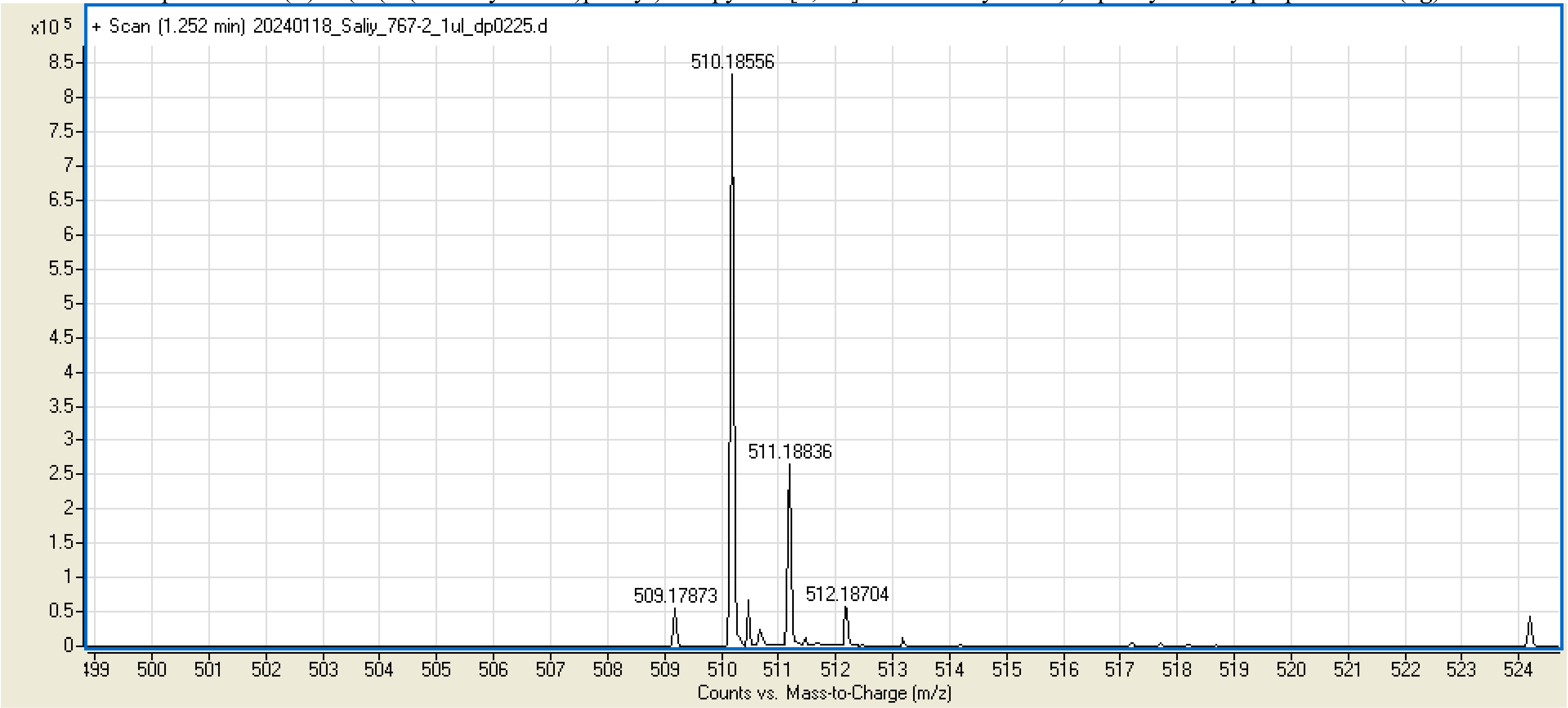

^{1}H NMR spectrum of (*E*)-2-(5-(4-fluorophenyl)-1*H*-pyrrolo[1,2-c]imidazol-1-ylidene)-1-phenyl-3-tosylpropan-1-one (**3h**) in $CDCl_3$.

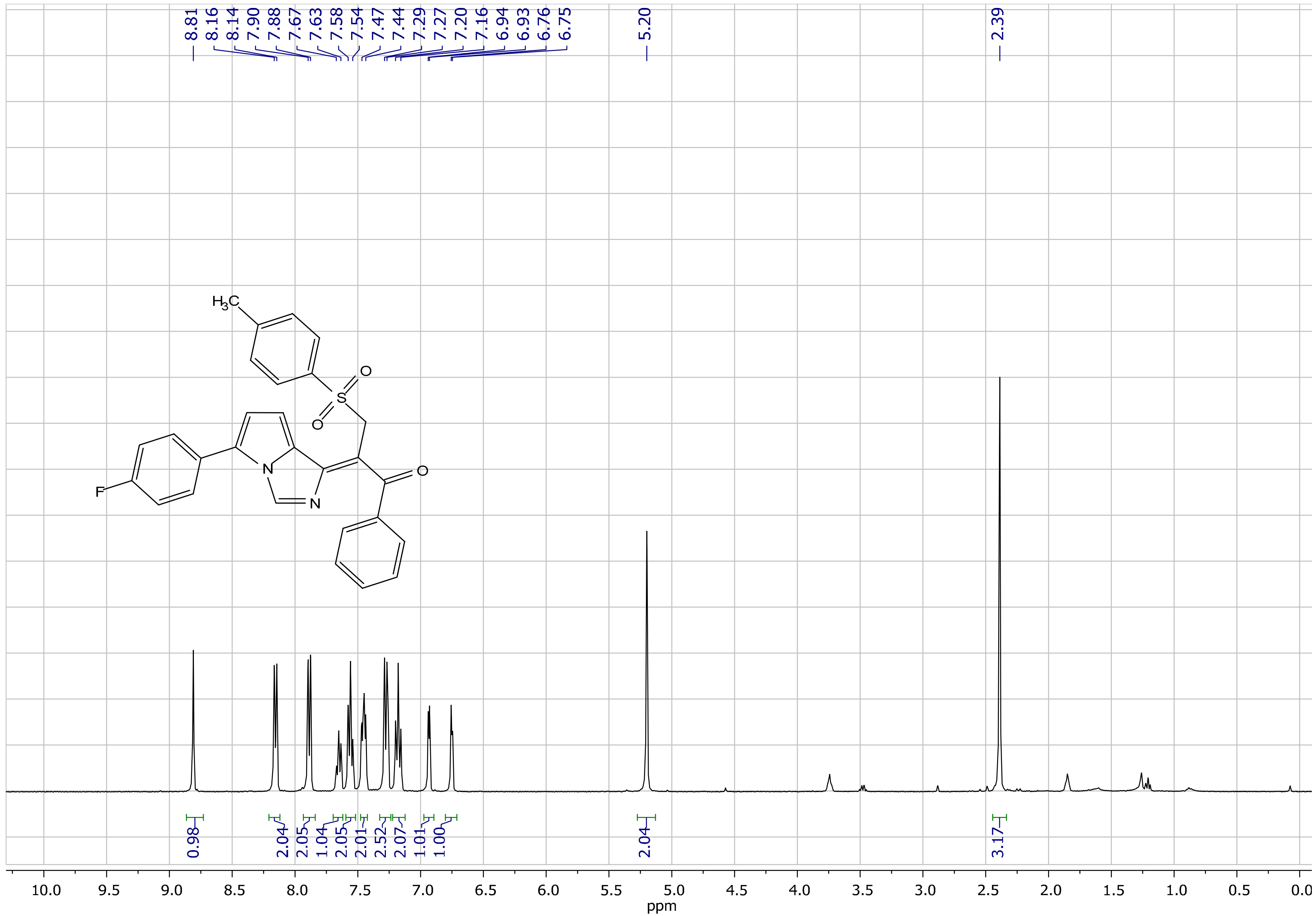

^{13}C NMR spectrum of (*E*)-2-(5-(4-fluorophenyl)-1*H*-pyrrolo[1,2-c]imidazol-1-ylidene)-1-phenyl-3-tosylpropan-1-one (**3h**) in $CDCl_3$.

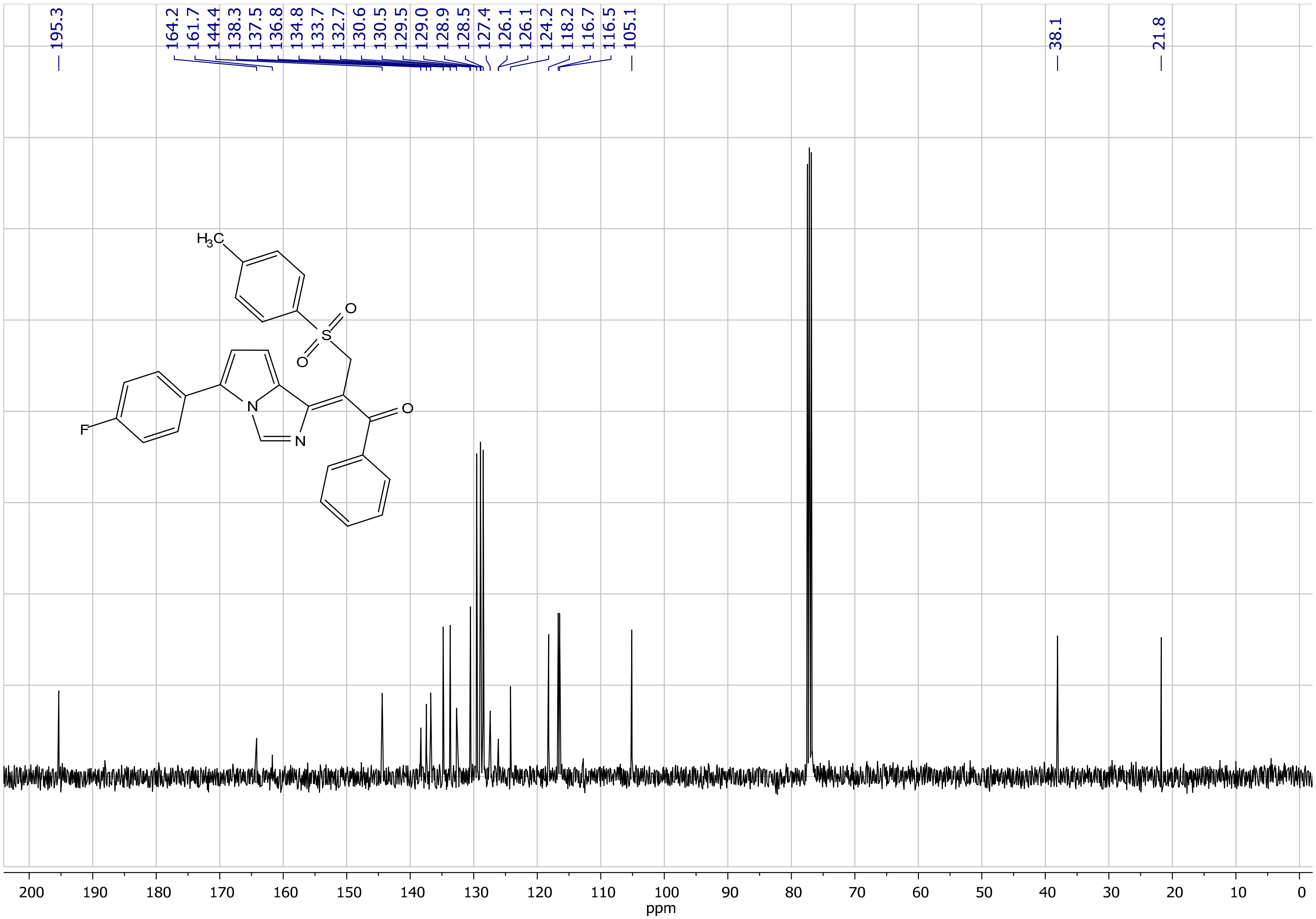

HRMS spectrum of (*E*)-2-(5-(4-fluorophenyl)-1*H*-pyrrolo[1,2-c]imidazol-1-ylidene)-1-phenyl-3-tosylpropan-1-one (**3h**)

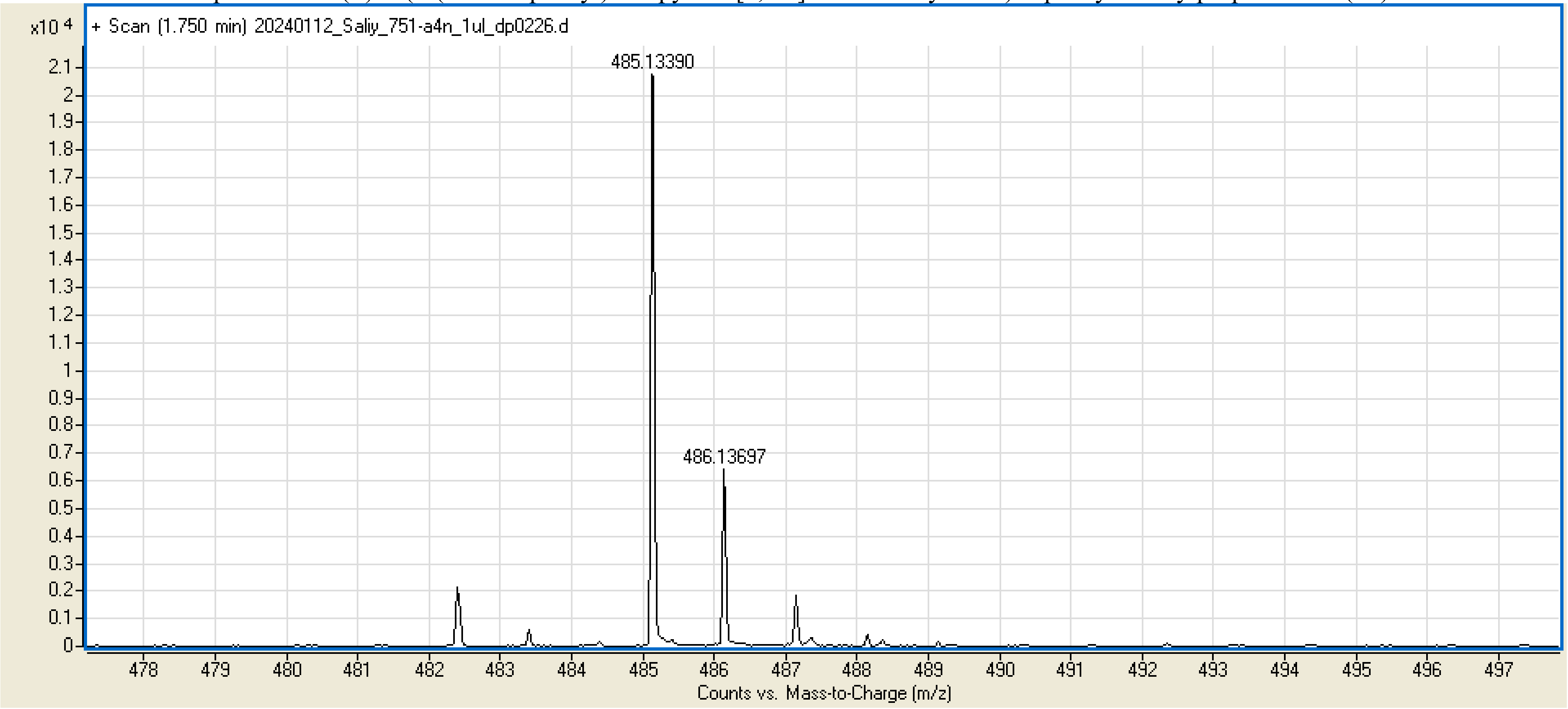

^{1}H NMR spectrum of (*E*)-2-(5-(4-fluorophenyl)-1*H*-pyrrolo[1,2-c]imidazol-1-ylidene)-1-(furan-2-yl)-3-tosylpropan-1-one (**3i**) in $CDCl_3$.

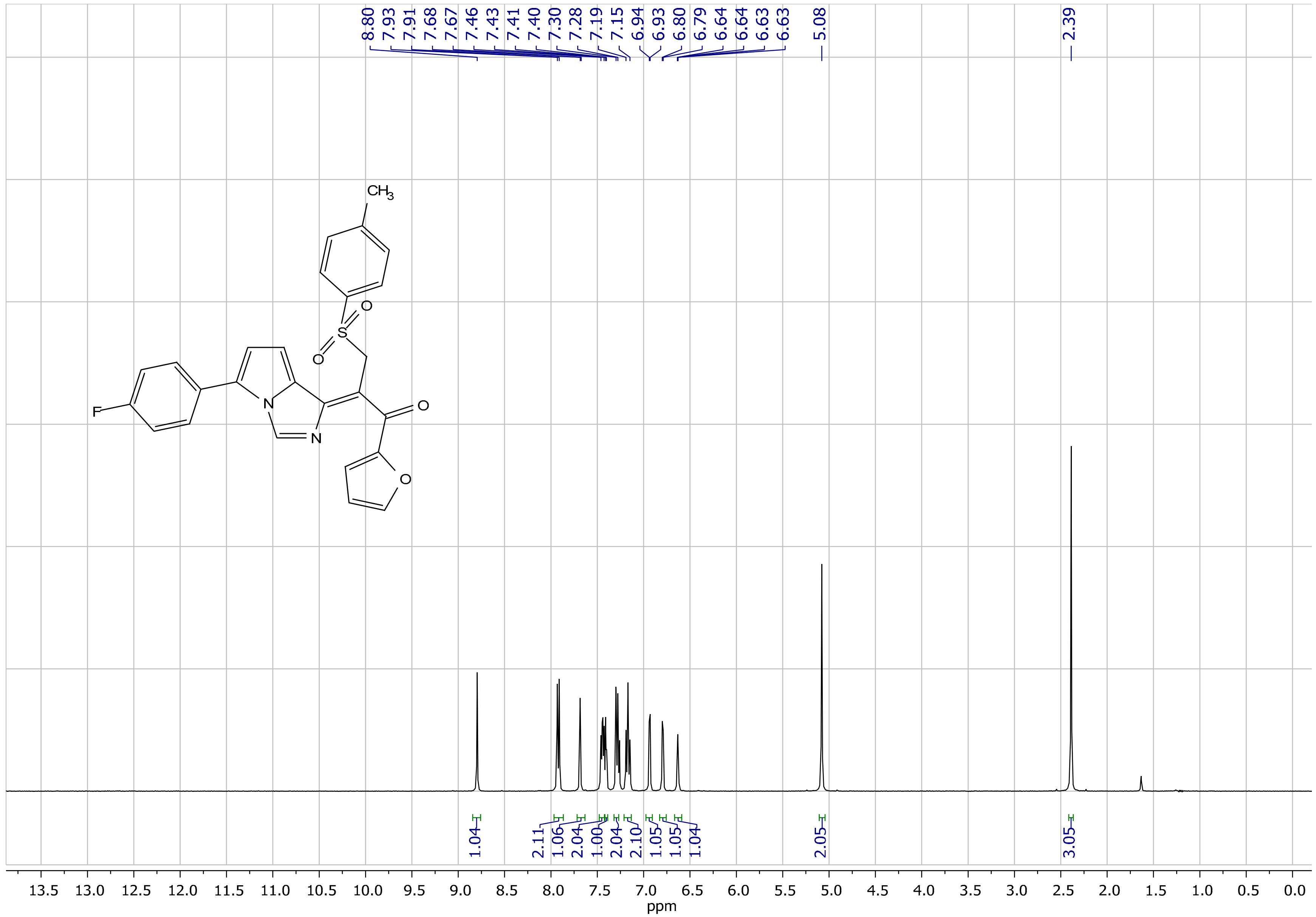

^{13}C NMR spectrum of (*E*)-2-(5-(4-fluorophenyl)-1*H*-pyrrolo[1,2-c]imidazol-1-ylidene)-1-(furan-2-yl)-3-tosylpropan-1-one (**3i**) in $CDCl_3$.

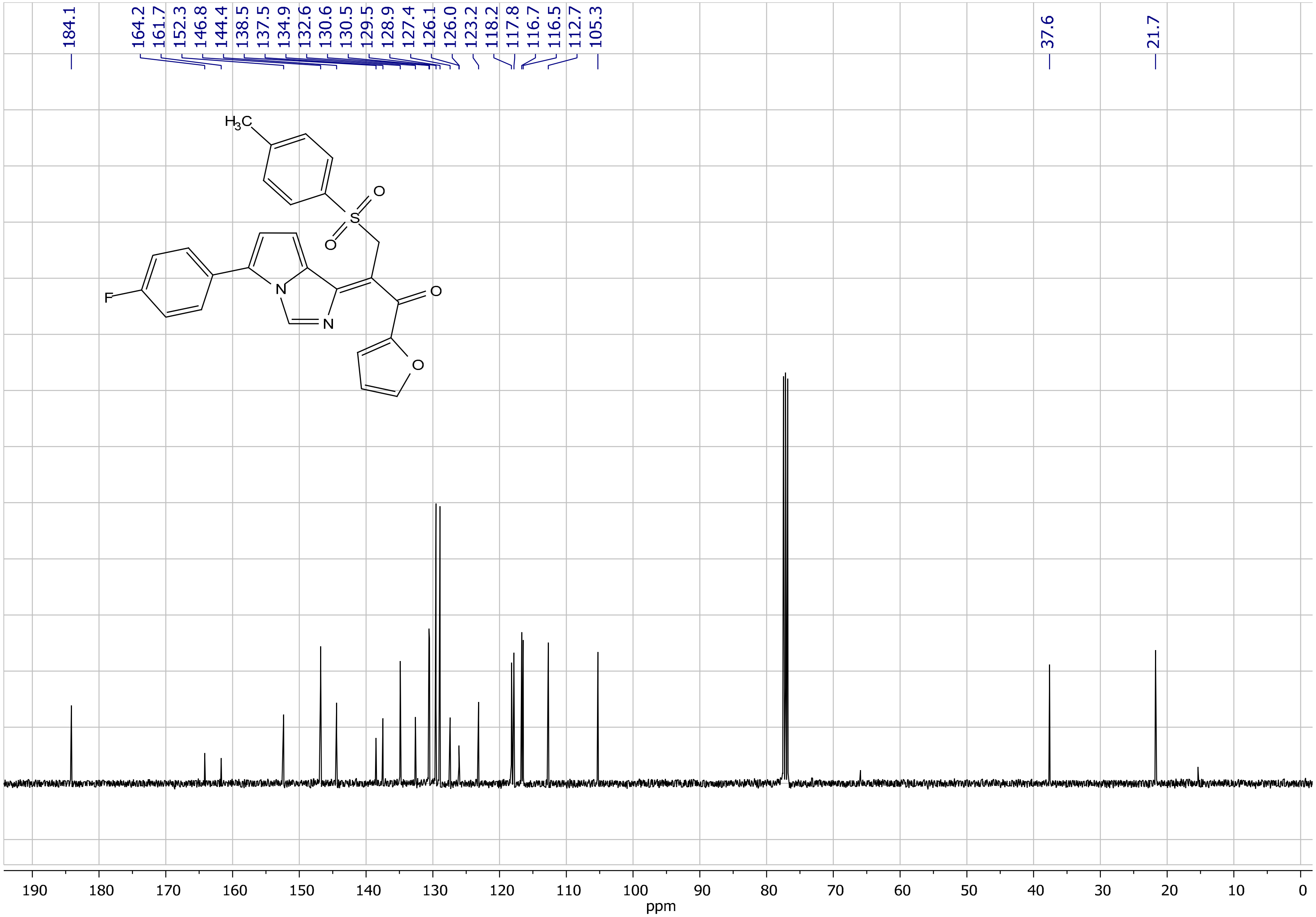

HRMS spectrum of (*E*)-2-(5-(4-fluorophenyl)-1*H*-pyrrolo[1,2-c]imidazol-1-ylidene)-1-(furan-2-yl)-3-tosylpropan-1-one (**3i**)

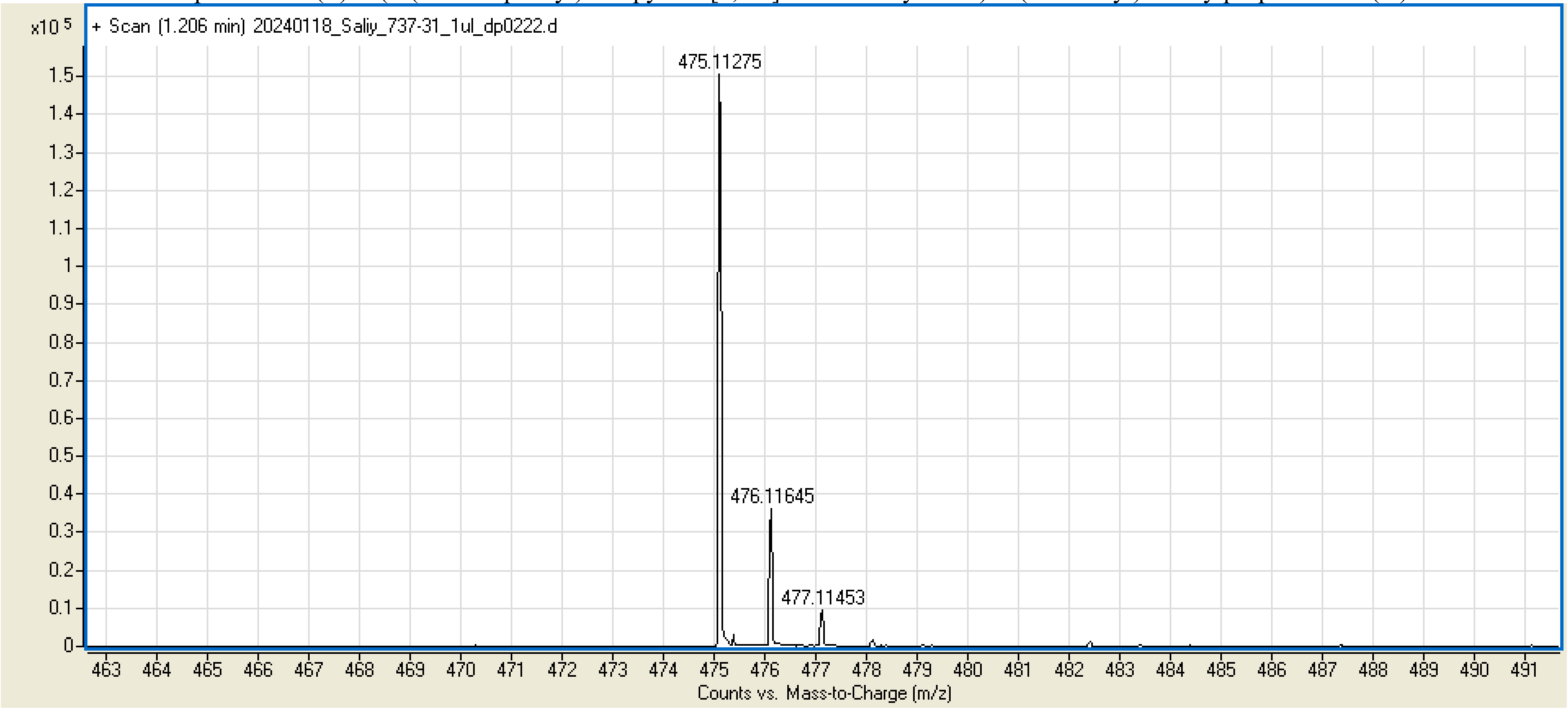

^{1}H NMR spectrum of (*E*)-2-(5-(4-chlorophenyl)-1*H*-pyrrolo[1,2-c]imidazol-1-ylidene)-1-phenyl-3-tosylpropan-1-one (**3j**) in $CDCl_3$.

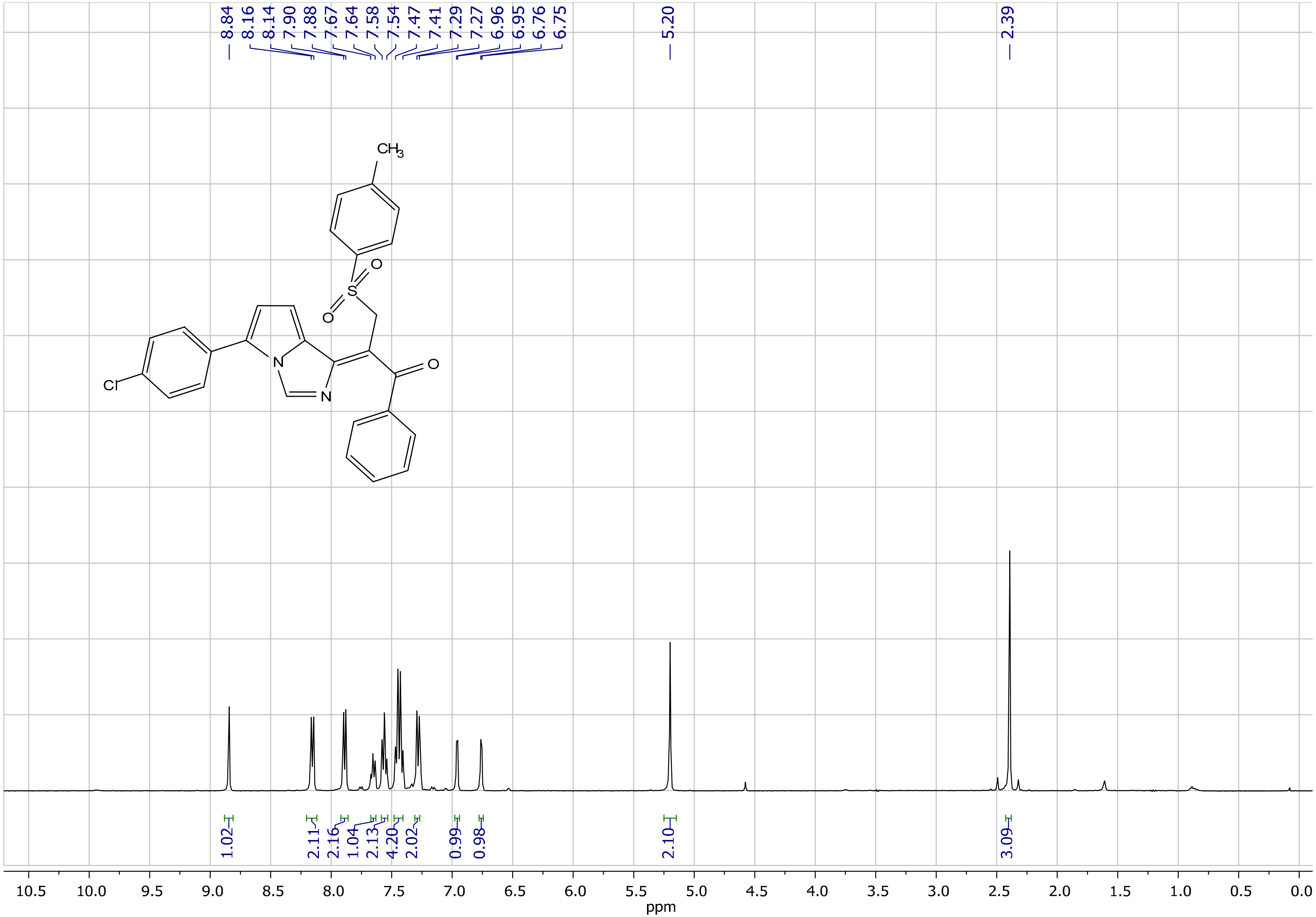

^{13}C NMR spectrum of (*E*)-2-(5-(4-chlorophenyl)-1*H*-pyrrolo[1,2-c]imidazol-1-ylidene)-1-phenyl-3-tosylpropan-1-one (**3j**) in $CDCl_3$.

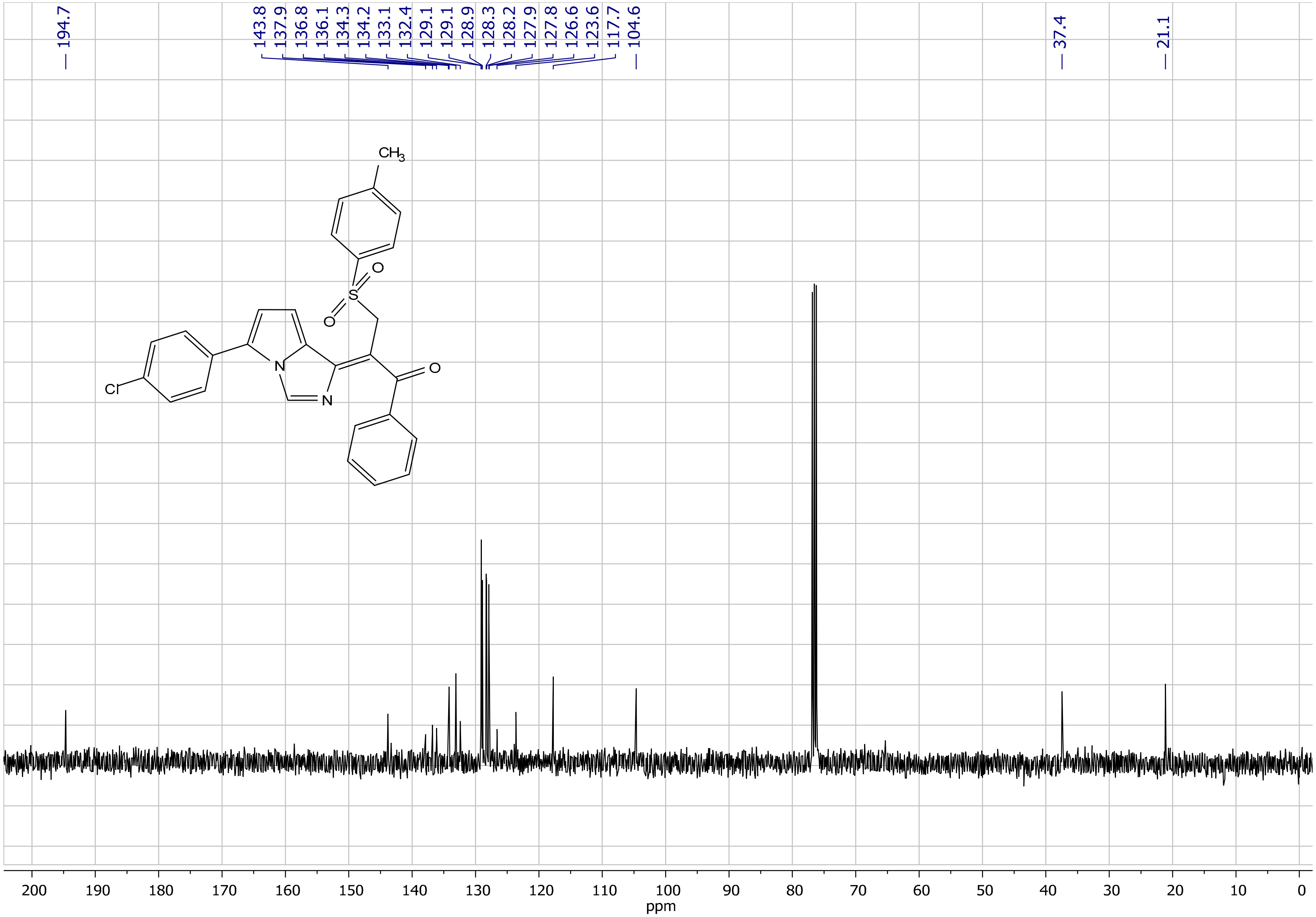

Supplement: Supplementary file 1 [file molecules-29-00885-s001.zip › molecules-2800769-supplementary File S1.pdf]
